# Single-Nucleotide Mutation Matrix: A New Model for Predicting the NF-κB DNA Binding Sites

**DOI:** 10.1371/journal.pone.0101490

**Published:** 2014-07-03

**Authors:** Wenxin Du, Jing Gao, Tingting Wang, Jinke Wang

**Affiliations:** State Key Laboratory of Bioelectronics, Southeast University, Nanjing, China; University of Granada, Spain

## Abstract

In this study, we established a single nucleotide mutation matrix (SNMM) model based on the relative binding affinities of NF-κB p50 homodimer to a wild-type binding site (GGGACTTTCC) and its all single-nucleotide mutants detected with the double-stranded DNA microarray. We evaluated this model by scoring different groups of 10-bp DNA sequences with this model and analyzing the correlations between the scores and the relative binding affinities detected with three wet experiments, including the electrophoresis mobility shift assay (EMSA), the protein-binding microarray (PBM) and the systematic evolution of ligands by exponential enrichment-sequencing (SELEX-Seq). The results revealed that the SNMM scores were strongly correlated with the detected binding affinities. We also scored the DNA sequences with other three models, including the principal coordinate (PC) model, the position weight matrix scoring algorithm (PWMSA) model and the Match model, and analyzed the correlations between the scores and the detected binding affinities. In comparison with these models, the SNMM model achieved reliable results. We finally determined 0.747 as the optimal threshold for predicting the NF-κB DNA-binding sites with the SNMM model. The SNMM model thus provides a new alternative model for scoring the relative binding affinities of NF-κB to the 10-bp DNA sequences and predicting the NF-κB DNA-binding sites.

## Introduction

NF-κB is a transcription factor (TF) identified in lymphocyte and then found to regulate the transcriptions of a large number of genes in most of cell types [1]. The NF-κB/Rel family consists of five members, including RelA/p65, c-Rel, RelB, NF-κB1/p50 and NF-κB2/p52, which can function as heterodimers or homodimers in the regulation of gene transcription [2]. The heterodimer formed by p50 and p65 is the most common functional NF-κB dimer in the mammalian cells, which regulates many important biological processes, such as immunity and inflammation [3], [4]. In this dimer, both subunits contact DNA, but only p65 contains a transactivation domain [5]. Like this dimer, other dimers formed by different members of NF-κB family can also bind the DNA-binding sites (DBSs) in the genome. Therefore, NF-κB is a well-known sequence-specific DNA-binding TF.

The consensus of the NF-κB DBSs was first identified as GGGRNNTYCC(R: G, A; Y: C, T; N: G, A, T, C)[6]. Subsequently, based on several in vitro assays, including electrophoresis mobility shift assay (EMSA), protein-binding microarray (PBM) and systematic evolution of ligands by exponential enrichment-sequencing (SELEX-Seq), this consensus was gradually expanded into GGRRNNYYCC [7]–[9] and RGGRNNHHYY (H: A, T, C) [10]. Furthermore, the analysis of the in vivo binding locations identified with the chromatin immunoprecipitation-sequencing (ChIP-Seq) revealed that NF-κB bound many of its target genes via these expanded consensuses [10], [11]. These data suggest that the DBSs of this TF have high variability. Therefore, it is still a great challenge to identify its all DBSs in the whole genome with wet experiments. In this case, the bioinformatic models for predicting DBSs would be helpful. The predicted putative DBSs provide more confident targets to the wet experiments, which can facilitate the identification of the functional DBSs of this TF in various cells [10].

In recent years, many bioinformatic models have been developed for predicting the putative DBSs of various TFs, such as position weight matrix scoring algorithm (PWMSA) [12], Match [13], TFSEARCH (http://www.rwcp.or.jp/papia), Mapper [14], and Matinspector [15]. These models can be used to predict the relative binding affinities of NF-κB to various DNA sequences by using a position weight matrix (PWM) that represents the DNA-binding motif of this TF, such as PWM M00051 NF-κB p50 in the TRANSFAC database. However, these models were not developed as the NF-κB-exclusive tool. Fortunately, one NF-κB-specific model had been developed, that is the Principal Coordinate (PC) model [8]. This model was constructed by training on the data of the relative binding affinities of NF-κB p50 homodimer to 52 variant DNA sequences representing the consensus of GGRRNNYYCC [8]. Unfortunately, this model can only be used to predict the binding affinity of NF-κB p50 to 256 DNA sequences belonging to this consensus. This limitation prevents it from more wide application in the identification of all variant potential NF-κB DBSs in the mammalian genomes. Therefore, more compatible NF-κB-specific models are still in need for this most intensively studied TF.

We have ever measured the binding affinities of NF-κB p50 homodimer to a wild-type binding site (GGGACTTTCC) and its all single-nucleotide mutants with an unimolecular double-stranded DNA (dsDNA) microarray for finding the relative importance of each position to the interaction of NF-κB with its cognate DNA sites [16]. In this paper, we confirmed these binding affinities with a newly fabricated bimolecular dsDNA microarray and constructed a single nucleotide mutation matrix (SNMM) according to the relative binding affinities measured with this wet experiment. We then applied this model to the prediction of NF-κB p50 homodimer to various DNA sequences in order to evaluate its reliability. We also compared this model with other three models, including PC, PWMSA and Match.

## Materials and Methods

### Measurement of the DNA binding affinity with DNA microarray

Pairs of complementary oligonucleotides were chemically synthesized and one was modified with amino group at the 5′ end. The paired complementary oligonucleotides were annealed in the TEN buffer (10 mM Tris-HCl, pH8.0, 100 mM NaCl, 1 mM EDTA) by incubating at 95°C for 5 min and then cooling to the room temperature gradually. The annealed double-stranded oligonucleotides were spotted (AD1500, BioDot) on the glass slides with aldehyde group (CapitalBio) to form the bimolecular dsDNA microarray. The bimolecular dsDNA microarray was incubated with the Cy3-labeled NF-κB p50 protein (Promega) by using a protocol as previously described [16]. The protein-bound dsDNA microarray was scanned with a scanner (LuxScan 10K, CapitalBio) and the fluorescent signal intensity was quantified with Image J.

### Calculation of the correlation coefficient and *p* value

The Pearson correlation coefficient (Pearson's *r*) has been widely used as a measure of the strength of linear dependence between two variables [17], [18]. Pearson's *r* ranges from -1 to +1. The negative and positive *r* values mean negative and positive correlation between two variables, respectively, and the zero *r* value means no correlation. The absolute *r* value between 0 and 0.2, 0.2 and 0.4, 0.4 and 0.6, 0.6 and 0.8, and 0.8 and 1.0 mean very weak, weak, medium, strong and very strong correlations, respectively. To demonstrate the statistical significance of the correlation coefficient, the confidence interval (*p* value) of the Pearson's *r* was calculated. The *p* values less than 0.05 and 0.01 mean that Pearson's *r* is significant and extremely significant, respectively. In this study, we calculated the Pearson's *r* and *p* value with MATLAB7.0 software.

### Evaluation of the SNMM model with the experimental data

For evaluating the SNMM model, we used three resources of NF-κB binding affinity data. The NF-κB binding affinity data measured by EMSA were collected from the previous study performed by Udalova et al [8], which detected the binding affinity of NF-κB p50 homodimer to 52 DNA sequences with the radioactive EMSA and constructed a PC model for predicting the binding affinity of NF-κB to the DNA sequences belonging to the consensus of GGRRNNYYCC. The NF-κB binding affinity data measured by PBM were collected from the previous study performed by Siggers et al [19], which detected the binding of NF-κB p50 to various DNA sequences with PBM. The NF-κB binding affinity data measured by SELEX-Seq were collected from the study performed by our lab [20], and the data was deposited into the Gene Expression Omnibus (GEO) database under the accession number of GSE:48660.

The PWMSA model was developed by Stormo et al [12]. The Match model was developed by Kel et al. [13]. When predicting the binding affinities of a transcription factor to DNA sequences by using these two models, a known PWM of the interested transcription factor is needed. To predict the binding affinities of NF-κB to DNA sequences with these two models, we employed a known PWM of NF-κB p50 constructed with 18 SELEX-selected DNA sequences by Kunsch et al [21], which was collected in transcription factor database TRANSFAC (accession number: M00051) (Table S1 in File S1). The TRANSFAC database provides an on-line Match prediction program. When predicting the binding affinity with the Match model, we input the DNA sequences to this on-line program. When predicting the binding affinity with the PWMSA model, we used a Perl script written by ourselves according to the formulas described in File S1.

### Determination of the optimal SNMM threshold for predicting the NF-κB DBSs

The optimal threshold was determined as previously described [13], [22]. To determine the optimal threshold, two groups of sequences, S1 and S2, were first selected. S1 consists of N sequences that are the NF-κB DBSs. S2 consists of M sequences that are not the NF-κB DBSs. The S1 and S2 sequences were predicted with the SNMM model. The threshold was taken from 0 to 1 at an interval of 0.001. At the threshold of *x*, if n S1 sequences were not predicted as the NF-κB DBSs, the false negative ratio (*rFN*) were [N-n]/N. Similarly, at the threshold of *x*, if m S2 sequences were predicted as the NF-κB DBSs, the false positive ratio (*rFP*) was m/M. The threshold that resulted in the lowest *rFN* and *rFP* was regarded as the optimal threshold.

### Measurement of the DNA binding affinity with EMSA

This study also detected the binding affinity of NF-κB to several DNA sequences with EMSA. Fifty five nanograms of the NF-κB p50 protein (Promega) was incubated with 1 pmol of the biotin-labeled dsDNA in a 10-μL protein binding reaction at room temperature for 1 h. The reaction was detected with an infrared fluorescence EMSA (NIRF-EMSA) exactly as previously described [23].

## Results

### Measurement of DNA-binding affinity with DNA microarray

**Figure 1:**
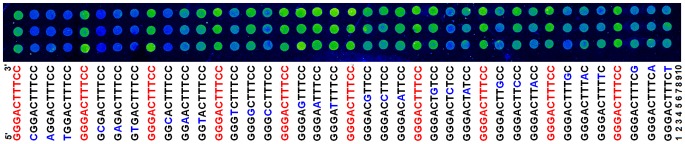
Detection of the DNA-binding affinities of the NF-κB p50 homodimer to a wild-type binding site (GGGACTTTCC) and its all single-nucleotide mutants with the bimolecular dsDNA microarray. The florescence image is a representative result of the dsDNA microarray detections. Each feature is composed of a bimolecular dsDNA probe. The sequences of the sense strands of each dsDNA probes are displayed under the image. All probes were arrayed in triplicate (each column) and the wild-type probe was arrayed in triplicate before the three mutants of each position. The positions of NF-κB binding sites were labeled as numbers of 1 to 10 after the bases.

The relative binding affinities of the NF-κB p50 homodimer to a wild-type binding site (GGGACTTTCC) [1], [24] and its all possible single nucleotide mutants were detected with the bimolecular dsDNA microarray. A representative fluorescent image of the dsDNA microarray detections is shown in Figure 1. The signal intensities of fluorescence images were quantified with the Image J software and the results were shown in Table S2 in File S1. These relative binding affinity data were in agreement with those we previously measured with the unimolecular dsDNA microarrays [16]. The bimolecular dsDNA microarray differs from the unimolecular dsDNA microarray at the DNA probe structure as previously described [25], [26].

### Construction of the SNMM model

The single-nucleotide mutant matrix (SNMM) was constructed according to the method previously described by Veprintsev et al. [27]. All binding affinities were normalized by subtracting the signal intensity values (Table S2 in File S1) with that of the reference sequence (GGGACTTTCC). As a result, a 4×10 matrix was obtained (Table 1).

**Table 1:** The single-nucleotide mutant matrix (SNMM).

	1	2	3	4	5	6	7	8	9	10
A	−31.69	−36.06	−21.42	0^a^	4.67	−26.26	−32.82	−27.38	−14.68	−28.07
C	−32.96	−40.34	−33.63	−32.27	0^a^	−33.02	−28.05	8.13	0^a^	0^a^
G	0^a^	0^a^	0^a^	−3.12	3.03	−27.83	−20.37	−33.97	−29.88	−28.17
T	−30.00	−32.43	−6.66	−24.15	5.74	0^a^	0^a^	0^a^	−23.25	−32.61

a Base of the reference sequence (GGGACTTTCC). 1 to 10, base position in the 10-bp NF-κB DBS.

The SNMM score of a particular sequence was calculated according to the following equation:
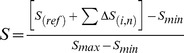



### Where

*S*_(*ref*)_ is the normalized binding affinity value of the reference sequence (0);

*S*_(*i,* n)_ is the score of base *i* at the position *n* in SNMM of a sequence;

∑Δ*S*_(*i,* n)_ is the sum of scores of bases at each position in SNMM of a sequence;

*S*_min_ is the sum of the lowest scores at each position in SNMM;

*S*_max_ is the sum of the highest scores at each position in SNMM.

### Evaluation of the SNMM model with the experimental data

#### Evaluating the SNMM model with the EMSA-measured data

**Figure 2:**
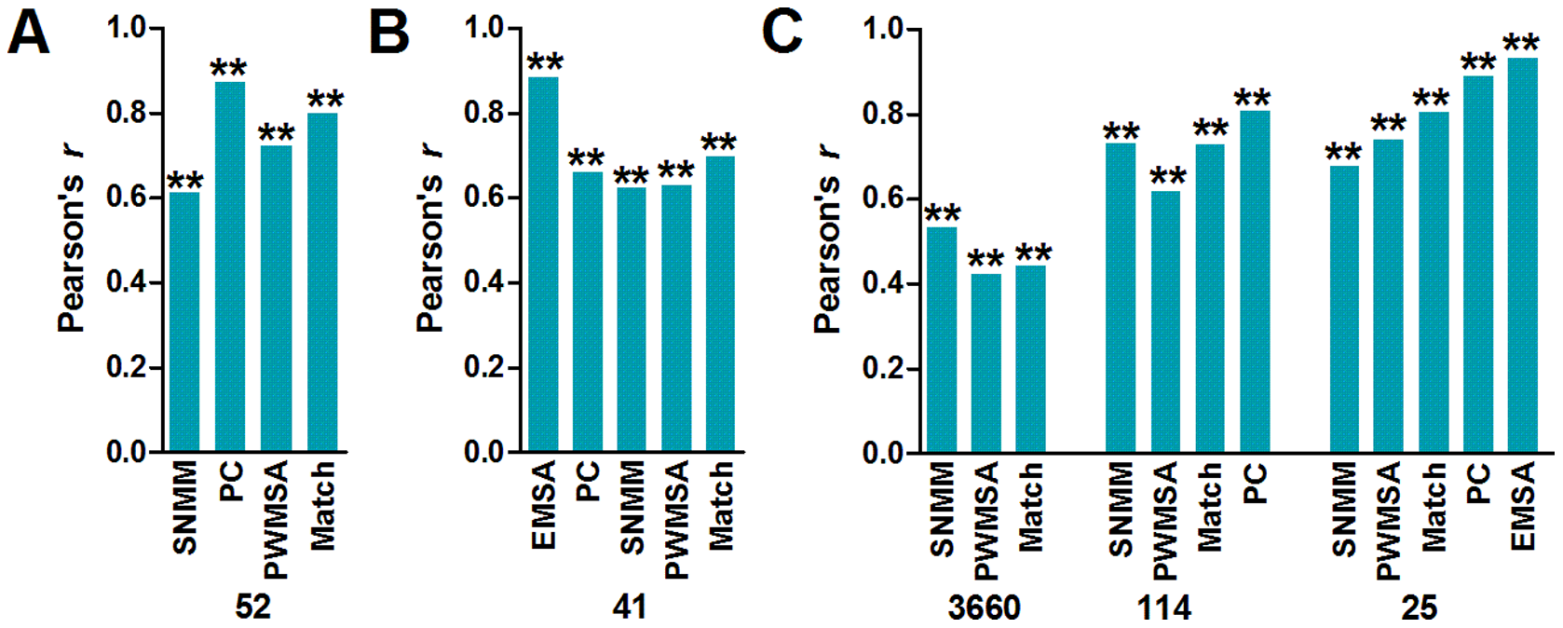
Correlation analysis. A, Correlations between the EMSA values and the scores of the PC, SNMM, PWMSA, and Match models, respectively. B, Correlations between the PBM *z* scores and the scores of the PC, SNMM, PWMSA, and Match models, respectively. C, Correlations between the SELEX-Seq values and the scores of the SNMM, PWMSA, Match, and PC models, respectively. Correlation between the SELEX-Seq values and the EMSA values. **, *p*<0.01. P value refers to the confidence interval of Pearson's *r*. The number under the abscissa refers to the number of values or sequences used in the corresponding correlation analysis.

Udalova et al. measured the relative binding affinity of the NF-κB p50 homodimer to 52 DNA sequences with EMSA [8] (Table 2). To evaluate the SNMM model, we scored these sequences with the SNMM, PWMSA and Match models, respectively. For the convenience of calculation, we converted the EMSA measured values of these sequences into the log values (named EMSA value hereafter). The EMSA values and the model scores are shown in Table 2. We also collected the PC scores of these sequences from the published data [8] and included in Table 2. With these data, we analyzed the correlation between the EMSA values and the scores obtained by various models. The results are shown in Figure 2A. It reveals that the SNMM scores are strongly correlated with the EMSA values (Pearson's *r*: 0.61); however, in comparison with other models, the SNMM scores are least correlated with the EMSA values.

**Table 2:** The EMSA values and model scores of NF-κB to 52 sequences.

Sequence	EMSA	PC	SNMM	PWMSA	Match	PBM
GGGGATTCCC	2.704	0.992	0.987	1	1	8.230
GGGGAATCCC	2.614	0.977	0.903	0.923	0.925	7.639
GGGGCTTCCC	2.444	0.981	0.972	0.979	0.923	5.197
GGGGTTCCCC	2.356	0.957	0.901	0.977	0.915	5.970
GGGGGATTCC	2.336	0.856	0.872	0.815	0.766	6.572
GGGAATTTCC	2.328	0.641	0.971	0.967	0.845	5.870
GGGGTTTTCC	2.322	0.953	0.964	0.958	0.846	5.964
GGGAAATTCC	2.258	0.633	0.888	0.885	0.77	6.192
GGGGACTTCC	2.155	0.762	0.856	0.904	0.848	4.773
GGGATCTCCC	2.057	0.813	0.895	0.881	0.771	4.350
GGGGAGTCCC	1.991	0.934	0.898	0.99	0.94	4.831
GGGGGCTCCC	1.929	0.684	0.877	0.834	0.843	3.640
GGGGGCTTCC	1.826	0.68	0.851	0.815	0.766	4.460
GGGATACCCC	1.799	0.918	0.828	0.881	0.763	2.892
GGGGAGCCCC	1.756	0.926	0.81	0.99	0.932	3.613
GGGGACCCCC	1.748	0.945	0.793	0.929	0.917	3.675
GGGATGTCCC	1.732	0.793	0.912	0.949	0.786	3.914
GGGGCTCCCC	1.69	0.922	0.883	0.979	0.915	3.613
GGGAACTTCC	1.663	0.492	0.866	0.885	0.77	4.036
GGAATACCCC	1.653	0.875	0.76	0.754	0.63	3.370
GGGATATCCC	1.623	0.828	0.917	0.881	0.771	4.179
GGGGTATCCC	1.602	0.914	0.907	0.901	0.848	2.892
GGGGCACCCC	1.491	0.77	0.8	0.902	0.84	2.010
GGGGCCCCCC	1.477	0.695	0.778	0.902	0.84	2.917
GGGAGGCCCC	1.447	0.75	0.814	0.881	0.772	2.420
GGGACTCTCC	1.431	0.273	0.867	0.941	0.76	3.406
GGGGGCCCCC	1.431	0.664	0.788	0.833	0.835	\*
GGAGAACCCC	1.415	0.621	0.747	0.796	0.784	2.214
GGGGGGCTCC	1.301	0.391	0.779	0.881	0.772	3.170
GGAGGGTTCC	0.954	0.031	0.8	0.755	0.648	\
GGAAGGCCCC	0.903	0.516	0.746	0.755	0.639	1.856
GGAAATTTCC	0.778	0.184	0.903	0.835	0.712	3.278
GGAACGCCCC	0.778	0.59	0.737	0.823	0.645	2.326
GGAGCGCCCC	0.778	0.332	0.727	0.843	0.722	\
GGGAGCTCCC	0.699	0.637	0.887	0.815	0.766	3.079
GGGGTACTCC	0.699	0.484	0.792	0.881	0.763	2.131
GGGGTGCTCC	0.699	0.422	0.787	0.948	0.777	\
GGAGGATCCC	0.602	0.16	0.83	0.707	0.71	\
GGAACGTCCC	0.477	0.316	0.826	0.824	0.653	2.453
GGAAGCTTCC	0.477	0.004	0.793	0.669	0.556	\
GGAATTCTCC	0.477	0.199	0.817	0.812	0.627	1.062
GGGAGTCTCC	0.477	0.301	0.877	0.872	0.755	2.378
GGGATCCTCC	0.477	0.156	0.781	0.862	0.685	\
GGAACCTCCC	0.301	0.383	0.809	0.757	0.638	0.950
GGAACTTTCC	0.301	0.039	0.888	0.814	0.635	1.332
GGAAGGCTCC	0.301	0.012	0.721	0.735	0.562	\
GGAAGTTTCC	0.301	0.09	0.898	0.746	0.63	0.946
GGAGGCTCCC	0.301	0.121	0.809	0.707	0.71	\
GGGAGACTCC	0.301	0.258	0.793	0.795	0.68	1.960
GGAATATTCC	0	0.008	0.823	0.735	0.561	1.495
GGAGACCTCC	0	0.078	0.699	0.777	0.706	\
GGAGGCCTCC	0	0	0.694	0.687	0.624	\

* No *z* score in the referenced article.

#### Evaluating the SNMM model with the PBM-measured data

Siggers et al. detected the relative binding affinities of the NF-κB p50 homodimer to various DNA sequences with PBM [19]. The binding affinity was presented as *z* score. The high *z* score means the high binding affinity. In comparison with the PBM-detected sequences, only 41 EMSA-detected sequences (Table 2) had the *z* scores (PBM in Table 2). We first analyzed the correlation between the PBM *z* scores and the EMSA values. The results reveal that the EMSA values are very strongly correlated with the PBM *z* scores (Pearson's *r*: 0.884) (Figure 2B), indicating that two wet experiments obtained the coincident results. We then analyzed the correlation between the PBM *z* scores and the scores obtained with four models. The results reveal that the SNMM scores are strongly correlated with the PBM *z* scores (Pearson's *r*: 0.624) (Figure 2B). The scores obtained with other models are also strongly correlated with the PBM *z* scores (Figure 2B). Therefore, the SNMM scores are strongly correlated with both the EMSA- and PBM-measured data.

#### Evaluating the SNMM model with the SELEX-Seq-measured data

Recently, we detected the relative binding affinities of the NF-κB p50 homodimer to all 10-mer DNA sequences with SELEX-Seq [20]. We obtained the SELEX-Seq fold enrichment value (name SELEX-Seq value hereafter) of the selected sequences after a four-round selection. The SELEX-Seq value reflects the relative binding affinity of the NF-κB p50 homodimer to a sequence. After a four-round selection, we obtained 7,282,890 10-mer reads that contained 242,957 variant 10-mer sequences [20].

To evaluate the SNMM model with the SELEX-Seq values, we scored all these 10-mer sequences with the SNMM model and found that there were 3660 sequences with the SNMM score ≥0.747 (a threshold for predicting NF-κB DBSs with SNMM, see below) (Table S3 in File S1). We then scored these sequences with the PWMSA and Match models (Table S3 in File S1) and analyzed the correlations between the SELEX-Seq values with the SNMM, PWMSA and Match scores, respectively. The results show that there are medium correlations between the SELEX-Seq values and the scores of three models; however, the SNMM scores are better correlated with the SELEX-Seq values than the PWMSA and Match scores (Figure 2C).

Among the 3660 sequences, we found that 114 sequences had the PC scores (Table S4 in File S1). We analyzed the correlation between the SELEX-Seq values of these 114 sequences and their SNMM, PWMSA, Match and PC scores (Figure 2C), respectively. The results reveal that the PC scores are very strongly correlated with the SELEX-Seq values. The SNMM and Match scores are similarly strongly correlated with the SELEX-Seq values. The PWMSA scores are also strongly correlated with the SELEX-Seq values; however, they are least correlated with the SELEX-Seq values in comparison with other three models.

Among the 114 sequences, we also found that 25 sequences had the EMSA values (Table S5 in File S1). We analyzed the correlations between the SELEX-Seq values of these sequences and their EMSA values and SNMM, PWMSA, Match and PC scores (Figure 2C), respectively. The results demonstrate that the SELEX-Seq values are highly correlated with the EMSA values, suggesting that two wet experiments obtained similarly reliable results. The Match and PC scores are very strongly correlated with the SELEX-Seq values. The SNMM and PWMSA scores are strongly correlated with the SELEX-Seq values.

#### Confirming the SNMM predictions with NIRF-EMSA

**Figure 3:**
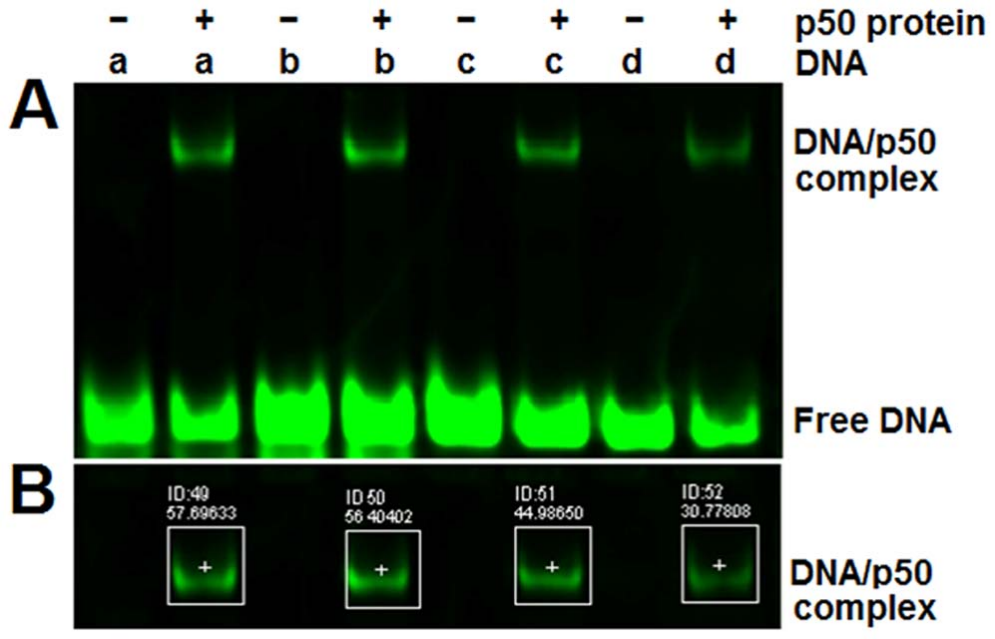
Detection of the DNA-binding affinities of the NF-κB p50 homodimer to four sequences with NIRF-EMSA. A, A representative image of the NIRF-EMSA detections. B, The quantified signal intensities of the shifted bands (labeled as DNA/p50 complex in Image A). a, GGGGATTCCC; b, GGGATCTCCC; c, GGGATACCCC; d, GGGAGGCCCC.

**Figure 4:**
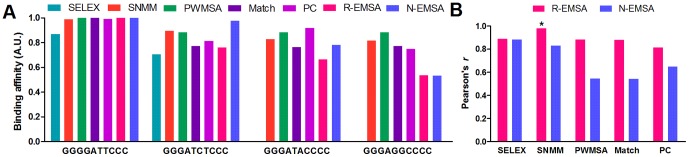
The relative binding affinities of the NF-κB p50 homodimer to four variant sequences. A, The binding affinities of the NF-κB p50 homodimer to four sequences detected with the radioactive EMSA (R-EMSA), NIRF-EMSA (N-EMSA) and SELEX-Seq (SELEX), respectively, and scored with the SNMM, PWMSA, Match and PC models, respectively. B, The correlation between the EMSA-detected values and the model scores. *, *p*<0.05; no *, *p*>0.05. P value refers to the confidence interval of Pearson's *r*.

To further evaluate the SNMM model, we selected four DNA sequences with various SELEX-Seq values and detected them with NIRF-EMSA (N-EMSA). The results are shown in Figure 3. The signal intensity of the shifted bands resulted from the DNA/p50 complex were quantified and normalized to the highest signal intensity (Figure 3). We then scored these sequences with the SNMM, PWMSA and Match models, respectively. We also collected the PC scores and the EMSA values of these sequences because they had been previously scored by the PC model and detected by the radioactive EMSA (R-EMSA) (Figure 4) [8]. Finally, we analyzed the correlations between the N- and R-EMSA values and the SNMM, PWMSA, Match and PC scores, respectively. The results reveal that the SELEX-Seq values are very strongly correlated with both the N-EMSA and R-EMSA values, demonstrating that the SELEX-Seq value can reflect the relative binding affinity of NF-κB to a sequence. The results also demonstrate that the SNMM scores are very strongly correlated with both the N-EMSA and R-EMSA values, suggesting that the SNMM score can be used as an indicator of the relative binding affinity of NF-κB to a 10-mer DNA sequence.

#### Determining the threshold of SNMM for predicting the NF-κB DBSs

To predict the NF-κB DBSs with the SNMM model, an optimal threshold should be determined. For this end, we first set up two groups of sequences. From 52 EMSA-detected DNA sequences (Table 2), we selected 30 sequences with the highest EMSA values as S1 group (Table S6 in File S1). These sequences were regarded as the DBSs of NF-κB because most of them (93.3%) had the Match score over 0.75 that is the common threshold of this model. We also selected 30 sequences from random 10-mer sequences that had the Match score of zero as S2 group (Table S6 in File S1). These sequences were regarded as the non-DBSs of NF-κB. By using the methods described in Methods and Materials, we identified 0.747 as the optimal threshold of SNMM for predicting the NF-κB DBSs.

## Discussion

In the evaluations of the SNMM model with the experimental data (Figure 2), it can be seen that the PC scores are more strongly correlated with the experimental data than the scores of other models. The reason lies in two aspects. First, the PC model was trained on the binding affinity data of the NF-κB p50 homodimer to 52 variant DNA sequences representing the consensus of GGRRNNYYCC [8]. When analyzing the correlations between the PC scores and the experimental data, we used the sequences with the PC scores, including 52, 41 and 114 sequences detected by EMSA, PBM and SELEX-Seq, respectively. Of course, these sequences belong to the consensus of GGRRNNYYCC. Second, the PC model was constructed as a mathematical model based on algorithm [8], which takes account of the correlation between nucleotides at different positions in its construction. However, PWMSA and Match are the PWM-based models. The PWM-based models are based on the additivity assumption, which assumes that the nucleotides in a transcription factor binding site are independent. The SNMM model also is a matrix-based model, which takes no account of the correlation between the nucleotides at the different positions in its construction. Therefore, in the evaluations of the SNMM model with the sequences belonging to GGRRNNYYCC, the matrix-based models are not as good as the PC model. However, the PC model can only be used to score 256 sequences belonging to GGRRNNYYCC, but the SNMM model is enabled to score any sequence. For example, we scored 242,957 variant SELEX-Seq-selected 10-mer sequences in this study and found 3660 sequences with the SNMM score ≥0.747.

In this study, the PWMSA and Match scoring used a PWM that was constructed with 18 SELEX-selected sequences [21] (Table S1 in File S1). Like 52 sequences (Table 1) that were used to train the PC model, these sequences also belong to the consensus of GGRRNNYYCC, which is identified as the canonical NF-κB DNA-binding motif [7]–[9]. However, most sequences used to construct the SNMM model do not belong to this consensus, such as AGGACTTTCC and GGGACTTTCA. Therefore, in the evaluations of the SNMM models with 52 EMSA-detected and 25 SELEX-Seq detected sequences belonging to the consensus of GGRRNNYYCC, the PWMSA and Match scores are more strongly correlated with the experimental data than the SNMM scores. However, in the evaluations of the SNMM models with 41 PBM-detected and 114 SELEX-Seq-detected sequences belonging to the consensus of GGRRNNYYCC, the SNMM scores showed the similar correlations with the experimental data as the PWMSA and Match scores. Furthermore, in the evaluations of the SNMM model with 3660 SELEX-Seq detected sequences, the SNMM scores are more strongly correlated with the experimental data than the PWMSA and Match scores. It should be noted that most of these 3660 sequences are not the canonical NF-κB DBSs belonging to the consensus of GGRRNNYYCC. These results reveal that the SNMM model is not only qualified to identify the canonical NF-κB DBSs as other models, but also more qualified to identify the noncanonical NF-κB DBSs than other models.

In this study, when analyzing the correlations of the SNMM, PWMSA and Match scores with the SELEX-Seq values of the 3660 sequences, we found that all correlations did not reach the strong level (Figure 2C). This results from the complex constitution of the SELEX-Seq-selected sequences. SELEX-Seq is an unbiased *in vitro* selection technique that can be used to find any DNA binders of a transcription factor [28]–[30]. For example, Wong et al. studied the DNA-binding specificity of NF-κB with the technique and found that NF-κB could bind both canonical and noncanonical sequences [10]. However, the PWM used in the PWMSA and Match models was established only with the canonical sequences. Although the construct of the SNMM model used some noncanonical sequences, the reference sequence and most its mutated sequences used in SNMM also belong to the canonical sequences. This suggests that it is important to take the noncanonical sequences into account in establishing more accurate models for predicting the NF-κB DBSs. The binding affinity data obtained with the unbiased *in vitro* detection techniques such as SELEX-Seq [10] and universal PBM [19] would be important to this end.

This study demonstrated that SNMM provides a simple model for predicting the NF-κB DBSs. It is worthy to mention that several new studies also revealed that simple models based on mononucleotide PWM are effective in evaluating the DNA-binding specificities of TFs. For example, Jolma et al. systematically analyzed specificities of 303 human DNA-binding domains (DBDs), 84 mouse DBDs, and 151 human full-length TFs that represent 411 different TFs using a high-throughput SELEX (HT-SELEX), they found that the vast majority of interactions that occur between a TF and the individual DNA bases are independent of each other [31]. Weirauch et al. systematically evaluated 26 methods for modeling TF sequence specificity using the *in vitro* PBM data of 66 mouse TFs belonging to various families, the results indicated that the simple models based on mononucleotide PWM trained by the best methods performed similarly to more complex models for most of TFs examined [32]. Therefore, the approach that was used to establish the NF-κB-specific SNMM model in this study may be used to construct the similar SNMM models for other TFs.

In conclusion, we constructed a new simple model for predicting the NF-κB DBSs and verified its effectiveness with various resources of experimental data in this study.

## Supporting Information

File S1 Supporting methods and Tables. Supporting methods. Construction of PWMSA. Table S1. The TRANSFAC NF-κB MA00051 PWM. Table S2. The binding affinities of NF-κB p50 homodimer to the sequence GGGACTTTCC and its all single-nucleotide mutants detected with the dsDNA microarray. Table S3. The SELEX-Seq values and model scores of 3660 sequences. Table S4. The SELEX-Seq values and model scores of 114 sequences. Table S5. The SELEX-Seq values and model scores of 25 sequences. Table S6. The sequences used to determine the optimal threshold of SNMM.
(DOCX)
