# Supplementary material for: Single-Nucleotide Mutation Matrix: A New Model for Predicting the NF-κB DNA Binding Sites
Source: PLoS One. 2014 Jul 3;9(7):e101490. doi: 10.1371/journal.pone.0101490 (PMC4081663; doi:10.1371/journal.pone.0101490)
Supplement: File S1 — Supporting methods and Tables. Supporting methods. Construction of PWMSA. Table S1. The TRANSFAC NF-κB MA00051 PWM. Table S2. The binding affinities of NF-κB p50 homodimer to the sequence GGGACTTTCC and its all single-nucleotide mutants detected with the dsDNA microarray. Table S3. The SELEX-Seq values and model scores of 3660 sequences. Table S4. The SELEX-Seq values and model scores of 114 sequences. Table S5. The SELEX-Seq values and model scores of 25 sequences. Table S6. The sequences used to determine the optimal threshold of SNMM. (DOCX) [file pone.0101490.s001.docx]

**File S1 Supporting methods and Tables**

**Supporting methods**

*Construction of PWMSA.*

The PWMSA is constructed as the following procedures.

1. Construction of the count matrix

The count matrix (CM) is constructed by first aligning N sequences and then counting the numbers of four bases (A, C, G and T) at each position. Count Matrix is displayed as formula (1):

$CM=\left\{\begin{matrix} m_{1,A} & m_{2,A} & \ldots& m_{n,A} \\ m_{1,C} & m_{2,C} & \ldots& m_{n,C} \\ m_{1,G} & m_{2,G} & \ldots& m_{n,G} \\ m_{1,T} & m_{2,T} & \ldots& m_{n,T} \end{matrix}\right\}$ (1)

1. Construction of PFM

For the convenience of calculation, the CM is changed into position frequency matric (PFM) by using the equation (2):

$f_{i,b}=\frac{n_{i,b}}{\sum_{i=1}^{b} n_{i,b}}$ (2)

Where

*b*, four bases (A, C, G and T);

*n_i,b_*, number of base *b* at position *i*;

*f _i,b_*, frequency of base *b* at position *i*.

In view of error resulted from small sample statistic, the equation (2) is changed into equation (3) by introducing the spurious count *k*.

$f_{i,b}^{'}=\frac{n_{i,b}+p_{i}k}{\sum_{i=1}^{A} n_{i,b}+k}$ (3)

Where

*p_i_*, the background frequency of four bases: 0.28 (A, T) and 0.22 (C, G) [[1](#_ENREF_1)];

*k*, the spurious count when the count is 0 (*k*=0.001).

1. Construction of PWM

Due to the preference of base composition of DNA sequence, the PFM is generally transferred into position weight matrix (PWM) [[2](#_ENREF_2),[3](#_ENREF_3)] by using the equation (4).

$W_{i,b}=\ln\left(\frac{f_{i,b}^{'}}{p_{i}}\right)$ (4)

According to formula (4), the PWM can be shown as formula (5).

$W=\left\{\begin{matrix} w_{1,A} & w_{2,A} & \ldots& w_{n,A} \\ w_{1,C} & w_{2,C} & \ldots& w_{n,G} \\ w_{1,G} & w_{2,G} & \ldots& w_{n,G} \\ w_{1,T} & w_{2,T} & \ldots& w_{n,T} \end{matrix}\right\}$ (5)

1. Construction of PWMSA

The sequences are scored with PWM by using the scoring function *S* (6).

$S=\frac{\sum_{i=1}^{n} M_{i}w_{ib}}{\sum_{i=1}^{n} M_{i}\max w_{i}}$ (6)

If necessary, for the convenience of calculation, the score value *S'* can be transferred into the values between 0 and 1 by using the deformation equation (7).

$S^{'}=\frac{\sum_{i=1}^{n}M_{i}w_{ib}-\sum_{i=1}^{n}M_{i}\min w_{i}}{\sum_{i=1}^{n}M_{i}\max w_{i}-\sum_{i=1}^{n}M_{i}\min w_{i}}$ (7)

Where

*M_i_*, conservative parameter. *M_i_* is positive proportional to the conservation of base, which is calculated by using the equation (8).

$M_{i}=\sum_{b} \frac{\left(f_{ib}^{''}/N-1/4\right)}{1/4}$ (8)

Where

$f_{ib}^{''}$, the frequency of base *b* at position *i*.

**Supporting tables**

Table S1. The TRANSFAC NF-κB MA00051 PWM.

| PO | A | C | G | T |  |
| --- | --- | --- | --- | --- | --- |
| 01 | 0 | 0 | 18 | 0 | G |
| 02 | 0 | 0 | 18 | 0 | G |
| 03 | 0 | 0 | 18 | 0 | G |
| 04 | 2 | 0 | 16 | 0 | G |
| 05 | 16 | 1 | 0 | 1 | A |
| 06 | 0 | 0 | 3 | 15 | T |
| 07 | 0 | 7 | 1 | 10 | Y |
| 08 | 0 | 16 | 0 | 2 | C |
| 09 | 0 | 18 | 0 | 0 | C |
| 10 | 0 | 17 | 1 | 0 | C |

PO, position.

Table S2. The binding affinities of the NF-κB p50 homodimer to the sequence of GGGACTTTCC and its all single-nucleotide mutants detected with the dsDNA microarray.

| No. of DNA | Sequence | Mutated base | Signal intensity |
| --- | --- | --- | --- |
| 00 | GGGACTTTCC | RS | 52.80 |
| 01 | **C**GGACTTTCC | C1 | 19.84 |
| 02 | **A**GGACTTTCC | A1 | 21.11 |
| 03 | **T**GGACTTTCC | T1 | 22.80 |
| 04 | G**C**GACTTTCC | C2 | 12.46 |
| 05 | G**A**GACTTTCC | A2 | 16.74 |
| 06 | G**T**GACTTTCC | T2 | 20.37 |
| 07 | GG**C**ACTTTCC | C3 | 19.17 |
| 08 | GG**A**ACTTTCC | A3 | 31.38 |
| 09 | GG**T**ACTTTCC | T3 | 46.14 |
| 10 | GGG**T**CTTTCC | T4 | 28.65 |
| 11 | GGG**G**CTTTCC | G4 | 49.68 |
| 12 | GGG**C**CTTTCC | C4 | 20.53 |
| 13 | GGGA**G**TTTCC | G5 | 55.83 |
| 14 | GGGA**A**TTTCC | A5 | 57.47 |
| 15 | GGGA**T**TTTCC | T5 | 58.54 |
| 16 | GGGAC**G**TTCC | G6 | 24.98 |
| 17 | GGGAC**C**TTCC | C6 | 19.78 |
| 18 | GGGAC**A**TTCC | A6 | 26.54 |
| 19 | GGGACT**G**TCC | G7 | 32.44 |
| 20 | GGGACT**C**TCC | C7 | 24.75 |
| 21 | GGGACT**A**TCC | A7 | 19.99 |
| 22 | GGGACTT**G**CC | G8 | 18.83 |
| 23 | GGGACTT**C**CC | C8 | 60.93 |
| 24 | GGGACTT**A**CC | A8 | 25.42 |
| 25 | GGGACTTT**G**C | G9 | 22.92 |
| 26 | GGGACTTT**A**C | A9 | 38.12 |
| 27 | GGGACTTT**T**C | T9 | 29.55 |
| 28 | GGGACTTTC**G** | G10 | 24.63 |
| 29 | GGGACTTTC**A** | A10 | 24.73 |
| 30 | GGGACTTTC**T** | T10 | 20.19 |

RS, reference sequence, which is a wild-type binding site. The mutated bases are shown in bold face in sequences. The number after the mutated base refers to the position of the mutated base in the 10-bp DNA-binding site (Figure 1).

Table S3. The SELEX-Seq values and model scores of 3660 sequences.

| Sequence | SELEX | SNMM | PWMSA | Match | Sequence | SELEX | SNMM | PWMSA | Match |
| --- | --- | --- | --- | --- | --- | --- | --- | --- | --- |
| GGGATTTCCC | 2.450 | 1.000 | 0.958 | 0.846 | GGTATTCTCG | 0.000 | 0.775 | 0.785 | 0.527 |
| GGGGATTTCC | 2.382 | 0.961 | 0.981 | 0.923 | GGGTGTTCTA | 0.000 | 0.752 | 0.583 | 0.591 |
| GGACTTTCCC | 2.357 | 0.830 | 0.758 | 0.702 | GGGATTGCTG | 0.000 | 0.772 | 0.798 | 0.587 |
| GGGGTTTCCC | 2.348 | 0.990 | 0.977 | 0.923 | GGGGCATCTC | 0.000 | 0.815 | 0.776 | 0.715 |
| GGGGATTCCC | 2.342 | 0.987 | 1.000 | 1.000 | TGGGATTCCA | 0.000 | 0.803 | 0.765 | 0.761 |
| GGGATTCCCC | 2.333 | 0.911 | 0.958 | 0.837 | GGGAATGCGG | 0.000 | 0.748 | 0.820 | 0.664 |
| GGGTTTTCCC | 2.332 | 0.923 | 0.885 | 0.835 | GGTGGGCCCC | 0.000 | 0.783 | 0.774 | 0.717 |
| GGGGTTCCCC | 2.289 | 0.901 | 0.977 | 0.915 | GGTGCTTCCA | 0.000 | 0.862 | 0.744 | 0.684 |
| GGGGTTTTCC | 2.280 | 0.964 | 0.958 | 0.846 | CGGATTTCCG | 0.000 | 0.806 | 0.804 | 0.613 |
| GGTACTTTCC | 2.262 | 0.935 | 0.814 | 0.635 | GAGTATTCCC | 0.000 | 0.806 | 0.781 | 0.779 |
| GGGTTTCCCC | 2.232 | 0.834 | 0.885 | 0.826 | TGTGAGTCCC | 0.000 | 0.782 | 0.737 | 0.674 |
| GGGGGTTTCC | 2.199 | 0.956 | 0.891 | 0.841 | GAGATTTTCC | 0.000 | 0.860 | 0.812 | 0.635 |
| GGGCTTTCCC | 2.191 | 0.898 | 0.885 | 0.835 | GGAAGTTTAT | 0.000 | 0.748 | 0.510 | 0.391 |
| GGTATTCCCC | 2.188 | 0.890 | 0.831 | 0.704 | GAGGGTTCTC | 0.000 | 0.793 | 0.657 | 0.652 |
| GTGGTTCCCC | 2.188 | 0.798 | 0.850 | 0.782 | GCGATTCCCC | 0.000 | 0.783 | 0.831 | 0.704 |
| GGGGGTTCCC | 2.180 | 0.982 | 0.911 | 0.918 | GGTGGCTCTC | 0.000 | 0.782 | 0.580 | 0.577 |
| GGTATTTCCC | 2.169 | 0.979 | 0.831 | 0.713 | GTGACTGTCC | 0.000 | 0.789 | 0.808 | 0.610 |
| GGGCTTCCCC | 2.166 | 0.809 | 0.885 | 0.826 | AGATTTTCCC | 0.000 | 0.755 | 0.631 | 0.569 |
| GGGACTTTCC | 2.165 | 0.956 | 0.941 | 0.768 | GGAGGTTCTA | 0.000 | 0.751 | 0.549 | 0.546 |
| GGGAGTTCCC | 2.158 | 0.991 | 0.891 | 0.841 | TGTATTTACC | 0.000 | 0.771 | 0.612 | 0.491 |
| GTGGTTTCCC | 2.150 | 0.887 | 0.851 | 0.790 | TGCAGTTCCC | 0.000 | 0.790 | 0.638 | 0.575 |
| GGGAATTCCC | 2.143 | 0.997 | 0.981 | 0.923 | GGAGTTTCTG | 0.000 | 0.759 | 0.697 | 0.557 |
| GTGCTTTCCC | 2.140 | 0.795 | 0.758 | 0.702 | TGGGGTGCCC | 0.000 | 0.822 | 0.777 | 0.760 |
| GGTTTTCCCC | 2.136 | 0.813 | 0.758 | 0.693 | GGTAGTCTCA | 0.000 | 0.767 | 0.637 | 0.516 |
| GGGGCTTTCC | 2.135 | 0.946 | 0.960 | 0.846 | GGTACATTTC | 0.000 | 0.778 | 0.611 | 0.428 |
| GGGGCTTCCC | 2.113 | 0.972 | 0.979 | 0.923 | GGAGTTTCAG | 0.000 | 0.786 | 0.697 | 0.557 |
| GGTGGTTTCC | 2.106 | 0.935 | 0.765 | 0.708 | GGGATCCTCC | 0.000 | 0.781 | 0.862 | 0.685 |
| GTGTTTTCCC | 2.097 | 0.821 | 0.758 | 0.702 | GGTGTTACAC | 0.000 | 0.818 | 0.690 | 0.629 |
| GGTGGTTCCC | 2.095 | 0.960 | 0.784 | 0.785 | TGTAGTTTCA | 0.000 | 0.760 | 0.510 | 0.391 |
| GGGTATTCCC | 2.088 | 0.920 | 0.908 | 0.912 | TGGACTGTCC | 0.000 | 0.796 | 0.808 | 0.610 |
| GGGATTTTCC | 2.076 | 0.974 | 0.939 | 0.768 | GGGGGATCCT | 0.000 | 0.795 | 0.725 | 0.737 |
| GTGATTCCCC | 2.072 | 0.808 | 0.831 | 0.704 | GGTAGTATCC | 0.000 | 0.840 | 0.712 | 0.602 |
| GGATTTTCCC | 2.067 | 0.856 | 0.758 | 0.702 | GGTGCTGCAC | 0.000 | 0.840 | 0.719 | 0.632 |
| GGTGCTTTCC | 2.060 | 0.925 | 0.833 | 0.713 | CGTAGGTTCC | 0.000 | 0.752 | 0.609 | 0.438 |
| GGGACTTCCC | 2.057 | 0.982 | 0.960 | 0.846 | GGTGTATTAC | 0.000 | 0.813 | 0.628 | 0.505 |
| GGATTTCCCC | 2.044 | 0.767 | 0.758 | 0.693 | TGGATTTGCC | 0.000 | 0.771 | 0.739 | 0.624 |
| GGTGTTTTCC | 2.037 | 0.943 | 0.831 | 0.713 | GGTGAGTCTC | 0.000 | 0.804 | 0.737 | 0.674 |
| GGTGATTCCC | 2.035 | 0.966 | 0.873 | 0.867 | GGAAATTTTC | 0.000 | 0.829 | 0.708 | 0.579 |
| GTGATTTCCC | 2.033 | 0.897 | 0.831 | 0.713 | GGTGATTCAT | 0.000 | 0.816 | 0.638 | 0.628 |
| GGTGATTTCC | 2.033 | 0.940 | 0.854 | 0.790 | TTGAATTTCC | 0.000 | 0.773 | 0.708 | 0.579 |
| GGGATGTCCC | 2.029 | 0.912 | 0.949 | 0.786 | GGGAATTTGA | 0.000 | 0.787 | 0.726 | 0.606 |
| GGTACTTCCC | 2.025 | 0.961 | 0.833 | 0.713 | GGTATTTGCC | 0.000 | 0.845 | 0.739 | 0.624 |
| GGGGTGTCCC | 2.016 | 0.902 | 0.968 | 0.863 | GTTATTTCCA | 0.000 | 0.787 | 0.596 | 0.474 |
| GGGGGTTTTC | 2.008 | 0.882 | 0.765 | 0.708 | TGTACGTTCC | 0.000 | 0.752 | 0.678 | 0.443 |
| GGGGTTTACC | 2.006 | 0.878 | 0.885 | 0.835 | GGGATTGCTA | 0.000 | 0.773 | 0.716 | 0.581 |
| GGTGCTTCCC | 1.998 | 0.951 | 0.853 | 0.790 | GATACTTTCC | 0.000 | 0.821 | 0.687 | 0.502 |
| GGGGGTTTTA | 1.980 | 0.793 | 0.656 | 0.602 | GGGAGCTTTC | 0.000 | 0.787 | 0.669 | 0.556 |
| GGGGATTTTC | 1.975 | 0.887 | 0.854 | 0.790 | GGGATTGCCA | 0.000 | 0.846 | 0.843 | 0.714 |
| GGGGATTTAC | 1.964 | 0.914 | 0.854 | 0.790 | CAGGTTTCCC | 0.000 | 0.771 | 0.724 | 0.657 |
| GGTGTTTCCC | 1.962 | 0.969 | 0.851 | 0.790 | TGGACTGCCC | 0.000 | 0.822 | 0.827 | 0.687 |
| GGTTTTTCCC | 1.962 | 0.902 | 0.758 | 0.702 | TGAGCTTCCC | 0.000 | 0.809 | 0.726 | 0.657 |
| GGGGATCCCC | 1.954 | 0.898 | 1.000 | 0.991 | TGGATTGCCC | 0.000 | 0.840 | 0.825 | 0.687 |
| GGGCTGTCCC | 1.949 | 0.809 | 0.875 | 0.775 | GGTGTCGCCC | 0.000 | 0.800 | 0.767 | 0.690 |
| GGGTATTTCC | 1.949 | 0.894 | 0.888 | 0.834 | GGCATCTCCC | 0.000 | 0.789 | 0.755 | 0.638 |
| GGGGAATTCC | 1.929 | 0.878 | 0.904 | 0.848 | GGTACCGCCC | 0.000 | 0.791 | 0.750 | 0.613 |
| GGTAGTTCCC | 1.924 | 0.970 | 0.765 | 0.708 | GGATCGTCCC | 0.000 | 0.749 | 0.750 | 0.642 |
| GGGGGTTTTT | 1.924 | 0.779 | 0.656 | 0.602 | GAGGCTTTCC | 0.000 | 0.832 | 0.833 | 0.713 |
| GGGTTCTCCC | 1.919 | 0.819 | 0.808 | 0.760 | GGGGATTACT | 0.000 | 0.771 | 0.799 | 0.805 |
| GGGTTTACCC | 1.916 | 0.819 | 0.852 | 0.806 | GATGGTTTCC | 0.000 | 0.820 | 0.638 | 0.575 |
| GGGGAGTTCC | 1.914 | 0.873 | 0.971 | 0.863 | GTGAGTGTAC | 0.000 | 0.752 | 0.612 | 0.472 |
| GGGTTTTTCC | 1.913 | 0.898 | 0.866 | 0.757 | GGTGTCGTCC | 0.000 | 0.774 | 0.748 | 0.613 |
| GGGTTTTACC | 1.912 | 0.811 | 0.793 | 0.746 | CGTTCTTCCC | 0.000 | 0.780 | 0.633 | 0.569 |
| GGGATTCCCG | 1.911 | 0.822 | 0.931 | 0.737 | AGTTCTTCCC | 0.000 | 0.784 | 0.633 | 0.569 |
| GGGATCTCCC | 1.900 | 0.895 | 0.881 | 0.771 | GGGGTTGACC | 0.000 | 0.813 | 0.878 | 0.809 |
| GGGGATCTCC | 1.900 | 0.872 | 0.980 | 0.914 | AGTGCATCCC | 0.000 | 0.767 | 0.649 | 0.582 |
| GTAGTTTCCC | 1.894 | 0.819 | 0.724 | 0.657 | GGGAATTGGC | 0.000 | 0.768 | 0.762 | 0.701 |
| GGGGAGTCCC | 1.889 | 0.898 | 0.990 | 0.940 | GGGATTAACC | 0.000 | 0.783 | 0.832 | 0.729 |
| GGGGATTTTA | 1.878 | 0.798 | 0.746 | 0.683 | AGAAGTGCCC | 0.000 | 0.758 | 0.631 | 0.549 |
| GGGGGATTCC | 1.877 | 0.872 | 0.815 | 0.766 | GGGAGTTTGC | 0.000 | 0.871 | 0.746 | 0.630 |
| GGAGTTTCCC | 1.871 | 0.922 | 0.851 | 0.790 | GGGGGTGTCA | 0.000 | 0.802 | 0.776 | 0.709 |
| GGGGATGTCC | 1.869 | 0.896 | 0.974 | 0.897 | GAGAATTTCG | 0.000 | 0.767 | 0.808 | 0.612 |
| GGTGTTCCCC | 1.867 | 0.880 | 0.850 | 0.782 | TAGAGTTCCC | 0.000 | 0.782 | 0.638 | 0.575 |
| GGGGTTTTAC | 1.858 | 0.918 | 0.831 | 0.713 | CTGGGTTCCC | 0.000 | 0.774 | 0.657 | 0.652 |
| GGGGCTTTTC | 1.856 | 0.872 | 0.833 | 0.713 | GATGTTTCTC | 0.000 | 0.781 | 0.597 | 0.524 |
| GGGGCTTTTA | 1.854 | 0.783 | 0.725 | 0.607 | CGTGATTCTC | 0.000 | 0.787 | 0.620 | 0.601 |
| GGAGTTCCCC | 1.842 | 0.833 | 0.850 | 0.782 | TGGGGTGCTC | 0.000 | 0.748 | 0.650 | 0.627 |
| GGGGTTTTTC | 1.838 | 0.891 | 0.831 | 0.713 | GGGAGTTTAG | 0.000 | 0.830 | 0.718 | 0.530 |
| GGGAGTTTCC | 1.837 | 0.966 | 0.872 | 0.763 | GCGGTTTACC | 0.000 | 0.750 | 0.758 | 0.702 |
| AGGGGTTTCC | 1.834 | 0.855 | 0.765 | 0.708 | AGTGCTTTAC | 0.000 | 0.778 | 0.580 | 0.447 |
| GGTAGTTTCC | 1.831 | 0.945 | 0.746 | 0.630 | GGTGATTAAC | 0.000 | 0.806 | 0.654 | 0.646 |
| GGGGGATTTC | 1.830 | 0.799 | 0.688 | 0.633 | GGTAACTTTC | 0.000 | 0.771 | 0.631 | 0.504 |
| GGGATTTACC | 1.826 | 0.887 | 0.866 | 0.757 | TGGACTTTAC | 0.000 | 0.814 | 0.687 | 0.502 |
| GTATTTTCCC | 1.824 | 0.753 | 0.631 | 0.569 | GGGAGTGTCG | 0.000 | 0.812 | 0.838 | 0.638 |
| CGGGATTTCC | 1.823 | 0.856 | 0.854 | 0.790 | CGTGTTACCC | 0.000 | 0.760 | 0.690 | 0.629 |
| TGGGGATTCC | 1.820 | 0.777 | 0.688 | 0.633 | GATAATTCCC | 0.000 | 0.861 | 0.727 | 0.657 |
| GTGGATTCCC | 1.820 | 0.884 | 0.873 | 0.867 | GGTACCTCCG | 0.000 | 0.767 | 0.729 | 0.538 |
| GGGGCTTTTT | 1.815 | 0.769 | 0.725 | 0.607 | GGGCGTCTCC | 0.000 | 0.774 | 0.799 | 0.744 |
| GGGGACTCCC | 1.810 | 0.882 | 0.923 | 0.925 | GTGCGTTCCC | 0.000 | 0.786 | 0.691 | 0.697 |
| GGGCATTCCC | 1.799 | 0.894 | 0.908 | 0.912 | GGTGGATTCA | 0.000 | 0.762 | 0.579 | 0.527 |
| CGGGGATTCC | 1.796 | 0.768 | 0.688 | 0.633 | TTTATTTCCC | 0.000 | 0.781 | 0.578 | 0.447 |
| GGTAATTCCC | 1.782 | 0.975 | 0.854 | 0.790 | CGTATTTTCC | 0.000 | 0.849 | 0.686 | 0.502 |
| GGGGATTTTT | 1.781 | 0.784 | 0.746 | 0.683 | GGTACTCTGC | 0.000 | 0.751 | 0.687 | 0.494 |
| GGGGTTTTTA | 1.779 | 0.802 | 0.723 | 0.607 | GGTGGTCCAC | 0.000 | 0.825 | 0.657 | 0.644 |
| GGGACTTTTC | 1.768 | 0.882 | 0.814 | 0.635 | GGGATTGTAA | 0.000 | 0.774 | 0.697 | 0.504 |
| GTTATTCCCC | 1.763 | 0.787 | 0.704 | 0.571 | GGAGGGTCTC | 0.000 | 0.752 | 0.647 | 0.592 |
| GGGCTTTACC | 1.760 | 0.785 | 0.793 | 0.746 | GGATCTTCTC | 0.000 | 0.764 | 0.633 | 0.569 |
| GGGATTTCCG | 1.759 | 0.911 | 0.931 | 0.746 | CGGGATTTCG | 0.000 | 0.767 | 0.827 | 0.690 |
| TGGGATTCCC | 1.746 | 0.892 | 0.873 | 0.867 | GGTGGTCCCA | 0.000 | 0.782 | 0.675 | 0.670 |
| GGGGATTTTG | 1.738 | 0.798 | 0.827 | 0.690 | GGTGAATTCA | 0.000 | 0.768 | 0.669 | 0.609 |
| TGGGATTTCC | 1.736 | 0.866 | 0.854 | 0.790 | CGTAGTTCCG | 0.000 | 0.776 | 0.611 | 0.475 |
| GGTATTTTCC | 1.735 | 0.953 | 0.812 | 0.635 | GGGAATCTCT | 0.000 | 0.778 | 0.853 | 0.731 |
| GGGATTTCCT | 1.717 | 0.897 | 0.850 | 0.740 | TGTGCTGCCC | 0.000 | 0.791 | 0.719 | 0.632 |
| AGGGGTTTTC | 1.714 | 0.782 | 0.638 | 0.575 | GGGCTTGTCC | 0.000 | 0.807 | 0.859 | 0.732 |
| GGGGACTTCC | 1.712 | 0.856 | 0.904 | 0.848 | AGTGTTTTAC | 0.000 | 0.796 | 0.578 | 0.447 |
| AGGGGATTCC | 1.712 | 0.772 | 0.688 | 0.633 | GGGGTCTTCT | 0.000 | 0.756 | 0.773 | 0.665 |
| GGGCATCCCC | 1.708 | 0.805 | 0.907 | 0.903 | AGGCTTTTCC | 0.000 | 0.771 | 0.739 | 0.624 |
| GGGGTTCTCC | 1.708 | 0.875 | 0.958 | 0.837 | TGGATTTCAC | 0.000 | 0.858 | 0.705 | 0.580 |
| GGGGTTTTTT | 1.706 | 0.787 | 0.723 | 0.607 | CGGGTGTCAC | 0.000 | 0.751 | 0.714 | 0.597 |
| GGGGCGTCCC | 1.703 | 0.884 | 0.970 | 0.863 | GGGACGCTCC | 0.000 | 0.779 | 0.931 | 0.700 |
| GTGCATTCCC | 1.699 | 0.791 | 0.781 | 0.779 | GGGATGTCTA | 0.000 | 0.749 | 0.713 | 0.547 |
| GGGACGTCCC | 1.699 | 0.894 | 0.951 | 0.786 | GTAGTTTCAC | 0.000 | 0.773 | 0.597 | 0.524 |
| GGTGCATTCC | 1.695 | 0.842 | 0.757 | 0.638 | GGTATTATTC | 0.000 | 0.775 | 0.652 | 0.474 |
| GGGGGTTTAC | 1.692 | 0.909 | 0.765 | 0.708 | GGGACTCCTC | 0.000 | 0.819 | 0.833 | 0.704 |
| AGGGGGTTCC | 1.690 | 0.767 | 0.755 | 0.648 | TGAGATTCCC | 0.000 | 0.824 | 0.746 | 0.734 |
| GGGGGATCCC | 1.690 | 0.898 | 0.834 | 0.843 | TATGGTTCCC | 0.000 | 0.751 | 0.530 | 0.519 |
| GGGATTCTCC | 1.690 | 0.885 | 0.939 | 0.760 | GGGGTTCCGC | 0.000 | 0.806 | 0.850 | 0.782 |
| GGGGGTGTCC | 1.689 | 0.891 | 0.885 | 0.815 | GGGGGTTACA | 0.000 | 0.780 | 0.710 | 0.723 |
| GGTGGATTCC | 1.683 | 0.851 | 0.688 | 0.633 | CGGAATTTAC | 0.000 | 0.820 | 0.708 | 0.579 |
| GGTTATTCCC | 1.683 | 0.899 | 0.781 | 0.779 | GGGCTAGCCC | 0.000 | 0.750 | 0.802 | 0.734 |
| GGGGCATCCC | 1.681 | 0.889 | 0.903 | 0.848 | GGAGTATTCC | 0.000 | 0.813 | 0.755 | 0.638 |
| GGGCTTTTCC | 1.677 | 0.872 | 0.866 | 0.757 | GGGTTATCTC | 0.000 | 0.766 | 0.681 | 0.627 |
| GGGTGTTCCC | 1.677 | 0.915 | 0.818 | 0.830 | TGGATCTCCC | 0.000 | 0.800 | 0.755 | 0.638 |
| GGGATTCCCT | 1.674 | 0.808 | 0.849 | 0.731 | GGCCATTCCC | 0.000 | 0.788 | 0.781 | 0.779 |
| GGGTTGTCCC | 1.673 | 0.835 | 0.875 | 0.775 | GGTTCTTGCC | 0.000 | 0.751 | 0.668 | 0.613 |
| GTAATTTCCC | 1.672 | 0.829 | 0.705 | 0.580 | GATGTTTCCC | 0.000 | 0.855 | 0.724 | 0.657 |
| GGGGATTTCT | 1.670 | 0.858 | 0.872 | 0.816 | GATTCTTCCC | 0.000 | 0.770 | 0.633 | 0.569 |
| GGGGCTGTCC | 1.665 | 0.882 | 0.954 | 0.820 | GGGATGTCGC | 0.000 | 0.817 | 0.822 | 0.653 |
| GGGTCTTCCC | 1.660 | 0.905 | 0.887 | 0.835 | TGTATTTCAC | 0.000 | 0.837 | 0.578 | 0.447 |
| GGGGGTTTTG | 1.660 | 0.793 | 0.737 | 0.608 | GTAGCTTCCC | 0.000 | 0.801 | 0.726 | 0.657 |
| GGTAATTTCC | 1.658 | 0.950 | 0.835 | 0.712 | TGTGGTCTCC | 0.000 | 0.751 | 0.638 | 0.566 |
| GGGAATTTCC | 1.658 | 0.971 | 0.962 | 0.845 | TGTATTTCAG | 0.000 | 0.748 | 0.551 | 0.347 |
| GGGGTTTTTG | 1.657 | 0.801 | 0.804 | 0.613 | CTGAGTTTCC | 0.000 | 0.758 | 0.619 | 0.497 |
| GGGGACTTTC | 1.656 | 0.783 | 0.777 | 0.715 | GGGGATTAAC | 0.000 | 0.828 | 0.781 | 0.779 |
| GGGGATTACC | 1.653 | 0.874 | 0.908 | 0.912 | CGTGGGTCCC | 0.000 | 0.768 | 0.647 | 0.592 |
| GGGGTATTCC | 1.651 | 0.881 | 0.881 | 0.771 | GCAATTTCCC | 0.000 | 0.804 | 0.705 | 0.580 |
| GGGACTTTTT | 1.648 | 0.779 | 0.706 | 0.529 | GGGCGCTTCC | 0.000 | 0.759 | 0.722 | 0.677 |
| AGGGTTTTCC | 1.645 | 0.864 | 0.831 | 0.713 | AGTAATTCCC | 0.000 | 0.875 | 0.727 | 0.657 |
| GGGGCATTCC | 1.643 | 0.863 | 0.883 | 0.771 | TCGATTTCCC | 0.000 | 0.777 | 0.705 | 0.580 |
| GGGTATCCCC | 1.638 | 0.831 | 0.907 | 0.903 | GTGAGGTCCC | 0.000 | 0.800 | 0.755 | 0.648 |
| GGTTTTACCC | 1.633 | 0.798 | 0.725 | 0.673 | GGTTCTGCCC | 0.000 | 0.820 | 0.754 | 0.676 |
| GGTCTTCCCC | 1.627 | 0.788 | 0.758 | 0.693 | GGGCTTCCAC | 0.000 | 0.762 | 0.758 | 0.693 |
| GGGGTCTCCC | 1.620 | 0.885 | 0.901 | 0.848 | ATTATTTCCC | 0.000 | 0.776 | 0.578 | 0.447 |
| GTGGTTTACC | 1.620 | 0.775 | 0.758 | 0.702 | GGGGATACAA | 0.000 | 0.747 | 0.731 | 0.733 |
| CGGGGTTTCC | 1.619 | 0.851 | 0.765 | 0.708 | GGGAATCGCC | 0.000 | 0.774 | 0.888 | 0.826 |
| GGGGGAGTCC | 1.618 | 0.808 | 0.808 | 0.740 | TGGAGGTTCC | 0.000 | 0.782 | 0.736 | 0.570 |
| GGGGTTACCC | 1.613 | 0.886 | 0.944 | 0.895 | AGGGGTACCC | 0.000 | 0.777 | 0.750 | 0.757 |
| GGGATTTTAC | 1.609 | 0.928 | 0.812 | 0.635 | GGGGGGGCAC | 0.000 | 0.782 | 0.768 | 0.700 |
| GGAATTTCCC | 1.607 | 0.932 | 0.831 | 0.713 | GGTCGTGCCC | 0.000 | 0.803 | 0.685 | 0.671 |
| GGGGCTTTTG | 1.604 | 0.783 | 0.806 | 0.613 | GGAAGAGCCC | 0.000 | 0.776 | 0.681 | 0.607 |
| GGGTTTTCCA | 1.602 | 0.834 | 0.776 | 0.729 | GGGGGTGCAG | 0.000 | 0.781 | 0.750 | 0.660 |
| GGGGTATCCC | 1.602 | 0.907 | 0.901 | 0.848 | GGGCCATTCC | 0.000 | 0.770 | 0.791 | 0.683 |
| GGGTTATCCC | 1.602 | 0.840 | 0.808 | 0.760 | GGATCTTCCG | 0.000 | 0.748 | 0.733 | 0.602 |
| GGGTTTTCCG | 1.601 | 0.834 | 0.858 | 0.735 | GGGATTCGCC | 0.000 | 0.778 | 0.865 | 0.749 |
| GTTATTTCCC | 1.599 | 0.876 | 0.705 | 0.580 | TGTAAGTCCC | 0.000 | 0.792 | 0.718 | 0.597 |
| GGTTTTCCAC | 1.591 | 0.767 | 0.631 | 0.560 | CGGATTTGCC | 0.000 | 0.762 | 0.739 | 0.624 |
| GGGTGTCCCC | 1.591 | 0.826 | 0.818 | 0.821 | GTAACTTTCC | 0.000 | 0.785 | 0.687 | 0.502 |
| GGTGGATCCC | 1.585 | 0.877 | 0.707 | 0.710 | AGTGGATTCC | 0.000 | 0.751 | 0.561 | 0.500 |
| GGGGAGCCCC | 1.580 | 0.810 | 0.990 | 0.932 | GGATATTCCG | 0.000 | 0.763 | 0.754 | 0.679 |
| GGGATTGCCC | 1.574 | 0.935 | 0.952 | 0.820 | GGGGGTGCGC | 0.000 | 0.822 | 0.777 | 0.760 |
| GGGACTGTCC | 1.574 | 0.891 | 0.934 | 0.743 | CGTGCGTCCC | 0.000 | 0.758 | 0.716 | 0.597 |
| GGGTACTTCC | 1.574 | 0.790 | 0.812 | 0.759 | GGTAGTTTCA | 0.000 | 0.856 | 0.637 | 0.524 |
| GGTCTTTCCC | 1.569 | 0.877 | 0.758 | 0.702 | GGCATTGTAC | 0.000 | 0.756 | 0.679 | 0.477 |
| GGGGGTTCAC | 1.568 | 0.935 | 0.784 | 0.785 | TGTAGATTCC | 0.000 | 0.766 | 0.542 | 0.423 |
| GGGGGGTTTC | 1.567 | 0.794 | 0.755 | 0.648 | GCGGATTTTC | 0.000 | 0.759 | 0.727 | 0.657 |
| GGGGTTCCCG | 1.562 | 0.812 | 0.950 | 0.815 | GGGGCTGGCC | 0.000 | 0.774 | 0.880 | 0.809 |
| GGGGTTCACC | 1.562 | 0.789 | 0.885 | 0.826 | CGTTATTTCC | 0.000 | 0.769 | 0.635 | 0.568 |
| TGGACTTTCC | 1.560 | 0.861 | 0.814 | 0.635 | GGGGCGTCCG | 0.000 | 0.794 | 0.942 | 0.763 |
| GGTGCTTTTT | 1.560 | 0.748 | 0.598 | 0.474 | GGGGGATCCG | 0.000 | 0.809 | 0.806 | 0.743 |
| CGGGGTTTTC | 1.558 | 0.777 | 0.638 | 0.575 | TGTGGGTCCC | 0.000 | 0.777 | 0.647 | 0.592 |
| GTGGATCCCC | 1.556 | 0.795 | 0.873 | 0.859 | TGCATTTCCC | 0.000 | 0.798 | 0.705 | 0.580 |
| GGGGGTTACC | 1.556 | 0.869 | 0.818 | 0.830 | CGGGCTTCGC | 0.000 | 0.773 | 0.726 | 0.657 |
| CGGGATTCCC | 1.550 | 0.882 | 0.873 | 0.867 | GGTAGCTCCG | 0.000 | 0.776 | 0.661 | 0.533 |
| GGATTCTCCC | 1.550 | 0.751 | 0.681 | 0.627 | GGTTACTTCC | 0.000 | 0.768 | 0.685 | 0.626 |
| GTGGTTACCC | 1.550 | 0.783 | 0.817 | 0.762 | GGGAATGTCA | 0.000 | 0.817 | 0.847 | 0.714 |
| GGGTAATTCC | 1.550 | 0.811 | 0.812 | 0.759 | GGTTGTTTTC | -0.004 | 0.794 | 0.545 | 0.486 |
| GGGGATGCAC | 1.550 | 0.876 | 0.867 | 0.842 | GGGGTGTTGC | -0.018 | 0.781 | 0.822 | 0.653 |
| GGGGTTTTAT | 1.548 | 0.814 | 0.723 | 0.607 | TTGGCTTTCC | -0.022 | 0.748 | 0.707 | 0.580 |
| GGGGAATTTC | 1.548 | 0.804 | 0.777 | 0.715 | GGGCGTTTTC | -0.022 | 0.790 | 0.672 | 0.619 |
| GGGACTTTTA | 1.544 | 0.793 | 0.706 | 0.529 | TGTGGTGTCC | -0.022 | 0.775 | 0.631 | 0.549 |
| GGGTGTTTCC | 1.540 | 0.889 | 0.799 | 0.752 | TGTACTTTTC | -0.027 | 0.766 | 0.561 | 0.369 |
| GGGTTTTCCT | 1.537 | 0.820 | 0.776 | 0.729 | GGGGTCTTCG | -0.032 | 0.770 | 0.854 | 0.671 |
| GGGGCTTTAC | 1.535 | 0.900 | 0.833 | 0.713 | GGGGGTGCTG | -0.032 | 0.754 | 0.750 | 0.660 |
| GGTTTTTACC | 1.528 | 0.790 | 0.666 | 0.613 | GGTGGTTCTG | -0.032 | 0.797 | 0.630 | 0.552 |
| GGTGGAGTCC | 1.525 | 0.787 | 0.681 | 0.607 | GGGTTTGTAC | -0.036 | 0.787 | 0.732 | 0.599 |
| GGGGTTTTCT | 1.524 | 0.861 | 0.850 | 0.740 | TGTGTGTCCC | -0.036 | 0.786 | 0.714 | 0.597 |
| CGGGTTTTCC | 1.523 | 0.860 | 0.831 | 0.713 | GGTGCTTCTC | -0.036 | 0.877 | 0.726 | 0.657 |
| GGGTTTTTAC | 1.522 | 0.851 | 0.739 | 0.624 | CGTGTTGTCC | -0.046 | 0.774 | 0.698 | 0.554 |
| GGTTTCTCCC | 1.522 | 0.798 | 0.681 | 0.627 | GGTGCCTTTC | -0.046 | 0.747 | 0.630 | 0.505 |
| GGGGGCTTCC | 1.522 | 0.851 | 0.815 | 0.766 | GGGGTTTGCT | -0.046 | 0.753 | 0.776 | 0.729 |
| GGGGATTCCT | 1.521 | 0.883 | 0.892 | 0.894 | GGCGCTTTTC | -0.046 | 0.766 | 0.707 | 0.580 |
| GTGGAGTCCC | 1.519 | 0.796 | 0.864 | 0.807 | TGTGATTTGC | -0.046 | 0.750 | 0.601 | 0.524 |
| GGTGGTTTTC | 1.515 | 0.861 | 0.638 | 0.575 | TGTGATTCTC | -0.051 | 0.797 | 0.620 | 0.601 |
| GGATTGTCCC | 1.512 | 0.767 | 0.749 | 0.642 | GGTGGTTTAG | -0.051 | 0.799 | 0.611 | 0.475 |
| GGGATTCACC | 1.512 | 0.798 | 0.865 | 0.749 | GGTTATTCCT | -0.051 | 0.796 | 0.672 | 0.672 |
| GGGATTTTTC | 1.508 | 0.900 | 0.812 | 0.635 | GGGGCTTTAG | -0.056 | 0.810 | 0.806 | 0.613 |
| GGCTTTTCCC | 1.507 | 0.817 | 0.758 | 0.702 | GGGGGTGTCT | -0.056 | 0.788 | 0.776 | 0.709 |
| GGACTTTTCC | 1.505 | 0.804 | 0.739 | 0.624 | TTTGTTTCCC | -0.056 | 0.771 | 0.597 | 0.524 |
| GGGGCTCTCC | 1.505 | 0.857 | 0.960 | 0.837 | GGGGTTCTGC | -0.056 | 0.781 | 0.831 | 0.704 |
| TGGGGTTTCC | 1.505 | 0.861 | 0.765 | 0.708 | GGTCTTTTTC | -0.060 | 0.777 | 0.612 | 0.491 |
| GGGACTTTTG | 1.502 | 0.793 | 0.787 | 0.536 | GTGGTGTTCC | -0.060 | 0.773 | 0.822 | 0.653 |
| GGGCCTTCCC | 1.498 | 0.879 | 0.887 | 0.835 | GGTAGTTTCG | -0.066 | 0.855 | 0.718 | 0.530 |
| GGCATTCCCC | 1.498 | 0.804 | 0.831 | 0.704 | TATGTTTCCC | -0.066 | 0.760 | 0.597 | 0.524 |
| GGGGATTCAC | 1.498 | 0.940 | 0.873 | 0.867 | GATGTTTTTC | -0.066 | 0.755 | 0.578 | 0.447 |
| GGGGCGCCCC | 1.491 | 0.795 | 0.969 | 0.855 | GGTTCTTCCT | -0.066 | 0.781 | 0.652 | 0.596 |
| GGGGGCTTTC | 1.489 | 0.777 | 0.688 | 0.633 | GGGGTTGTCA | -0.066 | 0.811 | 0.843 | 0.714 |
| GGGGTTTCCT | 1.488 | 0.887 | 0.869 | 0.817 | GGTTCTTTTC | -0.066 | 0.785 | 0.614 | 0.491 |
| GGGGGCGTCC | 1.484 | 0.786 | 0.808 | 0.740 | GGGGTTATCG | -0.066 | 0.771 | 0.898 | 0.718 |
| GGGCGTCCCC | 1.484 | 0.800 | 0.818 | 0.821 | TGGATTTTGC | -0.071 | 0.784 | 0.686 | 0.502 |
| GGTGGTTTTT | 1.484 | 0.757 | 0.529 | 0.469 | GGTTCTCTCC | -0.081 | 0.769 | 0.741 | 0.616 |
| GGGGTTTCAC | 1.481 | 0.944 | 0.851 | 0.790 | TGTGATGTCC | -0.081 | 0.780 | 0.721 | 0.631 |
| GGGGATTCTC | 1.477 | 0.913 | 0.873 | 0.867 | GGGGGGTCCT | -0.081 | 0.790 | 0.792 | 0.752 |
| GGGCTCTCCC | 1.477 | 0.793 | 0.808 | 0.760 | GGTTGATTCC | -0.081 | 0.785 | 0.595 | 0.544 |
| CGGGGTTCCC | 1.477 | 0.877 | 0.784 | 0.785 | GGGAGTGCTT | -0.081 | 0.750 | 0.650 | 0.576 |
| GGAGATTCCC | 1.477 | 0.919 | 0.873 | 0.867 | GGTGCGTTCC | -0.081 | 0.837 | 0.824 | 0.653 |
| GGGATTTTGC | 1.474 | 0.879 | 0.812 | 0.635 | CTTGGTTCCC | -0.081 | 0.753 | 0.530 | 0.519 |
| GGGGGTGCCC | 1.470 | 0.917 | 0.904 | 0.893 | GGTACTGTTC | -0.081 | 0.797 | 0.681 | 0.477 |
| GGGATTACCC | 1.470 | 0.896 | 0.925 | 0.817 | GCGGTGTTCC | -0.081 | 0.748 | 0.822 | 0.653 |
| AGGTGTTTCC | 1.465 | 0.789 | 0.672 | 0.619 | TGGTGTGCCC | -0.081 | 0.755 | 0.685 | 0.671 |
| GGGATTTTCG | 1.465 | 0.885 | 0.912 | 0.669 | AGTATTTTTC | -0.081 | 0.779 | 0.559 | 0.369 |
| GGGGTGCCCC | 1.462 | 0.813 | 0.967 | 0.855 | GGGGCGTTGC | -0.081 | 0.763 | 0.824 | 0.653 |
| GGTTATCCCC | 1.462 | 0.810 | 0.780 | 0.770 | GGTGCTATTC | -0.081 | 0.747 | 0.673 | 0.552 |
| GGGGTTTTCA | 1.462 | 0.875 | 0.850 | 0.740 | GGGCTTTGCC | -0.081 | 0.764 | 0.793 | 0.746 |
| GGCATTTCCC | 1.462 | 0.893 | 0.831 | 0.713 | GGTGGTTATC | -0.081 | 0.774 | 0.565 | 0.564 |
| GGGTAGTCCC | 1.462 | 0.832 | 0.898 | 0.852 | GGGGGTTTGA | -0.086 | 0.772 | 0.656 | 0.602 |
| GGGGGGTTCC | 1.461 | 0.867 | 0.882 | 0.781 | GGTGTTTCTG | -0.092 | 0.806 | 0.697 | 0.557 |
| GGGGTTTCCA | 1.459 | 0.901 | 0.869 | 0.817 | TGCGTTTTCC | -0.092 | 0.763 | 0.705 | 0.580 |
| GGGATTTTAT | 1.456 | 0.824 | 0.704 | 0.529 | GGTGTTTTGG | -0.092 | 0.759 | 0.677 | 0.480 |
| CGGGAATTCC | 1.455 | 0.773 | 0.777 | 0.715 | CGTCTTTCCC | -0.097 | 0.772 | 0.631 | 0.569 |
| GGGGACGTCC | 1.455 | 0.792 | 0.897 | 0.822 | GTGAATTTCT | -0.097 | 0.765 | 0.726 | 0.606 |
| GGTATCTCCC | 1.455 | 0.874 | 0.755 | 0.638 | GTGATTTCGC | -0.097 | 0.802 | 0.705 | 0.580 |
| GGGACTGCCC | 1.455 | 0.917 | 0.954 | 0.820 | TGTGAGTTCC | -0.097 | 0.756 | 0.718 | 0.597 |
| GGGATGTACC | 1.455 | 0.799 | 0.856 | 0.698 | TGCGGTTTCC | -0.097 | 0.754 | 0.638 | 0.575 |
| GGGTATCTCC | 1.455 | 0.805 | 0.888 | 0.826 | TGTCATTTCC | -0.097 | 0.752 | 0.635 | 0.568 |
| GGTACTTTTT | 1.451 | 0.758 | 0.579 | 0.396 | GTGTGTTTCC | -0.097 | 0.786 | 0.672 | 0.619 |
| GTTGTTCCCC | 1.445 | 0.777 | 0.723 | 0.649 | GTGACTTTGC | -0.097 | 0.758 | 0.687 | 0.502 |
| CGGGGGTTCC | 1.443 | 0.763 | 0.755 | 0.648 | TGGGTTGCTC | -0.097 | 0.757 | 0.717 | 0.632 |
| GGGCATTTCC | 1.439 | 0.869 | 0.888 | 0.834 | GGTTGTTCCA | -0.097 | 0.805 | 0.583 | 0.591 |
| GGGGGTCCCC | 1.439 | 0.893 | 0.910 | 0.910 | GTGGATTCTC | -0.097 | 0.810 | 0.746 | 0.734 |
| GTGATGTCCC | 1.439 | 0.809 | 0.822 | 0.653 | TGGATTTCCT | -0.097 | 0.801 | 0.723 | 0.607 |
| GTGGTTTTCC | 1.438 | 0.861 | 0.831 | 0.713 | GTGGATTCCT | -0.097 | 0.780 | 0.765 | 0.761 |
| GGTTATTTCC | 1.438 | 0.873 | 0.762 | 0.701 | TGCGTTTCCC | -0.097 | 0.788 | 0.724 | 0.657 |
| GGGATTTCCA | 1.431 | 0.911 | 0.850 | 0.740 | GGTAGCTTTC | -0.097 | 0.766 | 0.542 | 0.423 |
| TGGGAATTCC | 1.431 | 0.783 | 0.777 | 0.715 | GGGTTTGCCT | -0.097 | 0.755 | 0.770 | 0.703 |
| GGGAGATTCC | 1.431 | 0.882 | 0.795 | 0.688 | GGGAGTGTTC | -0.097 | 0.827 | 0.739 | 0.605 |
| GTGGTGTCCC | 1.431 | 0.799 | 0.841 | 0.730 | GGGCGTTCCT | -0.097 | 0.786 | 0.710 | 0.723 |
| GGGGATGTAC | 1.431 | 0.850 | 0.848 | 0.764 | GGGGATTTGA | -0.097 | 0.777 | 0.746 | 0.683 |
| GGTGCTTTTC | 1.431 | 0.851 | 0.707 | 0.580 | GGCGGTTTCC | -0.097 | 0.849 | 0.765 | 0.708 |
| GGGATTTCTC | 1.427 | 0.926 | 0.831 | 0.713 | TGTAGTGTCC | -0.097 | 0.785 | 0.612 | 0.472 |
| GGGGGTTTCT | 1.425 | 0.852 | 0.783 | 0.735 | TGTAGTTCGC | -0.097 | 0.780 | 0.511 | 0.442 |
| GTGAATTCCC | 1.423 | 0.894 | 0.854 | 0.790 | GATGTTGTCC | -0.097 | 0.764 | 0.698 | 0.554 |
| GGGGTTGCCC | 1.419 | 0.926 | 0.971 | 0.898 | GGTACTTTAT | -0.097 | 0.785 | 0.579 | 0.396 |
| GGTTTTTTCC | 1.417 | 0.877 | 0.739 | 0.624 | TGTAGTTTGC | -0.102 | 0.755 | 0.492 | 0.364 |
| GGGATATCCC | 1.415 | 0.917 | 0.881 | 0.771 | GGTCTTTCCT | -0.102 | 0.773 | 0.650 | 0.596 |
| GGGGGTCTCC | 1.415 | 0.867 | 0.891 | 0.832 | GGGGCTTGTC | -0.102 | 0.765 | 0.760 | 0.702 |
| GGGATTCCCA | 1.415 | 0.822 | 0.849 | 0.731 | TGTGGTTTTC | -0.102 | 0.766 | 0.511 | 0.442 |
| GGGGTTTCCG | 1.415 | 0.901 | 0.950 | 0.823 | TGTGTTCTCC | -0.102 | 0.759 | 0.704 | 0.571 |
| GGTTTTTCCG | 1.414 | 0.813 | 0.731 | 0.602 | TGGATTTCTC | -0.108 | 0.831 | 0.705 | 0.580 |
| GGTTTTTCCA | 1.410 | 0.813 | 0.650 | 0.596 | TGAGTTTTCC | -0.108 | 0.801 | 0.705 | 0.580 |
| CGGTATTTCC | 1.409 | 0.790 | 0.762 | 0.701 | GGTATTCTCT | -0.108 | 0.761 | 0.703 | 0.521 |
| GGGGGGTCCC | 1.407 | 0.893 | 0.901 | 0.858 | GGTATTTCTG | -0.108 | 0.816 | 0.677 | 0.480 |
| GGGCTTTCCG | 1.405 | 0.808 | 0.858 | 0.735 | TGTGTGTTCC | -0.114 | 0.760 | 0.695 | 0.520 |
| GGGTCTTTCC | 1.404 | 0.879 | 0.868 | 0.757 | GGTTCTTCCG | -0.114 | 0.795 | 0.733 | 0.602 |
| GGGGTTTTCG | 1.401 | 0.875 | 0.931 | 0.746 | GTTATTTCCT | -0.119 | 0.773 | 0.596 | 0.474 |
| CGGTGTTTCC | 1.400 | 0.785 | 0.672 | 0.619 | CGTGGTTTTC | -0.119 | 0.756 | 0.511 | 0.442 |
| TGGGGTTTTC | 1.400 | 0.787 | 0.638 | 0.575 | GATGGTTTTC | -0.119 | 0.747 | 0.511 | 0.442 |
| GGTTCTCCCC | 1.398 | 0.795 | 0.760 | 0.693 | TGGATTGCTC | -0.125 | 0.767 | 0.698 | 0.554 |
| GGTGAATTCC | 1.398 | 0.857 | 0.777 | 0.715 | GGTTCTTCGC | -0.125 | 0.789 | 0.633 | 0.569 |
| GGTGGTTACC | 1.398 | 0.848 | 0.691 | 0.697 | GTTAATTTGC | -0.125 | 0.752 | 0.581 | 0.446 |
| GGGATTGTCC | 1.398 | 0.910 | 0.932 | 0.743 | GGGGAGTTGC | -0.125 | 0.778 | 0.844 | 0.730 |
| GGTACTTTTC | 1.397 | 0.861 | 0.687 | 0.502 | GGCGTTTTAC | -0.125 | 0.811 | 0.705 | 0.580 |
| GGTGGTTTAC | 1.394 | 0.888 | 0.638 | 0.575 | GCTCTTTCCC | -0.125 | 0.749 | 0.631 | 0.569 |
| GGGTGATTCC | 1.394 | 0.806 | 0.722 | 0.677 | TTTGATTCCC | -0.125 | 0.768 | 0.620 | 0.601 |
| GGGGATTGCC | 1.392 | 0.853 | 0.908 | 0.912 | TGTGCGTCCC | -0.125 | 0.767 | 0.716 | 0.597 |
| CGGGAGTTCC | 1.389 | 0.768 | 0.844 | 0.730 | CGGGATTTGC | -0.125 | 0.762 | 0.727 | 0.657 |
| GGTGATCTCC | 1.389 | 0.851 | 0.854 | 0.781 | GGGGCATTCT | -0.125 | 0.759 | 0.775 | 0.665 |
| GGTATGTCCC | 1.389 | 0.891 | 0.822 | 0.653 | GGGGATGTGC | -0.125 | 0.802 | 0.848 | 0.764 |
| AGGGTTTCCC | 1.389 | 0.890 | 0.851 | 0.790 | CGTGTGTCCC | -0.125 | 0.776 | 0.714 | 0.597 |
| GTGATCTCCC | 1.389 | 0.792 | 0.755 | 0.638 | GGTATCTTTC | -0.125 | 0.775 | 0.609 | 0.428 |
| GGGCTTTTAC | 1.387 | 0.825 | 0.739 | 0.624 | GGTTATTTCT | -0.125 | 0.770 | 0.653 | 0.595 |
| GGGTTTCTCC | 1.387 | 0.809 | 0.865 | 0.749 | AGTGTGTTCC | -0.125 | 0.754 | 0.695 | 0.520 |
| GGGATTTTTG | 1.386 | 0.811 | 0.785 | 0.536 | TGGATTTTCA | -0.125 | 0.790 | 0.704 | 0.529 |
| GGGATTTTTA | 1.386 | 0.811 | 0.704 | 0.529 | GGTGTTTATC | -0.125 | 0.783 | 0.631 | 0.569 |
| GGGCTTTTTC | 1.382 | 0.798 | 0.739 | 0.624 | GGCATTTTTC | -0.125 | 0.794 | 0.686 | 0.502 |
| GGGGCTGCCC | 1.380 | 0.907 | 0.973 | 0.898 | GGGGTCTTGC | -0.125 | 0.765 | 0.755 | 0.638 |
| AGGGATTTCC | 1.380 | 0.860 | 0.854 | 0.790 | GGGGTTTGCG | -0.125 | 0.767 | 0.858 | 0.735 |
| GGTGGTTTTA | 1.372 | 0.772 | 0.529 | 0.469 | TGAGGTTTCC | -0.125 | 0.793 | 0.638 | 0.575 |
| CGGGTTTCCC | 1.365 | 0.886 | 0.851 | 0.790 | GCTATTTCTC | -0.125 | 0.777 | 0.578 | 0.447 |
| AGGGGCTTCC | 1.362 | 0.751 | 0.688 | 0.633 | GGGGTTATGC | -0.125 | 0.766 | 0.798 | 0.685 |
| GGGAGTTTAC | 1.362 | 0.919 | 0.746 | 0.630 | GGTGGTTCAT | -0.125 | 0.810 | 0.549 | 0.546 |
| GGAGAGTCCC | 1.362 | 0.831 | 0.864 | 0.807 | TTGATTTCCC | -0.125 | 0.802 | 0.705 | 0.580 |
| TGGATTCCCC | 1.362 | 0.816 | 0.831 | 0.704 | TGTACTTCTC | -0.125 | 0.792 | 0.580 | 0.447 |
| GGGGAGTACC | 1.362 | 0.786 | 0.898 | 0.852 | GGGCATTTCG | -0.125 | 0.779 | 0.861 | 0.734 |
| TGGGACTTCC | 1.362 | 0.761 | 0.777 | 0.715 | GGGGATTCGT | -0.137 | 0.789 | 0.765 | 0.761 |
| GGTAAGTCCC | 1.362 | 0.887 | 0.844 | 0.730 | GTTAGTTTCC | -0.137 | 0.842 | 0.619 | 0.497 |
| GGGGTTCCCT | 1.362 | 0.798 | 0.869 | 0.809 | GCGTGTTTCC | -0.137 | 0.761 | 0.672 | 0.619 |
| GGTTTTTCCT | 1.360 | 0.799 | 0.650 | 0.596 | GGGTATTTCG | -0.137 | 0.805 | 0.861 | 0.734 |
| GGGAATTTTC | 1.357 | 0.897 | 0.835 | 0.712 | GGTTCTGTCC | -0.137 | 0.794 | 0.734 | 0.599 |
| TGGGGTTCCC | 1.356 | 0.886 | 0.784 | 0.785 | TGTGTTGTCC | -0.137 | 0.783 | 0.698 | 0.554 |
| TGGTTTTCCC | 1.356 | 0.828 | 0.758 | 0.702 | GGGGTTTGGC | -0.137 | 0.762 | 0.758 | 0.702 |
| GTGTATTCCC | 1.352 | 0.817 | 0.781 | 0.779 | GGTATTGTTC | -0.143 | 0.815 | 0.679 | 0.477 |
| GGGAGAGTCC | 1.352 | 0.818 | 0.789 | 0.663 | GTTATTTTTC | -0.143 | 0.777 | 0.559 | 0.369 |
| GGATGTCCCC | 1.352 | 0.758 | 0.691 | 0.688 | TGTATTTCTC | -0.149 | 0.810 | 0.578 | 0.447 |
| GGGATTTTCT | 1.351 | 0.871 | 0.831 | 0.662 | GATATTTTTC | -0.149 | 0.765 | 0.559 | 0.369 |
| GGGGTTGTCC | 1.350 | 0.900 | 0.952 | 0.820 | GGTACTTTGC | -0.149 | 0.840 | 0.687 | 0.502 |
| GGGATTTTAG | 1.349 | 0.838 | 0.785 | 0.536 | GGGGCTTTGA | -0.149 | 0.762 | 0.725 | 0.607 |
| GGATTTTCCG | 1.346 | 0.766 | 0.731 | 0.602 | TGTATTTTGC | -0.149 | 0.763 | 0.559 | 0.369 |
| GGTGGTCCCC | 1.342 | 0.871 | 0.783 | 0.777 | TGTGTCTCCC | -0.149 | 0.769 | 0.647 | 0.582 |
| CGGACTTTCC | 1.342 | 0.852 | 0.814 | 0.635 | GGGGGTTCAT | -0.149 | 0.832 | 0.675 | 0.679 |
| TGGACTTCCC | 1.342 | 0.887 | 0.833 | 0.713 | GGTAGTGTTC | -0.155 | 0.806 | 0.612 | 0.472 |
| GGGGGATCTC | 1.342 | 0.825 | 0.707 | 0.710 | AGTGCTTTTC | -0.155 | 0.751 | 0.580 | 0.447 |
| CGGGGCTTCC | 1.342 | 0.747 | 0.688 | 0.633 | TGGGGTTTGC | -0.155 | 0.766 | 0.638 | 0.575 |
| GTTTTTTCCC | 1.338 | 0.799 | 0.631 | 0.569 | GGTTCTTCTC | -0.155 | 0.810 | 0.633 | 0.569 |
| GGTGTTCTCC | 1.337 | 0.854 | 0.831 | 0.704 | GTGGGTGTCC | -0.155 | 0.788 | 0.758 | 0.682 |
| GGGGATTTAT | 1.334 | 0.811 | 0.746 | 0.683 | GGTGCTTCTG | -0.155 | 0.788 | 0.698 | 0.557 |
| GGTACTCTCC | 1.332 | 0.846 | 0.814 | 0.627 | GGTGGATTCT | -0.155 | 0.748 | 0.579 | 0.527 |
| GTTCTTTCCC | 1.327 | 0.774 | 0.631 | 0.569 | TGTGGGTTCC | -0.155 | 0.751 | 0.628 | 0.515 |
| TGGGTATTCC | 1.326 | 0.786 | 0.755 | 0.638 | GGTAGGTTCT | -0.155 | 0.753 | 0.627 | 0.464 |
| GGGGTTTTAG | 1.323 | 0.828 | 0.804 | 0.613 | GGTATGTTGC | -0.155 | 0.770 | 0.676 | 0.443 |
| GGTGCTTTTA | 1.322 | 0.762 | 0.598 | 0.474 | TCGGTTTCCC | -0.155 | 0.767 | 0.724 | 0.657 |
| GGGTCTCCCC | 1.322 | 0.816 | 0.887 | 0.826 | TGTGTTTCCA | -0.155 | 0.785 | 0.615 | 0.551 |
| CGGGTATTCC | 1.322 | 0.777 | 0.755 | 0.638 | GGTGATTTCG | -0.161 | 0.850 | 0.827 | 0.690 |
| GGGGTAGTCC | 1.322 | 0.816 | 0.875 | 0.746 | CGGGGTTTGC | -0.161 | 0.756 | 0.638 | 0.575 |
| GGGGGATTCT | 1.322 | 0.769 | 0.706 | 0.660 | GGGTTTGTTC | -0.161 | 0.759 | 0.732 | 0.599 |
| GGACATTCCC | 1.322 | 0.826 | 0.781 | 0.779 | GGGTCTTTCT | -0.167 | 0.776 | 0.759 | 0.651 |
| GGGACTTCAC | 1.322 | 0.935 | 0.833 | 0.713 | GGGTGTTTGC | -0.167 | 0.794 | 0.672 | 0.619 |
| GGGTTTTTTC | 1.319 | 0.824 | 0.739 | 0.624 | GGTGTTCTTC | -0.167 | 0.781 | 0.704 | 0.571 |
| GGTATTCCCT | 1.317 | 0.787 | 0.723 | 0.598 | GGTAATTTCG | -0.174 | 0.860 | 0.808 | 0.612 |
| GGGAGTTTTC | 1.317 | 0.892 | 0.746 | 0.630 | GTTGATCTCC | -0.174 | 0.748 | 0.727 | 0.648 |
| GGGTAGTTCC | 1.315 | 0.806 | 0.879 | 0.774 | GGAGTGTTCC | -0.174 | 0.808 | 0.822 | 0.653 |
| GGGGTTTTGC | 1.314 | 0.870 | 0.831 | 0.713 | AGGGCTTTCG | -0.174 | 0.756 | 0.806 | 0.613 |
| GGGGCTTACC | 1.312 | 0.859 | 0.887 | 0.835 | GGTAGGTCCT | -0.174 | 0.779 | 0.647 | 0.542 |
| GGGATTTCAC | 1.312 | 0.953 | 0.831 | 0.713 | GGGGTTACTC | -0.174 | 0.812 | 0.817 | 0.762 |
| GGGGGATTAC | 1.312 | 0.826 | 0.688 | 0.633 | GTGGATTTCA | -0.174 | 0.769 | 0.746 | 0.683 |
| GGGCGTTCCC | 1.312 | 0.889 | 0.818 | 0.830 | GGTCATTCCT | -0.174 | 0.770 | 0.672 | 0.672 |
| AGGGACTTCC | 1.312 | 0.756 | 0.777 | 0.715 | CGTGTTCCCC | -0.174 | 0.776 | 0.723 | 0.649 |
| GGGGGCTCCC | 1.312 | 0.877 | 0.834 | 0.843 | GGTGATTTCA | -0.174 | 0.851 | 0.746 | 0.683 |
| GGTTTTTGCC | 1.310 | 0.769 | 0.666 | 0.613 | AGTTGTTCCC | -0.174 | 0.793 | 0.565 | 0.564 |
| GGTTTTCTCC | 1.309 | 0.788 | 0.739 | 0.616 | GGTGCTCCCT | -0.174 | 0.758 | 0.744 | 0.676 |
| GGGCTTTCCT | 1.308 | 0.794 | 0.776 | 0.729 | AGTAGTTTCG | -0.174 | 0.755 | 0.591 | 0.397 |
| GGTTGTTCCC | 1.308 | 0.894 | 0.691 | 0.697 | TGTACTTCGC | -0.174 | 0.771 | 0.580 | 0.447 |
| GGGTTTTCAC | 1.305 | 0.877 | 0.758 | 0.702 | GGGATGTTAG | -0.174 | 0.750 | 0.775 | 0.476 |
| GGGTTTCCAC | 1.301 | 0.788 | 0.758 | 0.693 | GGGCGTGCTC | -0.174 | 0.751 | 0.685 | 0.671 |
| GGTTCTTCCC | 1.301 | 0.884 | 0.760 | 0.702 | ATTATTTTCC | -0.174 | 0.750 | 0.559 | 0.369 |
| TGGGGATCCC | 1.301 | 0.803 | 0.707 | 0.710 | GGGAATTGTC | -0.174 | 0.789 | 0.762 | 0.701 |
| GGGATTTTCA | 1.301 | 0.885 | 0.831 | 0.662 | CGGATTTTTC | -0.174 | 0.796 | 0.686 | 0.502 |
| GGAGTTTACC | 1.301 | 0.810 | 0.758 | 0.702 | GGTACTTTGA | -0.174 | 0.751 | 0.579 | 0.396 |
| GGGCTTTTCG | 1.299 | 0.783 | 0.838 | 0.657 | GGTAATTCTG | -0.174 | 0.812 | 0.700 | 0.557 |
| GGATTTTTCC | 1.299 | 0.830 | 0.739 | 0.624 | GGTAGTGCCT | -0.174 | 0.802 | 0.650 | 0.576 |
| GGGCTTTTGC | 1.299 | 0.777 | 0.739 | 0.624 | GGTGTATCCG | -0.174 | 0.796 | 0.746 | 0.616 |
| TGGGGGTTCC | 1.297 | 0.772 | 0.755 | 0.648 | TGTACTTCCG | -0.174 | 0.776 | 0.679 | 0.480 |
| GGGTGGTTCC | 1.296 | 0.801 | 0.789 | 0.692 | GTAATTTCTC | -0.174 | 0.756 | 0.578 | 0.447 |
| GGGCTTTTCT | 1.294 | 0.768 | 0.757 | 0.651 | CGTATGTTCC | -0.174 | 0.760 | 0.676 | 0.443 |
| GGTGGTTCAC | 1.294 | 0.914 | 0.657 | 0.652 | TGGCTTTCAC | -0.174 | 0.756 | 0.631 | 0.569 |
| GGGTTTTTGC | 1.291 | 0.803 | 0.739 | 0.624 | CGCGTTTCCC | -0.174 | 0.779 | 0.724 | 0.657 |
| GGTGTATCCC | 1.290 | 0.886 | 0.774 | 0.715 | CGGGCTTCTC | -0.174 | 0.794 | 0.726 | 0.657 |
| GGGTTATTCC | 1.290 | 0.814 | 0.789 | 0.683 | GCGAGTTTCG | -0.174 | 0.748 | 0.718 | 0.530 |
| GGATTTTCCT | 1.288 | 0.752 | 0.650 | 0.596 | GTGAGTGTCC | -0.174 | 0.798 | 0.739 | 0.605 |
| GGGTTTTTAT | 1.288 | 0.748 | 0.631 | 0.518 | GGGACTTCGG | -0.174 | 0.798 | 0.806 | 0.613 |
| TGGGTTTTCC | 1.286 | 0.869 | 0.831 | 0.713 | GGTATGTGCC | -0.174 | 0.757 | 0.729 | 0.565 |
| GGGGAGTTTC | 1.285 | 0.799 | 0.844 | 0.730 | GGGATTGCTC | -0.174 | 0.862 | 0.825 | 0.687 |
| GGTGTTTTTT | 1.283 | 0.766 | 0.596 | 0.474 | GGGATTGCGG | -0.174 | 0.751 | 0.798 | 0.587 |
| GGTGTTTTTC | 1.283 | 0.869 | 0.705 | 0.580 | GGTGTTTCAA | -0.174 | 0.833 | 0.615 | 0.551 |
| GGGATTTTTT | 1.280 | 0.797 | 0.704 | 0.529 | CGGGCTTCCT | -0.174 | 0.764 | 0.744 | 0.684 |
| GGGAAGTTCC | 1.279 | 0.883 | 0.952 | 0.785 | TGTATTGCCC | -0.174 | 0.819 | 0.698 | 0.554 |
| GGGAATACCG | 1.279 | 0.803 | 0.920 | 0.794 | CGTGTTGCCC | -0.174 | 0.800 | 0.717 | 0.632 |
| GGGTTTTTCG | 1.278 | 0.808 | 0.838 | 0.657 | GGCCGTTTCC | -0.174 | 0.757 | 0.672 | 0.619 |
| GGGGATTTCG | 1.275 | 0.872 | 0.954 | 0.823 | CGGGGTTCCT | -0.174 | 0.774 | 0.675 | 0.679 |
| GGGTGTTTTC | 1.272 | 0.815 | 0.672 | 0.619 | GGTGTTTACA | -0.174 | 0.767 | 0.650 | 0.596 |
| AGGGATTTTC | 1.271 | 0.787 | 0.727 | 0.657 | CGTATTTCGC | -0.174 | 0.780 | 0.578 | 0.447 |
| TGGATTTCCC | 1.270 | 0.905 | 0.831 | 0.713 | GGTACTTGTC | -0.174 | 0.753 | 0.614 | 0.491 |
| GGTGATCCCC | 1.267 | 0.877 | 0.873 | 0.859 | TGTGCTGTCC | -0.174 | 0.765 | 0.700 | 0.554 |
| GGGAGTTACC | 1.267 | 0.879 | 0.799 | 0.752 | GGGGGTGCAT | -0.174 | 0.767 | 0.669 | 0.653 |
| CGGGACTTCC | 1.267 | 0.752 | 0.777 | 0.715 | TGGATTTCCG | -0.174 | 0.816 | 0.804 | 0.613 |
| GGGTATTACC | 1.267 | 0.807 | 0.815 | 0.823 | GGTAATGTTC | -0.174 | 0.811 | 0.702 | 0.554 |
| GGGCTTACCC | 1.267 | 0.794 | 0.852 | 0.806 | TGTCCTTCCC | -0.174 | 0.763 | 0.633 | 0.569 |
| GGGGGACCCC | 1.267 | 0.809 | 0.833 | 0.835 | GGTACCTTTC | -0.174 | 0.756 | 0.611 | 0.428 |
| GGTATCCCCC | 1.267 | 0.785 | 0.754 | 0.630 | TGTGATCTCC | -0.174 | 0.756 | 0.727 | 0.648 |
| TGGTATTCCC | 1.267 | 0.825 | 0.781 | 0.779 | TGAGCTTTCC | -0.174 | 0.783 | 0.707 | 0.580 |
| GGGACTTTAC | 1.267 | 0.909 | 0.814 | 0.635 | GGTGATGCTC | -0.174 | 0.827 | 0.740 | 0.709 |
| GGGTATTTTC | 1.266 | 0.821 | 0.762 | 0.701 | CGTATTTTGC | -0.174 | 0.754 | 0.559 | 0.369 |
| GGTGCTGTCC | 1.259 | 0.860 | 0.827 | 0.687 | GGGTTGGCCC | -0.174 | 0.771 | 0.869 | 0.749 |
| GGGGGCGCCC | 1.255 | 0.812 | 0.827 | 0.818 | GGGGGGGCTC | -0.174 | 0.755 | 0.768 | 0.700 |
| AGGGCTTTCC | 1.255 | 0.846 | 0.833 | 0.713 | GTGGCTCTCC | -0.174 | 0.754 | 0.833 | 0.704 |
| GGGGATTTCA | 1.255 | 0.872 | 0.872 | 0.816 | GGAGGTTCCT | -0.174 | 0.810 | 0.675 | 0.679 |
| GGAAGTTCCC | 1.255 | 0.923 | 0.765 | 0.708 | GGCATTTTCG | -0.174 | 0.778 | 0.785 | 0.536 |
| GGTGTCCCCC | 1.255 | 0.775 | 0.773 | 0.707 | GGTATTGCAT | -0.174 | 0.764 | 0.590 | 0.448 |
| GGGGTTTCTC | 1.253 | 0.916 | 0.851 | 0.790 | GGCTTTTCAC | -0.174 | 0.770 | 0.631 | 0.569 |
| GGGATTTTGT | 1.253 | 0.776 | 0.704 | 0.529 | CGGGTTTCAG | -0.174 | 0.750 | 0.697 | 0.557 |
| GGTGTTTTAC | 1.251 | 0.897 | 0.705 | 0.580 | TGGGTTGTAC | -0.174 | 0.758 | 0.698 | 0.554 |
| GGTGTATTCC | 1.250 | 0.860 | 0.755 | 0.638 | GGTGGTTCCA | -0.174 | 0.871 | 0.675 | 0.679 |
| GGGCCTTTCC | 1.247 | 0.854 | 0.868 | 0.757 | GGGGATGCTC | -0.174 | 0.848 | 0.867 | 0.842 |
| GGTACTGTCC | 1.247 | 0.870 | 0.808 | 0.610 | GGTATTTAGC | -0.174 | 0.772 | 0.612 | 0.491 |
| GGGGCTTTCT | 1.246 | 0.843 | 0.852 | 0.740 | CGGCGTTTCC | -0.174 | 0.759 | 0.672 | 0.619 |
| GGGGGTTTAT | 1.245 | 0.806 | 0.656 | 0.602 | GGTATCTCCT | -0.174 | 0.771 | 0.646 | 0.532 |
| GTGGTCTCCC | 1.243 | 0.783 | 0.774 | 0.715 | TGTAGTCTCC | -0.174 | 0.760 | 0.618 | 0.489 |
| GGGGATTCTT | 1.242 | 0.810 | 0.765 | 0.761 | TGTGTTGCCC | -0.174 | 0.809 | 0.717 | 0.632 |
| GGTTTTGCCC | 1.241 | 0.838 | 0.752 | 0.676 | GCTGTGTCCC | -0.174 | 0.753 | 0.714 | 0.597 |
| GTGTTTTTCC | 1.240 | 0.795 | 0.739 | 0.624 | CGGAGTTTGC | -0.174 | 0.766 | 0.619 | 0.497 |
| GGGTTTGCCC | 1.239 | 0.859 | 0.878 | 0.809 | AGGGTTTCCG | -0.174 | 0.800 | 0.823 | 0.690 |
| GGTGCTTTAC | 1.235 | 0.878 | 0.707 | 0.580 | GGTGTTACCG | -0.174 | 0.776 | 0.790 | 0.662 |
| GGGACTTTCT | 1.234 | 0.853 | 0.833 | 0.662 | GGAGTTTGCC | -0.174 | 0.789 | 0.758 | 0.702 |
| GGCCTTTCCC | 1.230 | 0.791 | 0.758 | 0.702 | GGTCTTTCAC | -0.174 | 0.830 | 0.631 | 0.569 |
| GTGATTTACC | 1.230 | 0.785 | 0.739 | 0.624 | GGGGATCTCG | -0.174 | 0.783 | 0.953 | 0.814 |
| GGGAATTCCA | 1.230 | 0.908 | 0.872 | 0.816 | GGGACGTTCT | -0.174 | 0.764 | 0.823 | 0.602 |
| GGGGGAGTAC | 1.230 | 0.761 | 0.681 | 0.607 | TGGTCTTCAC | -0.174 | 0.764 | 0.633 | 0.569 |
| GGATCTCCCC | 1.230 | 0.748 | 0.760 | 0.693 | GGGAGTTTGA | -0.174 | 0.782 | 0.637 | 0.524 |
| GGTGTTTACC | 1.230 | 0.856 | 0.758 | 0.702 | GGGTGTCTAC | -0.174 | 0.754 | 0.672 | 0.611 |
| CGGGGATCCC | 1.230 | 0.794 | 0.707 | 0.710 | GTTGTATCCC | -0.174 | 0.783 | 0.647 | 0.582 |
| GGTACTTTAC | 1.230 | 0.888 | 0.687 | 0.502 | GGTACTTCCG | -0.174 | 0.871 | 0.806 | 0.613 |
| GGTTTTTCAC | 1.223 | 0.856 | 0.631 | 0.569 | GGGTATTCTA | -0.174 | 0.757 | 0.672 | 0.672 |
| GGTTTTTTAC | 1.221 | 0.830 | 0.612 | 0.491 | GCGGCTTTCC | -0.174 | 0.818 | 0.833 | 0.713 |
| GGGTTTTTCA | 1.221 | 0.809 | 0.757 | 0.651 | GGTAATGTCT | -0.174 | 0.782 | 0.720 | 0.581 |
| GGGATTTTGG | 1.220 | 0.790 | 0.785 | 0.536 | GTGGGTTACC | -0.174 | 0.766 | 0.691 | 0.697 |
| GGGTTTTTAG | 1.218 | 0.762 | 0.712 | 0.524 | GTGGGTATCC | -0.174 | 0.749 | 0.731 | 0.679 |
| GGTGCTGCCC | 1.217 | 0.886 | 0.846 | 0.765 | GGGTATTCAT | -0.174 | 0.770 | 0.672 | 0.672 |
| GGTTGTCCCC | 1.217 | 0.805 | 0.691 | 0.688 | GGTTTGGCCC | -0.174 | 0.749 | 0.742 | 0.616 |
| GGGGCTTCAC | 1.217 | 0.925 | 0.853 | 0.790 | TGGGATTCCT | -0.174 | 0.788 | 0.765 | 0.761 |
| TGGGGCTTCC | 1.217 | 0.756 | 0.688 | 0.633 | GATGCTTTCC | -0.174 | 0.811 | 0.707 | 0.580 |
| GGGAATTCCG | 1.217 | 0.907 | 0.954 | 0.823 | GGTAGTTGCC | -0.174 | 0.837 | 0.672 | 0.619 |
| GGGACATTCC | 1.217 | 0.873 | 0.864 | 0.694 | TGGGCTCTCC | -0.174 | 0.762 | 0.833 | 0.704 |
| GGGGAATTCT | 1.213 | 0.774 | 0.796 | 0.742 | CGTGCTTTAC | -0.174 | 0.774 | 0.580 | 0.447 |
| GGTATTCCCG | 1.213 | 0.801 | 0.804 | 0.604 | AGTGCTTCCT | -0.174 | 0.747 | 0.617 | 0.551 |
| GGGAATTTTT | 1.212 | 0.794 | 0.726 | 0.606 | GATTATTTCC | -0.174 | 0.759 | 0.635 | 0.568 |
| GGGGTCTTCC | 1.209 | 0.860 | 0.881 | 0.771 | GCGGGTTTAC | -0.174 | 0.781 | 0.638 | 0.575 |
| GGGTTTTTCT | 1.207 | 0.794 | 0.757 | 0.651 | TGTGCATTCC | -0.174 | 0.747 | 0.630 | 0.505 |
| GGTTCTTTCC | 1.205 | 0.858 | 0.741 | 0.624 | GTTGTTACCC | -0.174 | 0.762 | 0.690 | 0.629 |
| GGGGGTTTCA | 1.204 | 0.867 | 0.783 | 0.735 | GTTGGCTCCC | -0.174 | 0.753 | 0.580 | 0.577 |
| GGATATCCCC | 1.204 | 0.763 | 0.780 | 0.770 | CGGATTTCCT | -0.174 | 0.792 | 0.723 | 0.607 |
| CGGGATTTTC | 1.204 | 0.783 | 0.727 | 0.657 | TGTGGTCCCC | -0.174 | 0.776 | 0.657 | 0.644 |
| GGTGCTCTCC | 1.204 | 0.836 | 0.833 | 0.704 | GGGAATTCGT | -0.174 | 0.798 | 0.746 | 0.683 |
| GGGATTCCTC | 1.204 | 0.837 | 0.831 | 0.704 | GGTCTTTCCG | -0.174 | 0.787 | 0.731 | 0.602 |
| AGGGATTCCC | 1.204 | 0.886 | 0.873 | 0.867 | GGTATTGGCC | -0.174 | 0.781 | 0.732 | 0.599 |
| GATTTTTCCC | 1.196 | 0.788 | 0.631 | 0.569 | CGTGTTTACC | -0.174 | 0.752 | 0.631 | 0.569 |
| TGGGTTTCCC | 1.196 | 0.895 | 0.851 | 0.790 | GTTGGTCCCC | -0.174 | 0.769 | 0.657 | 0.644 |
| GGGAATTTTG | 1.194 | 0.808 | 0.808 | 0.612 | CGTATTTCCC | -0.174 | 0.874 | 0.705 | 0.580 |
| GGTATTTACC | 1.190 | 0.866 | 0.739 | 0.624 | CGGGTTGTAC | -0.174 | 0.749 | 0.698 | 0.554 |
| TGGGCTTCCC | 1.190 | 0.877 | 0.853 | 0.790 | CGTAGTTTCG | -0.174 | 0.751 | 0.591 | 0.397 |
| GGTTTTTCGC | 1.190 | 0.808 | 0.631 | 0.569 | GGTACGTTGC | -0.174 | 0.752 | 0.678 | 0.443 |
| GGGGTTCCCA | 1.190 | 0.812 | 0.869 | 0.809 | TGTAGTTCCT | -0.174 | 0.772 | 0.529 | 0.469 |
| GTGGTTTCCA | 1.188 | 0.798 | 0.742 | 0.684 | GGTGTTTGTC | -0.180 | 0.762 | 0.631 | 0.569 |
| GGAGTTTTCC | 1.188 | 0.896 | 0.831 | 0.713 | TGTGGTTCCT | -0.180 | 0.762 | 0.549 | 0.546 |
| GGGTGCTTCC | 1.184 | 0.784 | 0.722 | 0.677 | GGTTCTTTCT | -0.180 | 0.755 | 0.633 | 0.518 |
| GGGAGTTTTT | 1.181 | 0.789 | 0.637 | 0.524 | TGTGGTTCTC | -0.180 | 0.792 | 0.530 | 0.519 |
| GGGGTTTTGT | 1.178 | 0.766 | 0.723 | 0.607 | GGTGTCTTTC | -0.180 | 0.765 | 0.628 | 0.505 |
| GGGACTGCAC | 1.176 | 0.871 | 0.827 | 0.687 | GGTGGTTGTC | -0.187 | 0.753 | 0.565 | 0.564 |
| GGGGATCTAC | 1.176 | 0.825 | 0.854 | 0.781 | TGGGTTTCCT | -0.187 | 0.792 | 0.742 | 0.684 |
| GGGAATTCCT | 1.176 | 0.893 | 0.872 | 0.816 | GCTGTTTTCC | -0.187 | 0.815 | 0.705 | 0.580 |
| GGGATCTACC | 1.176 | 0.783 | 0.789 | 0.683 | GGTATTTGTC | -0.187 | 0.772 | 0.612 | 0.491 |
| GGGAGGTCCC | 1.176 | 0.903 | 0.882 | 0.781 | TGGGTTTGCC | -0.187 | 0.761 | 0.758 | 0.702 |
| GGATTTTCCA | 1.176 | 0.766 | 0.650 | 0.596 | TGTGCTTCGC | -0.194 | 0.761 | 0.599 | 0.524 |
| GGTTTTTTGC | 1.171 | 0.782 | 0.612 | 0.491 | GCTGGTTTCC | -0.194 | 0.807 | 0.638 | 0.575 |
| GGTTTTGCCA | 1.171 | 0.749 | 0.643 | 0.570 | GGGGGTCTCT | -0.194 | 0.763 | 0.783 | 0.726 |
| GTGCTTTTCC | 1.170 | 0.769 | 0.739 | 0.624 | GTTGTTTACC | -0.194 | 0.754 | 0.631 | 0.569 |
| GGTTTTTTCA | 1.170 | 0.788 | 0.631 | 0.518 | GGTGTTGCCG | -0.194 | 0.815 | 0.817 | 0.665 |
| GGGTTTCCTC | 1.166 | 0.761 | 0.758 | 0.693 | CGTGGTTCCT | -0.194 | 0.752 | 0.549 | 0.546 |
| GGTGCTCCCC | 1.161 | 0.862 | 0.852 | 0.782 | CGTGTTTTAC | -0.201 | 0.792 | 0.578 | 0.447 |
| GGGATTTACG | 1.161 | 0.798 | 0.838 | 0.657 | GGTGTTTCGC | -0.201 | 0.874 | 0.724 | 0.657 |
| GGGCGGTCCC | 1.161 | 0.801 | 0.809 | 0.770 | GGTACTTTGG | -0.201 | 0.751 | 0.660 | 0.403 |
| GGGAGTGTCC | 1.161 | 0.901 | 0.866 | 0.738 | GGTCTTTTCA | -0.201 | 0.762 | 0.631 | 0.518 |
| GGGGATTCCA | 1.161 | 0.898 | 0.892 | 0.894 | GGGGTTGTAG | -0.208 | 0.764 | 0.798 | 0.587 |
| TGTACTTCCC | 1.161 | 0.866 | 0.707 | 0.580 | GGTGGTGTTC | -0.208 | 0.796 | 0.631 | 0.549 |
| GCTTTTTTCC | 1.157 | 0.749 | 0.612 | 0.491 | GTTGGTTCCC | -0.208 | 0.858 | 0.657 | 0.652 |
| GGTTTTGCCG | 1.157 | 0.748 | 0.724 | 0.576 | GGTGTTATTC | -0.208 | 0.765 | 0.671 | 0.552 |
| CGGGCTTTCC | 1.156 | 0.842 | 0.833 | 0.713 | GGTGTTTTGA | -0.215 | 0.759 | 0.596 | 0.474 |
| TGGGAGTTCC | 1.154 | 0.778 | 0.844 | 0.730 | GGGTGTTTCG | -0.215 | 0.800 | 0.772 | 0.652 |
| GGTGATGTCC | 1.154 | 0.875 | 0.848 | 0.764 | GTTTATTTCC | -0.215 | 0.770 | 0.635 | 0.568 |
| GGGTTTTCTC | 1.154 | 0.850 | 0.758 | 0.702 | GGGGTGTGCC | -0.222 | 0.768 | 0.875 | 0.775 |
| TGTTTTTCCC | 1.151 | 0.807 | 0.631 | 0.569 | GTTATTTCTC | -0.222 | 0.802 | 0.578 | 0.447 |
| GGTGGTGTCC | 1.151 | 0.870 | 0.758 | 0.682 | GGGTGTCTCC | -0.222 | 0.800 | 0.799 | 0.744 |
| GGGATTTCTT | 1.150 | 0.823 | 0.723 | 0.607 | GGTATTTCAT | -0.222 | 0.829 | 0.596 | 0.474 |
| GGTGGTTTTG | 1.147 | 0.772 | 0.611 | 0.475 | GGCATTTTGC | -0.222 | 0.773 | 0.686 | 0.502 |
| GGGGCGGTCC | 1.146 | 0.793 | 0.944 | 0.760 | CGCGTTTTCC | -0.222 | 0.753 | 0.705 | 0.580 |
| GGGACGGCCC | 1.146 | 0.829 | 0.944 | 0.760 | GGGGGTGCCT | -0.222 | 0.814 | 0.796 | 0.786 |
| GGTGATGCCC | 1.146 | 0.901 | 0.867 | 0.842 | GCGGGTTCTC | -0.222 | 0.780 | 0.657 | 0.652 |
| CGGGCTTCCC | 1.146 | 0.867 | 0.853 | 0.790 | GGTATGTCTC | -0.222 | 0.817 | 0.695 | 0.520 |
| GGTGCGTCCC | 1.146 | 0.863 | 0.843 | 0.730 | TGGGCTTTAC | -0.222 | 0.804 | 0.707 | 0.580 |
| GGTAGTCCCC | 1.146 | 0.881 | 0.764 | 0.699 | AGTTCTTTCC | -0.222 | 0.758 | 0.614 | 0.491 |
| GGTGATTTAC | 1.146 | 0.893 | 0.727 | 0.657 | TGGAGTTTGC | -0.222 | 0.776 | 0.619 | 0.497 |
| GGGCTTTCCA | 1.146 | 0.809 | 0.776 | 0.729 | GGTCCTTCCT | -0.222 | 0.755 | 0.652 | 0.596 |
| GGAGTGTCCC | 1.146 | 0.834 | 0.841 | 0.730 | GGGAGGTTTC | -0.222 | 0.804 | 0.736 | 0.570 |
| GGGACGGTCC | 1.146 | 0.803 | 0.925 | 0.683 | GATATTTTCG | -0.222 | 0.749 | 0.658 | 0.403 |
| GGGGTATTTC | 1.139 | 0.807 | 0.755 | 0.638 | GGGTTGTTAC | -0.222 | 0.763 | 0.729 | 0.565 |
| GGTTTTTCAG | 1.139 | 0.766 | 0.604 | 0.469 | TGGGTTTCAC | -0.222 | 0.848 | 0.724 | 0.657 |
| GGGGTGTTCC | 1.138 | 0.876 | 0.949 | 0.786 | GTCGTTTCCC | -0.222 | 0.781 | 0.724 | 0.657 |
| GGGGCGTTCC | 1.136 | 0.858 | 0.951 | 0.786 | CGTACTTTTC | -0.222 | 0.757 | 0.561 | 0.369 |
| GGTTTTATCC | 1.132 | 0.772 | 0.706 | 0.596 | GGGGCTTCGC | -0.222 | 0.877 | 0.853 | 0.790 |
| GGTTTTTTCG | 1.131 | 0.787 | 0.712 | 0.524 | GGGCTTGCTC | -0.222 | 0.759 | 0.752 | 0.676 |
| GTGGTTTCCG | 1.130 | 0.798 | 0.823 | 0.690 | GGTGGGGTCC | -0.222 | 0.782 | 0.748 | 0.622 |
| GGGGCCTCCC | 1.130 | 0.867 | 0.903 | 0.848 | TGTGTTGCAC | -0.222 | 0.763 | 0.590 | 0.499 |
| GGGGTGTACC | 1.130 | 0.789 | 0.875 | 0.775 | GGTGGTCTTC | -0.222 | 0.772 | 0.638 | 0.566 |
| GTAGATTCCC | 1.130 | 0.816 | 0.746 | 0.734 | GGTGTTTCAT | -0.222 | 0.819 | 0.615 | 0.551 |
| AGGGTTTTTC | 1.128 | 0.790 | 0.705 | 0.580 | TGGACTTTGC | -0.222 | 0.766 | 0.687 | 0.502 |
| TGTACTTTCC | 1.127 | 0.840 | 0.687 | 0.502 | CGGATTTTGC | -0.222 | 0.775 | 0.686 | 0.502 |
| GGTTTTTCAT | 1.127 | 0.752 | 0.523 | 0.463 | GGTTATTTCG | -0.222 | 0.784 | 0.734 | 0.601 |
| GGTGCTTTTG | 1.125 | 0.762 | 0.679 | 0.480 | GGTATCTTGC | -0.222 | 0.754 | 0.609 | 0.428 |
| GGGGTTATCC | 1.122 | 0.860 | 0.925 | 0.817 | GGTGGCTCCT | -0.222 | 0.752 | 0.599 | 0.604 |
| GGGCCTCCCC | 1.114 | 0.791 | 0.887 | 0.826 | GGTATTGCTC | -0.222 | 0.841 | 0.698 | 0.554 |
| GGGGCCTTCC | 1.114 | 0.841 | 0.883 | 0.771 | TGTACTTCCT | -0.222 | 0.762 | 0.598 | 0.474 |
| GGGACTATCC | 1.114 | 0.852 | 0.908 | 0.740 | TGTATCTTCC | -0.222 | 0.753 | 0.609 | 0.428 |
| GGAATTTTCC | 1.114 | 0.906 | 0.812 | 0.635 | TGTGGTGCCC | -0.222 | 0.801 | 0.650 | 0.627 |
| AGGGTGTTCC | 1.114 | 0.776 | 0.822 | 0.653 | GGGGGTTACG | -0.222 | 0.780 | 0.791 | 0.730 |
| GTGACTTCCC | 1.114 | 0.879 | 0.833 | 0.713 | CGTGCTTCTC | -0.222 | 0.773 | 0.599 | 0.524 |
| GGGCTTTTCA | 1.107 | 0.783 | 0.757 | 0.651 | GGTGGGTCCT | -0.222 | 0.769 | 0.666 | 0.619 |
| GGTGTTTCAC | 1.107 | 0.922 | 0.724 | 0.657 | TGGAGTTTCT | -0.222 | 0.767 | 0.637 | 0.524 |
| GGGGTTTTGG | 1.105 | 0.780 | 0.804 | 0.613 | GTAGGTTTCC | -0.222 | 0.785 | 0.638 | 0.575 |
| GGGATTTCTG | 1.103 | 0.837 | 0.804 | 0.613 | GGTGATCTTC | -0.222 | 0.777 | 0.727 | 0.648 |
| GTGGCTTCCC | 1.103 | 0.869 | 0.853 | 0.790 | TGTGGTTTAC | -0.222 | 0.793 | 0.511 | 0.442 |
| GGTTTTTTCT | 1.101 | 0.773 | 0.631 | 0.518 | TGGATGTTCC | -0.222 | 0.791 | 0.803 | 0.576 |
| GGTACATTCC | 1.097 | 0.852 | 0.737 | 0.561 | GGTCTTTTGC | -0.229 | 0.756 | 0.612 | 0.491 |
| GGGATATTCC | 1.097 | 0.891 | 0.862 | 0.694 | TTGATTTTCC | -0.229 | 0.776 | 0.686 | 0.502 |
| CGGACTTCCC | 1.097 | 0.877 | 0.833 | 0.713 | GGTGTGTTCC | -0.229 | 0.855 | 0.822 | 0.653 |
| GTGGTTTCCT | 1.097 | 0.784 | 0.742 | 0.684 | GGGTCTTTCG | -0.229 | 0.790 | 0.840 | 0.657 |
| GAGTTTTCCC | 1.097 | 0.809 | 0.758 | 0.702 | TGTGCTTCTC | -0.237 | 0.782 | 0.599 | 0.524 |
| AGGGAGTTCC | 1.097 | 0.772 | 0.844 | 0.730 | GGGATTGCTT | -0.237 | 0.758 | 0.716 | 0.581 |
| GGTGACTTCC | 1.097 | 0.835 | 0.777 | 0.715 | GGTCGTTTCC | -0.237 | 0.842 | 0.672 | 0.619 |
| GGTAGATTCC | 1.097 | 0.861 | 0.669 | 0.556 | GGTGCTCTTC | -0.237 | 0.762 | 0.706 | 0.571 |
| GGTTTTTCTC | 1.096 | 0.829 | 0.631 | 0.569 | GGGTTGTCTC | -0.244 | 0.761 | 0.749 | 0.642 |
| GGTTTTGCAC | 1.091 | 0.791 | 0.625 | 0.543 | GTTGTCTCCC | -0.244 | 0.761 | 0.647 | 0.582 |
| GGATTTTCGC | 1.091 | 0.761 | 0.631 | 0.569 | GGGGTTTAGC | -0.244 | 0.783 | 0.758 | 0.702 |
| GTTGTTTCCC | 1.088 | 0.866 | 0.724 | 0.657 | GGGGGTCTGC | -0.244 | 0.772 | 0.764 | 0.699 |
| GGATATTCCC | 1.079 | 0.852 | 0.781 | 0.779 | TGTTCTTTCC | -0.244 | 0.763 | 0.614 | 0.491 |
| GGTCCTTCCC | 1.079 | 0.858 | 0.760 | 0.702 | GGGGCTCTCT | -0.244 | 0.754 | 0.851 | 0.731 |
| GGTAGTCCCT | 1.079 | 0.778 | 0.656 | 0.593 | GGGGTGTTCG | -0.244 | 0.787 | 0.921 | 0.686 |
| GGATTTACCC | 1.079 | 0.751 | 0.725 | 0.673 | TGTCTTTTCC | -0.244 | 0.756 | 0.612 | 0.491 |
| GGTGCTTACC | 1.079 | 0.838 | 0.760 | 0.702 | GGGGCTTGCG | -0.244 | 0.749 | 0.860 | 0.735 |
| CGGATTTCCC | 1.079 | 0.895 | 0.831 | 0.713 | TGTTGTTCCC | -0.244 | 0.799 | 0.565 | 0.564 |
| GGTGATTTTC | 1.079 | 0.866 | 0.727 | 0.657 | GGTATTTCGT | -0.244 | 0.781 | 0.596 | 0.474 |
| GGGGGATGCC | 1.079 | 0.765 | 0.741 | 0.755 | GGGGTTGTCT | -0.252 | 0.796 | 0.843 | 0.714 |
| GGGGAGTTAC | 1.079 | 0.826 | 0.844 | 0.730 | CGGTTTTTAC | -0.252 | 0.747 | 0.612 | 0.491 |
| GGGGAGGTCC | 1.079 | 0.808 | 0.965 | 0.837 | GTGACTTTTC | -0.252 | 0.779 | 0.687 | 0.502 |
| CGTACTTTCC | 1.079 | 0.830 | 0.687 | 0.502 | GGCATTTTCT | -0.252 | 0.764 | 0.704 | 0.529 |
| GGTATTACCC | 1.079 | 0.875 | 0.798 | 0.685 | GGGGGTTCGC | -0.252 | 0.887 | 0.784 | 0.785 |
| GGGGATTCCG | 1.079 | 0.897 | 0.973 | 0.900 | TGTTGTTCAC | -0.252 | 0.752 | 0.438 | 0.431 |
| GGGATAGTCC | 1.079 | 0.826 | 0.856 | 0.668 | GGGGGCTTCG | -0.252 | 0.762 | 0.787 | 0.666 |
| GGGGGGATCC | 1.079 | 0.763 | 0.848 | 0.753 | GTTCTTTTCC | -0.252 | 0.748 | 0.612 | 0.491 |
| GGGATCTTCC | 1.079 | 0.870 | 0.862 | 0.694 | TGGAGTTTTC | -0.252 | 0.797 | 0.619 | 0.497 |
| GGGTTTTGCC | 1.076 | 0.790 | 0.793 | 0.746 | CGGGTTTTCT | -0.252 | 0.756 | 0.723 | 0.607 |
| GGTGTTTTTA | 1.072 | 0.780 | 0.596 | 0.474 | GTTATTTTCG | -0.260 | 0.761 | 0.658 | 0.403 |
| GGGGGTTCTC | 1.071 | 0.908 | 0.784 | 0.785 | GCGTTTTTCC | -0.260 | 0.770 | 0.739 | 0.624 |
| GGGTATGTCC | 1.070 | 0.830 | 0.882 | 0.809 | GGTGTTTGAC | -0.260 | 0.789 | 0.631 | 0.569 |
| AGGTTTTCCC | 1.070 | 0.823 | 0.758 | 0.702 | GGTGTTGCAT | -0.260 | 0.754 | 0.609 | 0.526 |
| GTGATTTTCC | 1.064 | 0.871 | 0.812 | 0.635 | TGGGTTTCCG | -0.260 | 0.806 | 0.823 | 0.690 |
| GGATGTTCCC | 1.061 | 0.847 | 0.691 | 0.697 | GGGTGTTCCT | -0.260 | 0.811 | 0.710 | 0.723 |
| GGGAGTTTTA | 1.061 | 0.803 | 0.637 | 0.524 | GGTGCTTTGC | -0.260 | 0.830 | 0.707 | 0.580 |
| GGGGTTTTAA | 1.061 | 0.829 | 0.723 | 0.607 | TGGCTTTTCC | -0.260 | 0.777 | 0.739 | 0.624 |
| GGGTTTTCTG | 1.059 | 0.760 | 0.731 | 0.602 | GGTGTTTAGC | -0.260 | 0.762 | 0.631 | 0.569 |
| TGGTATTTCC | 1.058 | 0.799 | 0.762 | 0.701 | GGTATGTTTC | -0.260 | 0.791 | 0.676 | 0.443 |
| TGGGCTTTCC | 1.058 | 0.851 | 0.833 | 0.713 | GGTGATTTGC | -0.260 | 0.845 | 0.727 | 0.657 |
| GGGATTTTGA | 1.054 | 0.790 | 0.704 | 0.529 | TGGGGTTTCG | -0.268 | 0.771 | 0.737 | 0.608 |
| GGGATTTATC | 1.054 | 0.814 | 0.739 | 0.624 | GGTGCTTTAG | -0.268 | 0.789 | 0.679 | 0.480 |
| GGGGATTCTA | 1.054 | 0.824 | 0.765 | 0.761 | TGTGTTTCCG | -0.268 | 0.785 | 0.697 | 0.557 |
| GCTTTTTCCC | 1.053 | 0.774 | 0.631 | 0.569 | GGTAGTTTCT | -0.268 | 0.841 | 0.637 | 0.524 |
| GGTACTTTTG | 1.051 | 0.772 | 0.660 | 0.403 | GGTCTTTTCT | -0.268 | 0.747 | 0.631 | 0.518 |
| GTGGATTTCC | 1.048 | 0.858 | 0.854 | 0.790 | TGTGTTTTCA | -0.268 | 0.759 | 0.596 | 0.474 |
| TGGTGTTTCC | 1.041 | 0.794 | 0.672 | 0.619 | GGGGTTGTAT | -0.268 | 0.750 | 0.716 | 0.581 |
| GGGATTCCTG | 1.041 | 0.748 | 0.804 | 0.604 | TGGGATTTGC | -0.268 | 0.771 | 0.727 | 0.657 |
| GGTGCTTCAC | 1.041 | 0.904 | 0.726 | 0.657 | GGGGTTGCCT | -0.268 | 0.822 | 0.862 | 0.792 |
| GTGGCGTCCC | 1.041 | 0.781 | 0.843 | 0.730 | GGTGTTTACG | -0.268 | 0.767 | 0.731 | 0.602 |
| GGGGCCGCCC | 1.041 | 0.803 | 0.896 | 0.823 | GAGGTTTTCT | -0.276 | 0.747 | 0.723 | 0.607 |
| GGGGATATTC | 1.041 | 0.783 | 0.821 | 0.761 | GGGGCTTCGT | -0.276 | 0.774 | 0.744 | 0.684 |
| TGAATTCCCC | 1.041 | 0.748 | 0.704 | 0.571 | GGTTATTTGC | -0.284 | 0.778 | 0.635 | 0.568 |
| GGTACTTACC | 1.041 | 0.848 | 0.741 | 0.624 | GGTGCTGTTC | -0.284 | 0.787 | 0.700 | 0.554 |
| GAGATTCCCC | 1.041 | 0.797 | 0.831 | 0.704 | GTTATTTTCT | -0.284 | 0.747 | 0.577 | 0.396 |
| GGGAGTCCCT | 1.041 | 0.799 | 0.783 | 0.726 | GGCGGTTTTC | -0.284 | 0.775 | 0.638 | 0.575 |
| GGTTTATCCC | 1.041 | 0.819 | 0.681 | 0.627 | TGTGTTTTAC | -0.292 | 0.802 | 0.578 | 0.447 |
| GGGAATTTCT | 1.041 | 0.867 | 0.853 | 0.739 | GTTATTTTGC | -0.292 | 0.756 | 0.559 | 0.369 |
| GGTGGTGCCC | 1.041 | 0.896 | 0.777 | 0.760 | GGTGCTACGC | -0.301 | 0.752 | 0.692 | 0.629 |
| GGGATTTCGC | 1.041 | 0.905 | 0.831 | 0.713 | GGTGGGTCCA | -0.301 | 0.783 | 0.666 | 0.619 |
| GGTTTTTTTC | 1.039 | 0.803 | 0.612 | 0.491 | GGTGTATCAA | -0.301 | 0.750 | 0.539 | 0.476 |
| GGATTTTCTC | 1.033 | 0.782 | 0.631 | 0.569 | GGAGTTCTCC | -0.301 | 0.807 | 0.831 | 0.704 |
| GGGTTTTCGC | 1.032 | 0.829 | 0.758 | 0.702 | GGTGTGGTCC | -0.301 | 0.790 | 0.815 | 0.627 |
| TGATTTTCCC | 1.030 | 0.760 | 0.631 | 0.569 | TGGAATTCGC | -0.301 | 0.807 | 0.727 | 0.657 |
| GGGATTTACT | 1.028 | 0.784 | 0.757 | 0.651 | GTCGGTTCCC | -0.301 | 0.772 | 0.657 | 0.652 |
| GGTATTTTAC | 1.021 | 0.907 | 0.686 | 0.502 | GGCATTTTAC | -0.301 | 0.821 | 0.686 | 0.502 |
| GGGGTTCCAC | 1.021 | 0.855 | 0.850 | 0.782 | AGTGATTCGC | -0.301 | 0.770 | 0.620 | 0.601 |
| GGGAATTTTA | 1.021 | 0.808 | 0.726 | 0.606 | CGTAATTCTC | -0.301 | 0.797 | 0.601 | 0.524 |
| GGGAATTTAT | 1.021 | 0.821 | 0.726 | 0.606 | TGGATATTAC | -0.301 | 0.749 | 0.609 | 0.428 |
| GGTGATTACC | 1.021 | 0.853 | 0.781 | 0.779 | TGGAATGTCC | -0.301 | 0.811 | 0.828 | 0.687 |
| GGGGACTTCT | 1.014 | 0.753 | 0.796 | 0.742 | TGTGATCCCC | -0.301 | 0.782 | 0.746 | 0.726 |
| GGGTATTTAC | 1.014 | 0.848 | 0.762 | 0.701 | GAGGAGTTCC | -0.301 | 0.758 | 0.844 | 0.730 |
| GGGTTTTCTA | 1.011 | 0.761 | 0.650 | 0.596 | GGCATTCTCC | -0.301 | 0.779 | 0.812 | 0.627 |
| GGTACTTTTA | 1.009 | 0.772 | 0.579 | 0.396 | GTGAACTTCC | -0.301 | 0.763 | 0.758 | 0.637 |
| GTGGTTTTTC | 1.008 | 0.788 | 0.705 | 0.580 | GGTAGTTATC | -0.301 | 0.784 | 0.545 | 0.486 |
| GGGAATTTAC | 1.000 | 0.924 | 0.835 | 0.712 | GGGGCATCAG | -0.301 | 0.753 | 0.748 | 0.616 |
| GGGTCGTCCC | 1.000 | 0.817 | 0.877 | 0.775 | GGGCTTTCAA | -0.301 | 0.762 | 0.650 | 0.596 |
| GTGATTACCC | 1.000 | 0.793 | 0.798 | 0.685 | GGTACGCCCC | -0.301 | 0.783 | 0.823 | 0.645 |
| AGGGGTTCTC | 1.000 | 0.807 | 0.657 | 0.652 | GGCCGTTCCC | -0.301 | 0.782 | 0.691 | 0.697 |
| GGTGAGTCCC | 1.000 | 0.877 | 0.864 | 0.807 | GGTGTATCCT | -0.301 | 0.782 | 0.665 | 0.609 |
| GGGGGGGTCC | 1.000 | 0.803 | 0.875 | 0.755 | CTGACTTTCC | -0.301 | 0.749 | 0.687 | 0.502 |
| AGGGTATTCC | 1.000 | 0.781 | 0.755 | 0.638 | GCGATTTCCC | -0.301 | 0.872 | 0.831 | 0.713 |
| GGGTTACCCC | 1.000 | 0.751 | 0.808 | 0.751 | GGGATTCTAA | -0.301 | 0.750 | 0.703 | 0.521 |
| GGGTGGTCCC | 1.000 | 0.827 | 0.809 | 0.770 | GAGATTTTCT | -0.301 | 0.756 | 0.704 | 0.529 |
| GGGGCTGTAC | 1.000 | 0.835 | 0.827 | 0.687 | TGTACTGCCC | -0.301 | 0.801 | 0.700 | 0.554 |
| GTGGGATTCC | 1.000 | 0.770 | 0.688 | 0.633 | AGGAGCTTCC | -0.301 | 0.760 | 0.669 | 0.556 |
| GGGATTTCTA | 1.000 | 0.837 | 0.723 | 0.607 | TGAGGTTCAC | -0.301 | 0.772 | 0.530 | 0.519 |
| GGGGATTATC | 1.000 | 0.800 | 0.781 | 0.779 | GGGAGTATCT | -0.301 | 0.758 | 0.730 | 0.629 |
| GGTGGTCTCC | 1.000 | 0.846 | 0.764 | 0.699 | GGTCATCTCC | -0.301 | 0.758 | 0.761 | 0.693 |
| TGGGATTTTC | 0.997 | 0.792 | 0.727 | 0.657 | GGGGATGCAT | -0.301 | 0.772 | 0.758 | 0.735 |
| GGTTGTTTCC | 0.993 | 0.868 | 0.672 | 0.619 | CGGGCGTCCC | -0.301 | 0.779 | 0.843 | 0.730 |
| GGGGATCTTC | 0.989 | 0.798 | 0.854 | 0.781 | GGTGCATTAC | -0.301 | 0.795 | 0.630 | 0.505 |
| GGTGATTTTT | 0.989 | 0.763 | 0.619 | 0.551 | CTTAGTTCCC | -0.301 | 0.763 | 0.511 | 0.442 |
| GGGGGTGTAC | 0.985 | 0.845 | 0.758 | 0.682 | TGTACTTACC | -0.301 | 0.753 | 0.614 | 0.491 |
| GGGGTTTCTT | 0.985 | 0.813 | 0.742 | 0.684 | GATACTTCCC | -0.301 | 0.846 | 0.707 | 0.580 |
| GGTTTTTCAA | 0.982 | 0.767 | 0.523 | 0.463 | GGGGGTATCA | -0.301 | 0.763 | 0.750 | 0.706 |
| AGGGTCTTCC | 0.978 | 0.759 | 0.755 | 0.638 | GTAGTTTTAC | -0.301 | 0.747 | 0.578 | 0.447 |
| AGGTGTTCCC | 0.978 | 0.814 | 0.691 | 0.697 | GGGATTACTC | -0.301 | 0.822 | 0.798 | 0.685 |
| GGGGCAGTAC | 0.978 | 0.752 | 0.750 | 0.613 | GGTGCTTTAA | -0.301 | 0.789 | 0.598 | 0.474 |
| GGGAATTCTC | 0.978 | 0.923 | 0.854 | 0.790 | GATATTCTCC | -0.301 | 0.750 | 0.685 | 0.494 |
| GGGGACTTAC | 0.978 | 0.810 | 0.777 | 0.715 | CGTAATTTGC | -0.301 | 0.750 | 0.581 | 0.446 |
| AGGTATTTCC | 0.978 | 0.794 | 0.762 | 0.701 | TGAAATTTAC | -0.301 | 0.761 | 0.581 | 0.446 |
| GGGGATTGTC | 0.978 | 0.779 | 0.781 | 0.779 | GTTAGTGCCC | -0.301 | 0.803 | 0.631 | 0.549 |
| GGTGGAACCC | 0.978 | 0.773 | 0.674 | 0.682 | CGTATATCCC | -0.301 | 0.791 | 0.628 | 0.505 |
| GGATCTTCCC | 0.978 | 0.837 | 0.760 | 0.702 | GGAACTTTAG | -0.301 | 0.752 | 0.660 | 0.403 |
| GGTCATTCCC | 0.978 | 0.873 | 0.781 | 0.779 | GCAGGTTTCC | -0.301 | 0.760 | 0.638 | 0.575 |
| GTGGTTTTAC | 0.970 | 0.815 | 0.705 | 0.580 | GGCGGTTCCC | -0.301 | 0.875 | 0.784 | 0.785 |
| GTTTTTTCAC | 0.969 | 0.753 | 0.505 | 0.436 | GGGGATGCTA | -0.301 | 0.759 | 0.758 | 0.735 |
| GTGATTTCCG | 0.962 | 0.808 | 0.804 | 0.613 | GGGCGATTCC | -0.301 | 0.780 | 0.722 | 0.677 |
| GGTGTTTTTG | 0.962 | 0.780 | 0.677 | 0.480 | AGGAGTTTTC | -0.301 | 0.791 | 0.619 | 0.497 |
| GGTTTTGTAC | 0.961 | 0.765 | 0.606 | 0.466 | GGAGCTTCAC | -0.301 | 0.857 | 0.726 | 0.657 |
| GGGGTTCTAC | 0.954 | 0.829 | 0.831 | 0.704 | GGGATGGCTC | -0.301 | 0.773 | 0.815 | 0.627 |
| GGGGGTCTAC | 0.954 | 0.820 | 0.764 | 0.699 | GATATTTCGC | -0.301 | 0.770 | 0.578 | 0.447 |
| TGGGGTATCC | 0.954 | 0.757 | 0.731 | 0.679 | TGGGCATCAC | -0.301 | 0.747 | 0.649 | 0.582 |
| TGTATTCCCC | 0.954 | 0.795 | 0.704 | 0.571 | GGGAGGTCTC | -0.301 | 0.829 | 0.755 | 0.648 |
| GGGGACCCAC | 0.954 | 0.747 | 0.796 | 0.784 | GTTAATACCC | -0.301 | 0.769 | 0.694 | 0.628 |
| GGGGTACCCC | 0.954 | 0.818 | 0.900 | 0.840 | CGGATTGCTC | -0.301 | 0.757 | 0.698 | 0.554 |
| GGGGTCTACC | 0.954 | 0.773 | 0.808 | 0.760 | GTTAGTGCAC | -0.301 | 0.756 | 0.505 | 0.416 |
| GGGCTATCCC | 0.954 | 0.814 | 0.808 | 0.760 | TGTACTCTCC | -0.301 | 0.751 | 0.687 | 0.494 |
| GGGAGTCCCG | 0.954 | 0.813 | 0.864 | 0.732 | GGTACTCCCT | -0.301 | 0.768 | 0.725 | 0.598 |
| GGGGTATTAC | 0.954 | 0.835 | 0.755 | 0.638 | AGGATTTTGC | -0.301 | 0.779 | 0.686 | 0.502 |
| GGGGATTCTG | 0.950 | 0.824 | 0.846 | 0.767 | AGTGAGTTCC | -0.301 | 0.751 | 0.718 | 0.597 |
| GTAGTTTTCC | 0.943 | 0.794 | 0.705 | 0.580 | GGAGATGTCC | -0.301 | 0.828 | 0.848 | 0.764 |
| GGGGGTATTC | 0.942 | 0.778 | 0.731 | 0.679 | TGGGGATCAC | -0.301 | 0.757 | 0.580 | 0.577 |
| GGGATGTTCC | 0.938 | 0.886 | 0.929 | 0.709 | GGTAATGCTC | -0.301 | 0.837 | 0.721 | 0.631 |
| GGGGTTGTAC | 0.938 | 0.853 | 0.825 | 0.687 | GGGTATCCTC | -0.301 | 0.757 | 0.780 | 0.770 |
| GGTATTCTCC | 0.938 | 0.864 | 0.812 | 0.627 | ATGACTTTCC | -0.301 | 0.753 | 0.687 | 0.502 |
| GGGGTTTTGA | 0.933 | 0.781 | 0.723 | 0.607 | GTGGATATCC | -0.301 | 0.754 | 0.821 | 0.761 |
| GGGAGTACCC | 0.929 | 0.887 | 0.858 | 0.812 | GGAAGTTCTG | -0.301 | 0.760 | 0.611 | 0.475 |
| CGGGCATTCC | 0.929 | 0.758 | 0.757 | 0.638 | TGGAATTCTC | -0.301 | 0.828 | 0.727 | 0.657 |
| GGGTGATCCC | 0.929 | 0.832 | 0.741 | 0.755 | GGAATTTAAC | -0.301 | 0.773 | 0.612 | 0.491 |
| AGGGGTTCCC | 0.929 | 0.881 | 0.784 | 0.785 | TGGACTTGCC | -0.301 | 0.753 | 0.741 | 0.624 |
| GGGTATGCCC | 0.929 | 0.855 | 0.901 | 0.886 | GGGGCGTCAC | -0.301 | 0.837 | 0.843 | 0.730 |
| GGGGACTGCC | 0.929 | 0.749 | 0.831 | 0.837 | CGGGTTTCCA | -0.301 | 0.797 | 0.742 | 0.684 |
| GGAATTTCCT | 0.929 | 0.829 | 0.723 | 0.607 | GAGCGTTTCC | -0.301 | 0.749 | 0.672 | 0.619 |
| GGATTTGCCC | 0.929 | 0.791 | 0.752 | 0.676 | TAGACTTTCC | -0.301 | 0.747 | 0.687 | 0.502 |
| GGGATTTGCC | 0.929 | 0.867 | 0.866 | 0.757 | GGAACTGCTC | -0.301 | 0.776 | 0.700 | 0.554 |
| GGGGGCATCC | 0.929 | 0.747 | 0.781 | 0.738 | GTGGCTTACC | -0.301 | 0.756 | 0.760 | 0.702 |
| GGGGCTATCC | 0.929 | 0.842 | 0.927 | 0.817 | GGGACTGCTA | -0.301 | 0.754 | 0.718 | 0.581 |
| GGATTTTTTC | 0.923 | 0.756 | 0.612 | 0.491 | GGTAATTGGC | -0.301 | 0.747 | 0.635 | 0.568 |
| GGGAATTTCG | 0.921 | 0.882 | 0.934 | 0.745 | GGTCTTACCC | -0.301 | 0.772 | 0.725 | 0.673 |
| AGGTTTTTCC | 0.919 | 0.797 | 0.739 | 0.624 | GGGGCTTAAC | -0.301 | 0.813 | 0.760 | 0.702 |
| GTTTTTTTCC | 0.919 | 0.774 | 0.612 | 0.491 | CGGAATTTTC | -0.301 | 0.793 | 0.708 | 0.579 |
| GGTTTGTCCC | 0.918 | 0.814 | 0.749 | 0.642 | GGCAGTTTCC | -0.301 | 0.859 | 0.746 | 0.630 |
| GGATTTTTAC | 0.916 | 0.783 | 0.612 | 0.491 | TGTGATTACC | -0.301 | 0.758 | 0.654 | 0.646 |
| GGTGGTTTCT | 0.915 | 0.831 | 0.656 | 0.602 | GGGGTGTCAA | -0.301 | 0.766 | 0.732 | 0.624 |
| GGCTTTTTCC | 0.915 | 0.791 | 0.739 | 0.624 | GGCGTATTCC | -0.301 | 0.774 | 0.755 | 0.638 |
| GGTGTTGTCC | 0.910 | 0.879 | 0.825 | 0.687 | AAGATTTTCC | -0.301 | 0.759 | 0.686 | 0.502 |
| CGGGTTTTTC | 0.908 | 0.786 | 0.705 | 0.580 | CGGGCTTCCG | -0.301 | 0.778 | 0.825 | 0.690 |
| GGTTTTGTCC | 0.907 | 0.812 | 0.732 | 0.599 | TGAATTTTCC | -0.301 | 0.811 | 0.686 | 0.502 |
| AGGGGTTTAC | 0.903 | 0.809 | 0.638 | 0.575 | CGTAGTTTCA | -0.301 | 0.751 | 0.510 | 0.391 |
| GGTACAGTCC | 0.903 | 0.787 | 0.731 | 0.535 | GGCAGTTCCT | -0.301 | 0.781 | 0.656 | 0.602 |
| AGGGGTATCC | 0.903 | 0.751 | 0.731 | 0.679 | GGGACGTCCG | -0.301 | 0.804 | 0.923 | 0.686 |
| GGTAATCTCC | 0.903 | 0.861 | 0.835 | 0.704 | GGTATTCTAC | -0.301 | 0.818 | 0.685 | 0.494 |
| GGTGACTCCC | 0.903 | 0.861 | 0.796 | 0.792 | CGGACTTTCG | -0.301 | 0.762 | 0.787 | 0.536 |
| AGGGGTTTCT | 0.903 | 0.752 | 0.656 | 0.602 | GGTAAGTTCG | -0.301 | 0.772 | 0.798 | 0.552 |
| GGAGACTCCC | 0.903 | 0.814 | 0.796 | 0.792 | GGGATGTTCA | -0.301 | 0.797 | 0.821 | 0.602 |
| GTGACTCCCC | 0.903 | 0.790 | 0.833 | 0.704 | CGGACTTCTC | -0.301 | 0.804 | 0.707 | 0.580 |
| GGGGGTTGCC | 0.903 | 0.848 | 0.818 | 0.830 | GGTGTTCGCC | -0.301 | 0.747 | 0.758 | 0.693 |
| GTGAGTCCCC | 0.903 | 0.800 | 0.764 | 0.699 | GCTATATCCC | -0.301 | 0.768 | 0.628 | 0.505 |
| GGTATGCCCC | 0.903 | 0.802 | 0.821 | 0.645 | GGGGACTCCG | -0.301 | 0.793 | 0.896 | 0.825 |
| GGGGCTGCAC | 0.903 | 0.861 | 0.846 | 0.765 | ATGGATTTCC | -0.301 | 0.758 | 0.727 | 0.657 |
| AGGACTTTCC | 0.903 | 0.856 | 0.814 | 0.635 | AGGGGTTTCG | -0.301 | 0.766 | 0.737 | 0.608 |
| GGTTTTGCTC | 0.894 | 0.764 | 0.625 | 0.543 | GGTACATTCT | -0.301 | 0.748 | 0.629 | 0.454 |
| AGTGTTTTCC | 0.891 | 0.843 | 0.705 | 0.580 | GGTGGTGCAA | -0.301 | 0.760 | 0.542 | 0.520 |
| GGGTTTTCAT | 0.888 | 0.773 | 0.650 | 0.596 | GGGGGTCTCG | -0.301 | 0.777 | 0.864 | 0.732 |
| GGTGTTGCCC | 0.885 | 0.904 | 0.844 | 0.765 | GTCAATTTCC | -0.301 | 0.761 | 0.708 | 0.579 |
| CGGGTGTTCC | 0.885 | 0.772 | 0.822 | 0.653 | GGGGCTTTAA | -0.301 | 0.811 | 0.725 | 0.607 |
| GGTCCTTTCC | 0.885 | 0.833 | 0.741 | 0.624 | AGGGGTGCAC | -0.301 | 0.770 | 0.650 | 0.627 |
| AGTGGTTTCC | 0.881 | 0.834 | 0.638 | 0.575 | CGGGGTACCC | -0.301 | 0.773 | 0.750 | 0.757 |
| GGTATTTCCG | 0.879 | 0.890 | 0.804 | 0.613 | TGTATCTCCC | -0.301 | 0.779 | 0.628 | 0.505 |
| GTGAATACCC | 0.875 | 0.790 | 0.821 | 0.761 | CGGAGTTTAC | -0.301 | 0.815 | 0.619 | 0.497 |
| GGGGGATTGC | 0.875 | 0.778 | 0.688 | 0.633 | GGGACTATTC | -0.301 | 0.778 | 0.781 | 0.607 |
| GGGAGCACCC | 0.875 | 0.783 | 0.781 | 0.738 | GGGACCTTCG | -0.301 | 0.762 | 0.837 | 0.594 |
| TGGAATTTCC | 0.875 | 0.876 | 0.835 | 0.712 | GGGTACGCCC | -0.301 | 0.751 | 0.824 | 0.811 |
| GGTGAATACC | 0.875 | 0.770 | 0.704 | 0.704 | GGGAGTCTAC | -0.301 | 0.830 | 0.745 | 0.622 |
| TGGGATCTCC | 0.875 | 0.777 | 0.854 | 0.781 | GCGAAGTCCC | -0.301 | 0.780 | 0.844 | 0.730 |
| GGGAGACTCC | 0.875 | 0.793 | 0.795 | 0.680 | GGGGTTACCA | -0.301 | 0.797 | 0.836 | 0.789 |
| GGGGAATCCT | 0.875 | 0.800 | 0.815 | 0.819 | GGTGATGGCC | -0.301 | 0.768 | 0.774 | 0.753 |
| GGGGATGTTC | 0.875 | 0.823 | 0.848 | 0.764 | TGCGATTCCC | -0.301 | 0.785 | 0.746 | 0.734 |
| GGGAAATTTC | 0.875 | 0.814 | 0.758 | 0.637 | GAGATGTCCC | -0.301 | 0.797 | 0.822 | 0.653 |
| AGGGGCTCCC | 0.875 | 0.776 | 0.707 | 0.710 | GTGAATTTTC | -0.301 | 0.794 | 0.708 | 0.579 |
| GGGGTTTCTA | 0.875 | 0.827 | 0.742 | 0.684 | TGGTATTCAC | -0.301 | 0.778 | 0.654 | 0.646 |
| GGGAGTTCAC | 0.875 | 0.945 | 0.765 | 0.708 | GCGTATTCCC | -0.301 | 0.792 | 0.781 | 0.779 |
| GGGATTCTAC | 0.875 | 0.839 | 0.812 | 0.627 | GGGACTTGTC | -0.301 | 0.775 | 0.741 | 0.624 |
| GGGGCTTTCA | 0.875 | 0.857 | 0.852 | 0.740 | GGGCCTTTGC | -0.301 | 0.759 | 0.741 | 0.624 |
| GGGTTTTCAG | 0.875 | 0.788 | 0.731 | 0.602 | TGTATTCTCC | -0.301 | 0.769 | 0.685 | 0.494 |
| GGGGGTTTCG | 0.874 | 0.866 | 0.864 | 0.741 | GGGATAGTTC | -0.301 | 0.753 | 0.729 | 0.535 |
| TGGTTTTTCC | 0.874 | 0.803 | 0.739 | 0.624 | CGGAGTGTCC | -0.301 | 0.797 | 0.739 | 0.605 |
| GGGCTTTCTC | 0.872 | 0.824 | 0.758 | 0.702 | GGTGTAATCC | -0.301 | 0.756 | 0.721 | 0.610 |
| GTGGTTTTCT | 0.869 | 0.758 | 0.723 | 0.607 | CGGAGTTCTC | -0.301 | 0.813 | 0.638 | 0.575 |
| GGAATTTTTC | 0.869 | 0.833 | 0.686 | 0.502 | GGTAGGGCCC | -0.301 | 0.817 | 0.748 | 0.622 |
| GGAGTTTCCG | 0.865 | 0.833 | 0.823 | 0.690 | GGTCCTCCCC | -0.301 | 0.769 | 0.760 | 0.693 |
| CGGGTCTTCC | 0.865 | 0.755 | 0.755 | 0.638 | TGTGATTCCT | -0.301 | 0.767 | 0.638 | 0.628 |
| GGGGTTTGCC | 0.864 | 0.857 | 0.885 | 0.835 | GGTAATTTGG | -0.301 | 0.766 | 0.681 | 0.479 |
| CGGTGTTCCC | 0.860 | 0.810 | 0.691 | 0.697 | GGGAATGCGC | -0.301 | 0.837 | 0.848 | 0.764 |
| GGGGCATTTC | 0.860 | 0.789 | 0.757 | 0.638 | GCGAATTTCC | -0.301 | 0.843 | 0.835 | 0.712 |
| GGGGTTTACT | 0.859 | 0.774 | 0.776 | 0.729 | GGCTCTTCCC | -0.301 | 0.799 | 0.760 | 0.702 |
| GGTGGCTTCC | 0.857 | 0.830 | 0.688 | 0.633 | GGTCGGTCCC | -0.301 | 0.780 | 0.682 | 0.637 |
| GGGGTTTCTG | 0.855 | 0.827 | 0.823 | 0.690 | CGGATTTCAG | -0.301 | 0.760 | 0.677 | 0.480 |
| CGGTTTTTCC | 0.854 | 0.793 | 0.739 | 0.624 | GGGAAATTCG | -0.301 | 0.798 | 0.858 | 0.670 |
| GGGATTCTCT | 0.854 | 0.782 | 0.830 | 0.654 | GGGGTGTCCA | -0.301 | 0.813 | 0.859 | 0.757 |
| GGTGGATTTC | 0.851 | 0.778 | 0.561 | 0.500 | GGGGGATCAT | -0.301 | 0.748 | 0.599 | 0.604 |
| GGGGTATCTC | 0.845 | 0.833 | 0.774 | 0.715 | GGAACTTTAC | -0.301 | 0.842 | 0.687 | 0.502 |
| CGTACTTCCC | 0.845 | 0.856 | 0.707 | 0.580 | GGGCCTTTCG | -0.301 | 0.764 | 0.840 | 0.657 |
| GGTGGCTCCC | 0.845 | 0.856 | 0.707 | 0.710 | GACGATTCCC | -0.301 | 0.766 | 0.746 | 0.734 |
| TGGGCATTCC | 0.845 | 0.768 | 0.757 | 0.638 | TGGACTCTCC | -0.301 | 0.772 | 0.814 | 0.627 |
| GGGCTTGCCC | 0.845 | 0.833 | 0.878 | 0.809 | GGGATTTAAC | -0.301 | 0.841 | 0.739 | 0.624 |
| TGGGACTCCC | 0.845 | 0.787 | 0.796 | 0.792 | GAGAGTTCCT | -0.301 | 0.774 | 0.656 | 0.602 |
| GGGAAGTCCT | 0.845 | 0.805 | 0.863 | 0.757 | GGGAACTTCG | -0.301 | 0.777 | 0.858 | 0.670 |
| GGCGTTCCCC | 0.845 | 0.795 | 0.850 | 0.782 | CGGGCTCTCC | -0.301 | 0.753 | 0.833 | 0.704 |
| CGGGGTATCC | 0.845 | 0.747 | 0.731 | 0.679 | GTGGTTCCAC | -0.301 | 0.752 | 0.723 | 0.649 |
| GGGGGTCCAC | 0.845 | 0.846 | 0.783 | 0.777 | GGTATTTACT | -0.301 | 0.763 | 0.631 | 0.518 |
| GGTTAGTCCC | 0.845 | 0.811 | 0.771 | 0.719 | TGGACTGCTC | -0.301 | 0.748 | 0.700 | 0.554 |
| GGGACTCTAC | 0.845 | 0.821 | 0.814 | 0.627 | GGCGACTTCC | -0.301 | 0.750 | 0.777 | 0.715 |
| GTGGCTCCCC | 0.845 | 0.780 | 0.852 | 0.782 | GAGATTTCCG | -0.301 | 0.796 | 0.804 | 0.613 |
| GGTGTAGTCC | 0.845 | 0.795 | 0.748 | 0.613 | GGGGTCTCCA | -0.301 | 0.796 | 0.792 | 0.742 |
| GGGATTCCAC | 0.845 | 0.865 | 0.831 | 0.704 | TATGATTCCC | -0.301 | 0.756 | 0.620 | 0.601 |
| GGTAAATTCC | 0.845 | 0.866 | 0.758 | 0.637 | GTGATTTCAC | -0.301 | 0.851 | 0.705 | 0.580 |
| GGAATTTTCG | 0.845 | 0.817 | 0.785 | 0.536 | GGGAGCTCCT | -0.301 | 0.783 | 0.706 | 0.660 |
| GGACGTTCCC | 0.845 | 0.821 | 0.691 | 0.697 | GGTACGGCCC | -0.301 | 0.808 | 0.817 | 0.627 |
| GGGAGTGCCC | 0.845 | 0.927 | 0.885 | 0.815 | GGTAATTCCG | -0.301 | 0.886 | 0.827 | 0.690 |
| GGGATTACCT | 0.845 | 0.793 | 0.816 | 0.711 | GGTACTACCT | -0.301 | 0.753 | 0.692 | 0.578 |
| GGGATTGTAC | 0.845 | 0.863 | 0.806 | 0.610 | CGGGTTTCGC | -0.301 | 0.791 | 0.724 | 0.657 |
| GGGATTCTTC | 0.836 | 0.812 | 0.812 | 0.627 | GGTATAGCAC | -0.301 | 0.784 | 0.621 | 0.480 |
| CGGTTTTCCC | 0.833 | 0.819 | 0.758 | 0.702 | AGGATTATCC | -0.301 | 0.770 | 0.779 | 0.607 |
| GTGGTTTTCG | 0.829 | 0.772 | 0.804 | 0.613 | GGATATTCCT | -0.301 | 0.749 | 0.672 | 0.672 |
| GTGGTTTTCA | 0.829 | 0.772 | 0.723 | 0.607 | TGGGTAGCCC | -0.301 | 0.747 | 0.767 | 0.690 |
| GGGGGTTCTT | 0.825 | 0.804 | 0.675 | 0.679 | GGGACCTTCT | -0.301 | 0.748 | 0.756 | 0.587 |
| CGGATTTTCC | 0.824 | 0.870 | 0.812 | 0.635 | TGGGCGTCCC | -0.301 | 0.789 | 0.843 | 0.730 |
| GGGACTTTAT | 0.824 | 0.806 | 0.706 | 0.529 | GGATGTTCAC | -0.301 | 0.800 | 0.565 | 0.564 |
| GGGTTTATCC | 0.824 | 0.794 | 0.832 | 0.729 | CGGAGTTTCA | -0.301 | 0.772 | 0.637 | 0.524 |
| GGAATTTTGC | 0.824 | 0.812 | 0.686 | 0.502 | GGGAGTTCAG | -0.301 | 0.856 | 0.737 | 0.608 |
| GGAATTTTCT | 0.820 | 0.803 | 0.704 | 0.529 | TGTGATTCCA | -0.301 | 0.781 | 0.638 | 0.628 |
| AGGGCTTTTC | 0.820 | 0.772 | 0.707 | 0.580 | TGCAATTCCC | -0.301 | 0.795 | 0.727 | 0.657 |
| GGGAGTTTTG | 0.816 | 0.803 | 0.718 | 0.530 | GCTATTTCAC | -0.301 | 0.804 | 0.578 | 0.447 |
| GTTATTTACC | 0.813 | 0.763 | 0.612 | 0.491 | GGGAGTGCCT | -0.301 | 0.823 | 0.776 | 0.709 |
| GGGACGTTCC | 0.813 | 0.868 | 0.931 | 0.709 | TGTAAGTTCC | -0.301 | 0.766 | 0.698 | 0.519 |
| GGTGGAGCCC | 0.813 | 0.813 | 0.700 | 0.685 | GCGGGTTCAC | -0.301 | 0.807 | 0.657 | 0.652 |
| GGTACTCCAC | 0.813 | 0.825 | 0.706 | 0.571 | GCGATTTTCA | -0.301 | 0.757 | 0.704 | 0.529 |
| GGGAATTCTT | 0.813 | 0.819 | 0.746 | 0.683 | TGTTGTTTCC | -0.301 | 0.773 | 0.545 | 0.486 |
| GGTACGTCCC | 0.813 | 0.872 | 0.824 | 0.653 | GCGGATGCCC | -0.301 | 0.794 | 0.867 | 0.842 |
| GGACTTTTAC | 0.813 | 0.757 | 0.612 | 0.491 | GGGAGTGCTC | -0.301 | 0.853 | 0.758 | 0.682 |
| GGGATTCCAT | 0.813 | 0.761 | 0.723 | 0.598 | GGTATTACCA | -0.301 | 0.786 | 0.690 | 0.578 |
| GGGAGTCCAC | 0.813 | 0.856 | 0.764 | 0.699 | GGTTATTGCC | -0.301 | 0.765 | 0.688 | 0.690 |
| GGGACCTTCC | 0.813 | 0.851 | 0.864 | 0.694 | GCGGTTACCC | -0.301 | 0.758 | 0.817 | 0.762 |
| GGGGGTGCAC | 0.813 | 0.870 | 0.777 | 0.760 | CGTGATTCGC | -0.301 | 0.766 | 0.620 | 0.601 |
| TGGAGTCCCC | 0.813 | 0.807 | 0.764 | 0.699 | GGGACTTATC | -0.301 | 0.795 | 0.741 | 0.624 |
| GGTAGTTACC | 0.813 | 0.858 | 0.672 | 0.619 | GGCGATTTCC | -0.301 | 0.854 | 0.854 | 0.790 |
| GGGGGATTCA | 0.813 | 0.783 | 0.706 | 0.660 | GAGGATTTCG | -0.301 | 0.757 | 0.827 | 0.690 |
| GGTATATCCC | 0.813 | 0.896 | 0.755 | 0.638 | GGGGTCGTAC | -0.301 | 0.748 | 0.748 | 0.613 |
| CGGAATTTCC | 0.813 | 0.866 | 0.835 | 0.712 | GATGCTTCCG | -0.301 | 0.747 | 0.698 | 0.557 |
| GGACCTTCCC | 0.813 | 0.812 | 0.760 | 0.702 | GGCGTTGCCC | -0.301 | 0.819 | 0.844 | 0.765 |
| GGGATCTCCG | 0.813 | 0.806 | 0.854 | 0.671 | GGGTATTCCA | -0.301 | 0.831 | 0.799 | 0.805 |
| CGTGCTTCCC | 0.813 | 0.846 | 0.726 | 0.657 | TGTGCCTCCC | -0.301 | 0.751 | 0.649 | 0.582 |
| GTGGTATCCC | 0.813 | 0.804 | 0.774 | 0.715 | GGGCCATCCC | -0.301 | 0.796 | 0.810 | 0.760 |
| CGGGATGTCC | 0.813 | 0.792 | 0.848 | 0.764 | TGTAATTCGC | -0.301 | 0.786 | 0.601 | 0.524 |
| TGGAGTTCCC | 0.813 | 0.896 | 0.765 | 0.708 | GGGTCGTTCC | -0.301 | 0.791 | 0.858 | 0.698 |
| GGGAATTTGT | 0.809 | 0.773 | 0.726 | 0.606 | GTGAATGTCC | -0.301 | 0.803 | 0.828 | 0.687 |
| GAGGGTTTTC | 0.809 | 0.768 | 0.638 | 0.575 | GGTACTTCAT | -0.301 | 0.811 | 0.598 | 0.474 |
| GGTGATTTTA | 0.805 | 0.777 | 0.619 | 0.551 | AGGGTTTTCG | -0.301 | 0.775 | 0.804 | 0.613 |
| GGGTTCTTCC | 0.803 | 0.793 | 0.789 | 0.683 | TGTAACTCCC | -0.301 | 0.776 | 0.650 | 0.582 |
| GGGGGTTTAA | 0.801 | 0.820 | 0.656 | 0.602 | AGGCATTCCC | -0.301 | 0.794 | 0.781 | 0.779 |
| GGGGTCGTCC | 0.801 | 0.795 | 0.875 | 0.746 | GGGGTAGCTC | -0.301 | 0.769 | 0.767 | 0.690 |
| GGTGGCGTCC | 0.796 | 0.765 | 0.681 | 0.607 | GGCACGTCCC | -0.301 | 0.787 | 0.824 | 0.653 |
| GGGGTTTATC | 0.794 | 0.804 | 0.758 | 0.702 | GGAGGTTCAT | -0.301 | 0.764 | 0.549 | 0.546 |
| GGGGTCTTTC | 0.785 | 0.786 | 0.755 | 0.638 | GGGAGGTCCG | -0.301 | 0.814 | 0.854 | 0.681 |
| GGGGCTTTAT | 0.784 | 0.796 | 0.725 | 0.607 | GTTAATGCCC | -0.301 | 0.808 | 0.721 | 0.631 |
| GGAGTTTCCT | 0.778 | 0.819 | 0.742 | 0.684 | GGGACTGCTG | -0.301 | 0.754 | 0.800 | 0.587 |
| GGGGGGCCCC | 0.778 | 0.804 | 0.901 | 0.850 | TTTAATTCCC | -0.301 | 0.778 | 0.601 | 0.524 |
| GGGGACCTCC | 0.778 | 0.767 | 0.904 | 0.839 | GGGTCTATCC | -0.301 | 0.775 | 0.834 | 0.729 |
| GGAGTTTCCA | 0.778 | 0.833 | 0.742 | 0.684 | GGTTGGTCCC | -0.301 | 0.805 | 0.682 | 0.637 |
| GGTAATGCCC | 0.778 | 0.911 | 0.848 | 0.764 | GGGATCTCTC | -0.301 | 0.822 | 0.755 | 0.638 |
| GGTGGATTAC | 0.778 | 0.805 | 0.561 | 0.500 | GGAACCTCCC | -0.301 | 0.809 | 0.757 | 0.638 |
| GGGATCTTTC | 0.778 | 0.796 | 0.735 | 0.561 | AGGGTTTTGC | -0.301 | 0.769 | 0.705 | 0.580 |
| GTGGATTACC | 0.778 | 0.771 | 0.781 | 0.779 | GTGAGTTCAA | -0.301 | 0.753 | 0.529 | 0.469 |
| CGTGATTTCC | 0.778 | 0.835 | 0.727 | 0.657 | GTTAATGTCC | -0.301 | 0.782 | 0.702 | 0.554 |
| GGGATCTCCT | 0.778 | 0.792 | 0.773 | 0.665 | GGTACTATTC | -0.301 | 0.757 | 0.654 | 0.474 |
| GGTGATTCAC | 0.778 | 0.919 | 0.746 | 0.734 | GGTGTATCAC | -0.301 | 0.839 | 0.647 | 0.582 |
| GGGTTTTTAA | 0.778 | 0.762 | 0.631 | 0.518 | GGTACTGTCA | -0.301 | 0.781 | 0.699 | 0.504 |
| GGGACTTCCT | 0.778 | 0.878 | 0.852 | 0.740 | GGCATTGTCC | -0.301 | 0.803 | 0.806 | 0.610 |
| GGGGTAGCCC | 0.778 | 0.842 | 0.894 | 0.823 | CGTAGTGCCC | -0.301 | 0.801 | 0.631 | 0.549 |
| GGGGGCTTAC | 0.778 | 0.804 | 0.688 | 0.633 | GAGGTATCCC | -0.301 | 0.792 | 0.774 | 0.715 |
| GGTATTTCAC | 0.778 | 0.932 | 0.705 | 0.580 | GGGGGTATAC | -0.301 | 0.805 | 0.731 | 0.679 |
| GTTATCTCCC | 0.778 | 0.771 | 0.628 | 0.505 | GGTTCCTCCC | -0.301 | 0.779 | 0.683 | 0.627 |
| GGTTTTACAC | 0.778 | 0.752 | 0.598 | 0.540 | TGGAGCTTCC | -0.301 | 0.766 | 0.669 | 0.556 |
| AGGGGTTGCC | 0.778 | 0.748 | 0.691 | 0.697 | GGGAGGGCTC | -0.301 | 0.765 | 0.748 | 0.622 |
| GGGGTTGCAC | 0.778 | 0.879 | 0.844 | 0.765 | AGAGGTTTCC | -0.301 | 0.787 | 0.638 | 0.575 |
| GGGGTAGTAC | 0.778 | 0.770 | 0.748 | 0.613 | GTGGGCTCCC | -0.301 | 0.774 | 0.707 | 0.710 |
| GTGGTTTTGC | 0.770 | 0.767 | 0.705 | 0.580 | CGGATTTTCT | -0.301 | 0.766 | 0.704 | 0.529 |
| GGGGTGTTTC | 0.766 | 0.802 | 0.822 | 0.653 | GGGAGTTACT | -0.301 | 0.775 | 0.691 | 0.646 |
| GGGGGGTTCT | 0.763 | 0.764 | 0.773 | 0.675 | CGTATTTTCA | -0.301 | 0.760 | 0.577 | 0.396 |
| GGGATTTCAT | 0.760 | 0.850 | 0.723 | 0.607 | GGGACCTTAC | -0.301 | 0.805 | 0.737 | 0.561 |
| GGGGGGTTAC | 0.760 | 0.821 | 0.755 | 0.648 | GGACTTTCAC | -0.301 | 0.783 | 0.631 | 0.569 |
| GGAGTTTTCT | 0.757 | 0.793 | 0.723 | 0.607 | GTGGATTCAC | -0.301 | 0.837 | 0.746 | 0.734 |
| GGGGGGTGCC | 0.754 | 0.760 | 0.809 | 0.770 | GCGAGTTTCC | -0.301 | 0.838 | 0.746 | 0.630 |
| TGGGATTTAC | 0.754 | 0.819 | 0.727 | 0.657 | TGGGATTCAG | -0.301 | 0.756 | 0.719 | 0.634 |
| GGTAGTTTTC | 0.754 | 0.871 | 0.619 | 0.497 | TGGGTTTTCT | -0.301 | 0.766 | 0.723 | 0.607 |
| AGTGCTTTCC | 0.754 | 0.825 | 0.707 | 0.580 | GAGGCTTTAC | -0.301 | 0.785 | 0.707 | 0.580 |
| GGGGATTTGC | 0.751 | 0.866 | 0.854 | 0.790 | TCTAATTCCC | -0.301 | 0.752 | 0.601 | 0.524 |
| CGTGGTTTCC | 0.750 | 0.830 | 0.638 | 0.575 | TGGGGTGCAC | -0.301 | 0.775 | 0.650 | 0.627 |
| GGGATTTCGT | 0.748 | 0.802 | 0.723 | 0.607 | GGCGGTTCAC | -0.301 | 0.828 | 0.657 | 0.652 |
| GTGATTTCCT | 0.748 | 0.794 | 0.723 | 0.607 | GGTGATCCCT | -0.301 | 0.773 | 0.764 | 0.752 |
| GGGGTTTACG | 0.745 | 0.788 | 0.858 | 0.735 | CGGGTTCCCC | -0.301 | 0.797 | 0.850 | 0.782 |
| GGTATTTTTC | 0.744 | 0.879 | 0.686 | 0.502 | TGGATGTCAC | -0.301 | 0.770 | 0.695 | 0.520 |
| GTGCCTTCCC | 0.740 | 0.777 | 0.760 | 0.702 | GGTGCTGCTC | -0.301 | 0.812 | 0.719 | 0.632 |
| GTGGATACCC | 0.740 | 0.780 | 0.840 | 0.839 | AGGACTATCC | -0.301 | 0.751 | 0.781 | 0.607 |
| GGGATGACCC | 0.740 | 0.808 | 0.915 | 0.758 | AGGAGTTGCC | -0.301 | 0.757 | 0.672 | 0.619 |
| GGGATGTCCG | 0.740 | 0.822 | 0.921 | 0.686 | GGGAGTTGCG | -0.301 | 0.769 | 0.772 | 0.652 |
| GTAACTTCCC | 0.740 | 0.811 | 0.707 | 0.580 | GGGAGATTCG | -0.301 | 0.793 | 0.768 | 0.589 |
| GTGAGTTCCC | 0.740 | 0.889 | 0.765 | 0.708 | GATGTTTCCA | -0.301 | 0.766 | 0.615 | 0.551 |
| GGGACTTCTC | 0.740 | 0.908 | 0.833 | 0.713 | GGTGATGCAT | -0.301 | 0.751 | 0.631 | 0.602 |
| GGGATTCCGC | 0.740 | 0.816 | 0.831 | 0.704 | GGGTCTCCAC | -0.301 | 0.770 | 0.760 | 0.693 |
| GGGAGTACCT | 0.740 | 0.784 | 0.750 | 0.706 | TGGGCTATCC | -0.301 | 0.747 | 0.800 | 0.685 |
| GGAATTTTAT | 0.740 | 0.756 | 0.577 | 0.396 | GGTGCTTCAT | -0.301 | 0.801 | 0.617 | 0.551 |
| GGTGGATACC | 0.740 | 0.765 | 0.615 | 0.622 | CGGGCTGTCC | -0.301 | 0.777 | 0.827 | 0.687 |
| GGAGTTTTAC | 0.740 | 0.850 | 0.705 | 0.580 | TGTATTATCC | -0.301 | 0.754 | 0.652 | 0.474 |
| CGGGAGTCCC | 0.740 | 0.794 | 0.864 | 0.807 | GGGATTTGAC | -0.301 | 0.820 | 0.739 | 0.624 |
| GGTATTCCCA | 0.740 | 0.801 | 0.723 | 0.598 | TGGGGTTCAA | -0.301 | 0.751 | 0.549 | 0.546 |
| GGGGATGACC | 0.740 | 0.810 | 0.901 | 0.886 | TGTAATTTCG | -0.301 | 0.765 | 0.681 | 0.479 |
| GGGGGCTTCA | 0.740 | 0.762 | 0.706 | 0.660 | CGGGTCTCCC | -0.301 | 0.781 | 0.774 | 0.715 |
| CGGGGTTTAC | 0.740 | 0.805 | 0.638 | 0.575 | GGAATTTGAC | -0.301 | 0.752 | 0.612 | 0.491 |
| TGGGTATCCC | 0.740 | 0.812 | 0.774 | 0.715 | GGGATTTGCA | -0.301 | 0.777 | 0.757 | 0.651 |
| CGTGGTTCCC | 0.740 | 0.856 | 0.657 | 0.652 | GGTATTCTGC | -0.301 | 0.769 | 0.685 | 0.494 |
| GGAGTTTTTC | 0.739 | 0.823 | 0.705 | 0.580 | GGTGTACCAC | -0.301 | 0.750 | 0.647 | 0.574 |
| GGGACTTCTT | 0.735 | 0.805 | 0.725 | 0.607 | GGGGATGCGC | -0.301 | 0.827 | 0.867 | 0.842 |
| GGTGCTTTCT | 0.733 | 0.822 | 0.725 | 0.607 | GTGGTTGTAC | -0.301 | 0.750 | 0.698 | 0.554 |
| GGTATTGTCC | 0.732 | 0.889 | 0.806 | 0.610 | CGGGCTTTAC | -0.301 | 0.795 | 0.707 | 0.580 |
| GGGGTTATTC | 0.729 | 0.787 | 0.798 | 0.685 | GGGATTGCGC | -0.301 | 0.841 | 0.825 | 0.687 |
| GGACTTTCTC | 0.727 | 0.756 | 0.631 | 0.569 | AGGGTTACCC | -0.301 | 0.786 | 0.817 | 0.762 |
| GGTGTTTTCT | 0.725 | 0.840 | 0.723 | 0.607 | GGAAGTTTGC | -0.301 | 0.803 | 0.619 | 0.497 |
| GGGGCTTCTC | 0.723 | 0.898 | 0.853 | 0.790 | GGTATGTCCG | -0.301 | 0.801 | 0.794 | 0.553 |
| GGGATTCTGC | 0.720 | 0.791 | 0.812 | 0.627 | CGAGCTTTCC | -0.301 | 0.774 | 0.707 | 0.580 |
| GGGATTCTCG | 0.720 | 0.796 | 0.911 | 0.660 | CGGCATTTCC | -0.301 | 0.764 | 0.762 | 0.701 |
| TGGGGTTTAC | 0.719 | 0.814 | 0.638 | 0.575 | GGGGTTACAG | -0.301 | 0.750 | 0.790 | 0.662 |
| GTGTTTTTAC | 0.718 | 0.748 | 0.612 | 0.491 | TGTATTGCAC | -0.301 | 0.773 | 0.571 | 0.421 |
| CGGGGTTCTC | 0.711 | 0.803 | 0.657 | 0.652 | GGTGGTACTC | -0.301 | 0.783 | 0.624 | 0.624 |
| GGGGGTTCTA | 0.711 | 0.819 | 0.675 | 0.679 | CTGGCTTCCC | -0.301 | 0.765 | 0.726 | 0.657 |
| TGGGTTTTTC | 0.708 | 0.795 | 0.705 | 0.580 | AGGAGTTTCC | -0.301 | 0.865 | 0.746 | 0.630 |
| CGGGGTTTCT | 0.704 | 0.748 | 0.656 | 0.602 | TGGGCCTCCC | -0.301 | 0.772 | 0.776 | 0.715 |
| GGGATTCTAG | 0.699 | 0.749 | 0.785 | 0.527 | GAGGATTTCC | -0.301 | 0.847 | 0.854 | 0.790 |
| GGGACTTGCC | 0.699 | 0.848 | 0.868 | 0.757 | GGAGTCTTCC | -0.301 | 0.792 | 0.755 | 0.638 |
| GGGGAGTCCA | 0.699 | 0.809 | 0.882 | 0.834 | TGTAATGTCC | -0.301 | 0.790 | 0.702 | 0.554 |
| GGTATTTCCT | 0.699 | 0.875 | 0.723 | 0.607 | GGCAGATTCC | -0.301 | 0.776 | 0.669 | 0.556 |
| GGGTATACCC | 0.699 | 0.816 | 0.874 | 0.883 | AGTGTTTTCA | -0.301 | 0.754 | 0.596 | 0.474 |
| GAGATTTCCC | 0.699 | 0.886 | 0.831 | 0.713 | GGAGATTCCT | -0.301 | 0.815 | 0.765 | 0.761 |
| GGGGATCCCT | 0.699 | 0.794 | 0.891 | 0.885 | GGAAGTTTCC | -0.301 | 0.898 | 0.746 | 0.630 |
| TGGGGTTCTC | 0.699 | 0.813 | 0.657 | 0.652 | GCTATTTCCC | -0.301 | 0.851 | 0.705 | 0.580 |
| GGTAGTTTAC | 0.699 | 0.898 | 0.619 | 0.497 | GGGCCTTCAC | -0.301 | 0.833 | 0.760 | 0.702 |
| GGTATTTCCA | 0.699 | 0.890 | 0.723 | 0.607 | GTGGATTCAG | -0.301 | 0.748 | 0.719 | 0.634 |
| GGACTTGCCC | 0.699 | 0.765 | 0.752 | 0.676 | GGGGTGTCAC | -0.301 | 0.855 | 0.841 | 0.730 |
| GGGAGTACCG | 0.699 | 0.798 | 0.831 | 0.712 | GGGAACCTCC | -0.301 | 0.777 | 0.884 | 0.762 |
| GGTCTGTCCC | 0.699 | 0.788 | 0.749 | 0.642 | GGTACTTCTA | -0.301 | 0.798 | 0.598 | 0.474 |
| GGTAATTTTC | 0.699 | 0.876 | 0.708 | 0.579 | GTTGCTCCCC | -0.301 | 0.759 | 0.725 | 0.649 |
| GGGGGCCTCC | 0.699 | 0.762 | 0.814 | 0.757 | GGCCCTTCCC | -0.301 | 0.773 | 0.760 | 0.702 |
| CGGGATCTCC | 0.699 | 0.767 | 0.854 | 0.781 | TGTGATGCAC | -0.301 | 0.759 | 0.613 | 0.576 |
| GGATTTTCAC | 0.699 | 0.809 | 0.631 | 0.569 | CGTAAGTTCC | -0.301 | 0.757 | 0.698 | 0.519 |
| GGGGACTCTC | 0.699 | 0.808 | 0.796 | 0.792 | TGAACTTCCC | -0.301 | 0.819 | 0.707 | 0.580 |
| GGTATTGCCC | 0.699 | 0.914 | 0.825 | 0.687 | CGGAGTCTCC | -0.301 | 0.772 | 0.745 | 0.622 |
| GGGGGCCCCC | 0.699 | 0.788 | 0.833 | 0.835 | GGTGATTTAT | -0.301 | 0.790 | 0.619 | 0.551 |
| GGGATTATCC | 0.699 | 0.870 | 0.906 | 0.740 | AGAGTTGCCC | -0.301 | 0.757 | 0.717 | 0.632 |
| CGGGTTATCC | 0.699 | 0.756 | 0.798 | 0.685 | GCGATTGTCC | -0.301 | 0.782 | 0.806 | 0.610 |
| CGGGGCTCCC | 0.699 | 0.772 | 0.707 | 0.710 | GGTGGGGCCC | -0.301 | 0.808 | 0.768 | 0.700 |
| GGTGGATCAC | 0.699 | 0.831 | 0.580 | 0.577 | GGGGGTACCA | -0.301 | 0.788 | 0.769 | 0.784 |
| GGGTGATCTC | 0.699 | 0.758 | 0.615 | 0.622 | GGGTCATCTC | -0.301 | 0.748 | 0.683 | 0.627 |
| GGTGTTCCAC | 0.699 | 0.833 | 0.723 | 0.649 | GTGAAGTTCC | -0.301 | 0.780 | 0.825 | 0.652 |
| GGGATTTCAG | 0.699 | 0.864 | 0.804 | 0.613 | GGCGCTGTCC | -0.301 | 0.775 | 0.827 | 0.687 |
| GGGATTTCGG | 0.699 | 0.816 | 0.804 | 0.613 | GCTGCTTCCC | -0.301 | 0.823 | 0.726 | 0.657 |
| GTGTCTTCCC | 0.699 | 0.802 | 0.760 | 0.702 | GCTAGTTCCG | -0.301 | 0.753 | 0.611 | 0.475 |
| GGCAATTCCC | 0.699 | 0.890 | 0.854 | 0.790 | GGTACGTCTC | -0.301 | 0.799 | 0.697 | 0.520 |
| GGGAATTTAG | 0.699 | 0.835 | 0.808 | 0.612 | CGGACTTCCG | -0.301 | 0.788 | 0.806 | 0.613 |
| GGGAATTTGC | 0.699 | 0.876 | 0.835 | 0.712 | CGGACTTCGC | -0.301 | 0.783 | 0.707 | 0.580 |
| GGGCTTCTCC | 0.699 | 0.783 | 0.865 | 0.749 | GGTGGATTCG | -0.301 | 0.762 | 0.661 | 0.533 |
| TAGATTTCCC | 0.699 | 0.791 | 0.705 | 0.580 | GGGACTTGGC | -0.301 | 0.754 | 0.741 | 0.624 |
| GGGACTTTCG | 0.699 | 0.867 | 0.914 | 0.669 | TGTCGTTCCC | -0.301 | 0.773 | 0.565 | 0.564 |
| GGGACTCCCT | 0.699 | 0.789 | 0.851 | 0.731 | AGGAGTTTGC | -0.301 | 0.770 | 0.619 | 0.497 |
| GGTAGATTTC | 0.699 | 0.788 | 0.542 | 0.423 | CTGAATTTCC | -0.301 | 0.763 | 0.708 | 0.579 |
| GATTTTTTCC | 0.694 | 0.762 | 0.612 | 0.491 | AGGAATTTCT | -0.301 | 0.767 | 0.726 | 0.606 |
| GGTGTTCCCT | 0.692 | 0.777 | 0.742 | 0.676 | GAGTGTTCCC | -0.301 | 0.800 | 0.691 | 0.697 |
| GGGGGTTATC | 0.688 | 0.795 | 0.691 | 0.697 | GGAGGTTTGC | -0.301 | 0.793 | 0.638 | 0.575 |
| GGTGATTTTG | 0.688 | 0.777 | 0.700 | 0.557 | GGGGCTTACA | -0.301 | 0.770 | 0.778 | 0.729 |
| GGGTTTCTAC | 0.681 | 0.762 | 0.739 | 0.616 | CGCGGTTCCC | -0.301 | 0.770 | 0.657 | 0.652 |
| GTGGTTGCCC | 0.681 | 0.823 | 0.844 | 0.765 | TGGGTATCAC | -0.301 | 0.765 | 0.647 | 0.582 |
| TGGTGTTCCC | 0.679 | 0.820 | 0.691 | 0.697 | TGTAGTGCAC | -0.301 | 0.764 | 0.505 | 0.416 |
| GGTTTCTTCC | 0.679 | 0.772 | 0.662 | 0.550 | TGTAGGTCCC | -0.301 | 0.787 | 0.628 | 0.515 |
| AGTGTTTCCC | 0.677 | 0.869 | 0.724 | 0.657 | GTTGATTTCC | -0.301 | 0.837 | 0.727 | 0.657 |
| GCGGGTTTTC | 0.672 | 0.754 | 0.638 | 0.575 | GTGACTTCCA | -0.301 | 0.790 | 0.725 | 0.607 |
| GGGGCTTGCC | 0.669 | 0.838 | 0.887 | 0.835 | TGTAATTCTC | -0.301 | 0.807 | 0.601 | 0.524 |
| GGGTTTTAAC | 0.669 | 0.764 | 0.666 | 0.613 | TGGGCTTCCT | -0.301 | 0.773 | 0.744 | 0.684 |
| GGGTTAGTCC | 0.669 | 0.750 | 0.782 | 0.657 | GCGATGTTCC | -0.301 | 0.758 | 0.803 | 0.576 |
| GGGTTTGTCC | 0.668 | 0.833 | 0.859 | 0.732 | AGCGTTTCCC | -0.301 | 0.783 | 0.724 | 0.657 |
| GGGTGTTTAC | 0.667 | 0.843 | 0.672 | 0.619 | TGGATATCCC | -0.301 | 0.822 | 0.755 | 0.638 |
| GGGGATTTGT | 0.666 | 0.763 | 0.746 | 0.683 | GGTGATTCGA | -0.301 | 0.782 | 0.638 | 0.628 |
| AGGGTTTCTC | 0.653 | 0.816 | 0.724 | 0.657 | GGGGTATCCA | -0.301 | 0.818 | 0.792 | 0.742 |
| GGTGATATCC | 0.653 | 0.836 | 0.821 | 0.761 | GGTAATTATC | -0.301 | 0.789 | 0.635 | 0.568 |
| GGTCGTCCCC | 0.653 | 0.779 | 0.691 | 0.688 | GGTAGTTGAC | -0.301 | 0.790 | 0.545 | 0.486 |
| TGGGATCCCC | 0.653 | 0.803 | 0.873 | 0.859 | GGGATGCTCC | -0.301 | 0.797 | 0.929 | 0.700 |
| GGAATTTCTG | 0.653 | 0.769 | 0.677 | 0.480 | GCGGTTTCCG | -0.301 | 0.773 | 0.823 | 0.690 |
| GGGATTTTAA | 0.653 | 0.839 | 0.704 | 0.529 | AGGGATTTCG | -0.301 | 0.771 | 0.827 | 0.690 |
| GGGGTTTACA | 0.653 | 0.789 | 0.776 | 0.729 | GGTACTTCGT | -0.301 | 0.763 | 0.598 | 0.474 |
| CGGGATATCC | 0.653 | 0.752 | 0.821 | 0.761 | GGAACTCTCC | -0.301 | 0.799 | 0.814 | 0.627 |
| GGGATTTCGA | 0.653 | 0.816 | 0.723 | 0.607 | GGTAATTTTA | -0.301 | 0.787 | 0.600 | 0.473 |
| GGGGACTTCA | 0.653 | 0.767 | 0.796 | 0.742 | GCAAGTTCCC | -0.301 | 0.796 | 0.638 | 0.575 |
| GGGAGTACCA | 0.653 | 0.798 | 0.750 | 0.706 | GTGAATGTAC | -0.301 | 0.757 | 0.702 | 0.554 |
| GGAATTTCCG | 0.653 | 0.843 | 0.804 | 0.613 | AGGCGTTTCC | -0.301 | 0.763 | 0.672 | 0.619 |
| CGGGGTTTCA | 0.653 | 0.762 | 0.656 | 0.602 | GGGAATTCGA | -0.301 | 0.813 | 0.746 | 0.683 |
| GGGACTTCCG | 0.653 | 0.892 | 0.933 | 0.746 | AGGTTTGCCC | -0.301 | 0.758 | 0.752 | 0.676 |
| GGGCAATTCC | 0.653 | 0.785 | 0.812 | 0.759 | GGTGAGATCC | -0.301 | 0.748 | 0.811 | 0.701 |
| GGGGATCCCG | 0.653 | 0.808 | 0.972 | 0.892 | GGGAATCTGC | -0.301 | 0.787 | 0.835 | 0.704 |
| GATCTTTCCC | 0.653 | 0.762 | 0.631 | 0.569 | GATGATTTCC | -0.301 | 0.825 | 0.727 | 0.657 |
| TGGGAGTCCC | 0.653 | 0.803 | 0.864 | 0.807 | TGGACTTCCA | -0.301 | 0.798 | 0.725 | 0.607 |
| GGTTATTCAC | 0.653 | 0.852 | 0.654 | 0.646 | GGCAAGTTCC | -0.301 | 0.776 | 0.825 | 0.652 |
| GGTACCTCCC | 0.653 | 0.856 | 0.757 | 0.638 | AGTGTTCTCC | -0.301 | 0.754 | 0.704 | 0.571 |
| GGAATTCCGC | 0.653 | 0.748 | 0.704 | 0.571 | AGGTTTTCTC | -0.301 | 0.749 | 0.631 | 0.569 |
| GTAGATTTCC | 0.653 | 0.790 | 0.727 | 0.657 | AGTGGTCCCC | -0.301 | 0.771 | 0.657 | 0.644 |
| GGGAACTTTC | 0.653 | 0.792 | 0.758 | 0.637 | AGGGCTTACC | -0.301 | 0.759 | 0.760 | 0.702 |
| GGTAGTTCAC | 0.653 | 0.924 | 0.638 | 0.575 | GGCACTCTCC | -0.301 | 0.760 | 0.814 | 0.627 |
| GGATTATCCC | 0.653 | 0.772 | 0.681 | 0.627 | TGTGACTCCC | -0.301 | 0.766 | 0.670 | 0.659 |
| AGGGATATCC | 0.653 | 0.756 | 0.821 | 0.761 | GGAAGTCCTC | -0.301 | 0.761 | 0.638 | 0.566 |
| GGGAGTTTCT | 0.649 | 0.862 | 0.764 | 0.657 | GGTTCTGCAC | -0.301 | 0.773 | 0.627 | 0.543 |
| TGGGTCTTCC | 0.648 | 0.764 | 0.755 | 0.638 | GGTACGTCCG | -0.301 | 0.783 | 0.796 | 0.553 |
| GGGGGTGTTC | 0.648 | 0.817 | 0.758 | 0.682 | GGGGCCTCCG | -0.301 | 0.778 | 0.875 | 0.749 |
| ATGGGTTTCC | 0.643 | 0.752 | 0.638 | 0.575 | GGAGGTTCCG | -0.301 | 0.824 | 0.757 | 0.685 |
| AGTTTTTCCC | 0.641 | 0.802 | 0.631 | 0.569 | GGTGCTTCAA | -0.301 | 0.815 | 0.617 | 0.551 |
| GGTGGTATCC | 0.636 | 0.831 | 0.731 | 0.679 | GGTATATTTC | -0.301 | 0.796 | 0.609 | 0.428 |
| GTCATTTCCC | 0.636 | 0.791 | 0.705 | 0.580 | GGTATGGTAC | -0.301 | 0.754 | 0.669 | 0.417 |
| TGGGTGTTCC | 0.636 | 0.781 | 0.822 | 0.653 | GATGCATCCC | -0.301 | 0.753 | 0.649 | 0.582 |
| TGGGATTTCA | 0.636 | 0.777 | 0.746 | 0.683 | GCTGCTGCCC | -0.301 | 0.758 | 0.719 | 0.632 |
| GGGGCTTTCG | 0.635 | 0.857 | 0.933 | 0.746 | GGGAGTTATC | -0.301 | 0.805 | 0.672 | 0.619 |
| GGGGGTCTTC | 0.634 | 0.793 | 0.764 | 0.699 | CGTGACTCCC | -0.301 | 0.756 | 0.670 | 0.659 |
| CTGGGTTTCC | 0.634 | 0.748 | 0.638 | 0.575 | TGTAGGTTCC | -0.301 | 0.761 | 0.609 | 0.438 |
| GGGATGTTTC | 0.633 | 0.812 | 0.803 | 0.576 | GGCACTTTAC | -0.301 | 0.803 | 0.687 | 0.502 |
| GGTGGTTTCA | 0.628 | 0.846 | 0.656 | 0.602 | CGTAGTTACC | -0.301 | 0.753 | 0.545 | 0.486 |
| GGGGGTTCCT | 0.628 | 0.878 | 0.802 | 0.812 | GGAATCTCTC | -0.301 | 0.754 | 0.628 | 0.505 |
| GGTACTTTCT | 0.626 | 0.831 | 0.706 | 0.529 | AGTAGTGCAC | -0.301 | 0.759 | 0.505 | 0.416 |
| GGGGATTTAG | 0.623 | 0.825 | 0.827 | 0.690 | GTAATTTTAC | -0.301 | 0.757 | 0.559 | 0.369 |
| GGAGTTTTCG | 0.621 | 0.807 | 0.804 | 0.613 | GACAGTTCCC | -0.301 | 0.770 | 0.638 | 0.575 |
| GGGTGGTTAC | 0.620 | 0.754 | 0.663 | 0.559 | GTTACTTCTC | -0.301 | 0.784 | 0.580 | 0.447 |
| TGTATTTCCC | 0.616 | 0.884 | 0.705 | 0.580 | GGAGGTATCC | -0.301 | 0.784 | 0.731 | 0.679 |
| GGGGTTTCGC | 0.614 | 0.895 | 0.851 | 0.790 | TGTACGTCCC | -0.301 | 0.777 | 0.697 | 0.520 |
| GTGGGTTTCC | 0.614 | 0.853 | 0.765 | 0.708 | GCTGCTTCAC | -0.301 | 0.776 | 0.599 | 0.524 |
| GGTGAATTTC | 0.602 | 0.783 | 0.650 | 0.582 | GGTAAATTCT | -0.301 | 0.763 | 0.650 | 0.531 |
| GGAAATTTCC | 0.602 | 0.903 | 0.835 | 0.712 | GGGCCTGCCC | -0.301 | 0.815 | 0.880 | 0.809 |
| CGGGTATCCC | 0.602 | 0.802 | 0.774 | 0.715 | GGAGCTTCGC | -0.301 | 0.809 | 0.726 | 0.657 |
| TGGGATTACC | 0.602 | 0.779 | 0.781 | 0.779 | TGTGATTTCG | -0.301 | 0.755 | 0.700 | 0.557 |
| GGGTTATTAC | 0.602 | 0.768 | 0.662 | 0.550 | GTTGAGTCCC | -0.301 | 0.775 | 0.737 | 0.674 |
| GGGGGGCTCC | 0.602 | 0.779 | 0.881 | 0.772 | GGGTCTGCCA | -0.301 | 0.752 | 0.772 | 0.703 |
| GTAATTTTCC | 0.602 | 0.803 | 0.686 | 0.502 | TGAAGTTCTC | -0.301 | 0.755 | 0.511 | 0.442 |
| AGTGATTCCC | 0.602 | 0.865 | 0.746 | 0.734 | GATATCTCCC | -0.301 | 0.760 | 0.628 | 0.505 |
| GGGTGTATCC | 0.602 | 0.785 | 0.766 | 0.724 | GGAGATTCAG | -0.301 | 0.783 | 0.719 | 0.634 |
| GGAATTCCAC | 0.602 | 0.797 | 0.704 | 0.571 | GGAATTTCAT | -0.301 | 0.782 | 0.596 | 0.474 |
| GAGGTTTCCC | 0.602 | 0.876 | 0.851 | 0.790 | GGCTGTTCAC | -0.301 | 0.762 | 0.565 | 0.564 |
| GGGGACTTGC | 0.602 | 0.761 | 0.777 | 0.715 | GGTGATACCG | -0.301 | 0.772 | 0.813 | 0.739 |
| GGGACTCTTC | 0.602 | 0.793 | 0.814 | 0.627 | GGGACCTCGC | -0.301 | 0.782 | 0.757 | 0.638 |
| GGTAACTTCC | 0.602 | 0.845 | 0.758 | 0.637 | AGGGGTGCCC | -0.301 | 0.816 | 0.777 | 0.760 |
| GGGTTGTTCC | 0.602 | 0.809 | 0.856 | 0.698 | GGTATAGTAC | -0.301 | 0.759 | 0.602 | 0.402 |
| GGTAATTACC | 0.602 | 0.863 | 0.762 | 0.701 | GGGGATGTCG | -0.301 | 0.807 | 0.947 | 0.797 |
| TGAATTTCCC | 0.602 | 0.837 | 0.705 | 0.580 | AGGGGTCCCC | -0.301 | 0.792 | 0.783 | 0.777 |
| GGGGCGCTCC | 0.602 | 0.769 | 0.950 | 0.777 | CGGAGATTCC | -0.301 | 0.778 | 0.669 | 0.556 |
| GGGGTTAACC | 0.602 | 0.773 | 0.852 | 0.806 | CGTAGATTCC | -0.301 | 0.757 | 0.542 | 0.423 |
| GGTGCCTTCC | 0.602 | 0.820 | 0.757 | 0.638 | GGTGACGTCC | -0.301 | 0.771 | 0.771 | 0.689 |
| GGATATTTCC | 0.602 | 0.826 | 0.762 | 0.701 | GGTCCTGTCC | -0.301 | 0.768 | 0.734 | 0.599 |
| GGGGAGTTCA | 0.602 | 0.784 | 0.863 | 0.757 | GGTTATGCTC | -0.301 | 0.761 | 0.647 | 0.620 |
| AGGTATTCCC | 0.602 | 0.820 | 0.781 | 0.779 | GGCAATTTCG | -0.301 | 0.775 | 0.808 | 0.612 |
| GAGGGTTTCC | 0.602 | 0.841 | 0.765 | 0.708 | CTGAGTTCCC | -0.301 | 0.784 | 0.638 | 0.575 |
| GGTTATTACC | 0.602 | 0.786 | 0.688 | 0.690 | GGTAGTCACC | -0.301 | 0.769 | 0.672 | 0.611 |
| TGGGATTCAC | 0.602 | 0.845 | 0.746 | 0.734 | CGCAGTTTCC | -0.301 | 0.754 | 0.619 | 0.497 |
| GGTCTTTACC | 0.602 | 0.764 | 0.666 | 0.613 | GGAGCTGCTC | -0.301 | 0.766 | 0.719 | 0.632 |
| AGGATTTCCC | 0.602 | 0.900 | 0.831 | 0.713 | GGTGCGGCCC | -0.301 | 0.798 | 0.836 | 0.705 |
| TGGGGCTCCC | 0.602 | 0.782 | 0.707 | 0.710 | GGGTCTTCCA | -0.301 | 0.816 | 0.778 | 0.729 |
| GGGAGATTTC | 0.602 | 0.809 | 0.669 | 0.556 | GGGTCTTACC | -0.301 | 0.793 | 0.795 | 0.746 |
| GGTGTCTCCC | 0.602 | 0.864 | 0.774 | 0.715 | GGGACGTCGC | -0.301 | 0.799 | 0.824 | 0.653 |
| AGTACTTTCC | 0.602 | 0.834 | 0.687 | 0.502 | AGTGCTGTCC | -0.301 | 0.760 | 0.700 | 0.554 |
| AGGGTTTTAC | 0.602 | 0.817 | 0.705 | 0.580 | CGGATTGTCC | -0.301 | 0.805 | 0.806 | 0.610 |
| GGAAAGTTCC | 0.602 | 0.815 | 0.825 | 0.652 | CGCATTTCCC | -0.301 | 0.789 | 0.705 | 0.580 |
| GGGAACTTCT | 0.602 | 0.763 | 0.776 | 0.664 | CGCATTTTCC | -0.301 | 0.763 | 0.686 | 0.502 |
| GGAATTTCGC | 0.602 | 0.837 | 0.705 | 0.580 | TGTAATATCC | -0.301 | 0.751 | 0.675 | 0.551 |
| GGGTCATTCC | 0.602 | 0.796 | 0.791 | 0.683 | AGTGATTCAC | -0.301 | 0.819 | 0.620 | 0.601 |
| GGTCATTTCC | 0.602 | 0.847 | 0.762 | 0.701 | GGAACTTCTG | -0.301 | 0.751 | 0.679 | 0.480 |
| GGGGGTTGTC | 0.597 | 0.774 | 0.691 | 0.697 | GGTAGTGCCG | -0.301 | 0.816 | 0.731 | 0.582 |
| GGGGGTTTGT | 0.590 | 0.758 | 0.656 | 0.602 | GGGATTTCAA | -0.301 | 0.864 | 0.723 | 0.607 |
| GGGGGCTTCT | 0.588 | 0.748 | 0.706 | 0.660 | TGAGGTTCCC | -0.301 | 0.818 | 0.657 | 0.652 |
| GTGGGGTTCC | 0.583 | 0.765 | 0.755 | 0.648 | GGAGATTTCA | -0.301 | 0.804 | 0.746 | 0.683 |
| GGCGTTTCCC | 0.580 | 0.883 | 0.851 | 0.790 | GGTGATACCC | -0.301 | 0.862 | 0.840 | 0.839 |
| TGTGATTCCC | 0.580 | 0.870 | 0.746 | 0.734 | TGTAGTTACC | -0.301 | 0.763 | 0.545 | 0.486 |
| GGGGGGTTCA | 0.574 | 0.778 | 0.773 | 0.675 | GGGGATCTCA | -0.301 | 0.783 | 0.872 | 0.808 |
| GAGTTTTTCC | 0.568 | 0.783 | 0.739 | 0.624 | GGAAATTCAT | -0.301 | 0.779 | 0.619 | 0.551 |
| CGGGGTGTCC | 0.565 | 0.787 | 0.758 | 0.682 | TCGAGTTCCC | -0.301 | 0.768 | 0.638 | 0.575 |
| GGGTTTTCAA | 0.565 | 0.788 | 0.650 | 0.596 | GGAGTTGTTC | -0.301 | 0.758 | 0.698 | 0.554 |
| GGTTATCTCC | 0.565 | 0.784 | 0.761 | 0.693 | GCGGATTCCG | -0.301 | 0.769 | 0.846 | 0.767 |
| GGGGTGGTCC | 0.565 | 0.811 | 0.942 | 0.760 | AGGATTTTCA | -0.301 | 0.785 | 0.704 | 0.529 |
| GGGATATTTC | 0.565 | 0.817 | 0.735 | 0.561 | GAAATTTTCC | -0.301 | 0.792 | 0.686 | 0.502 |
| GGGATGTCCT | 0.565 | 0.808 | 0.840 | 0.680 | GGTGTGCCCC | -0.301 | 0.792 | 0.841 | 0.722 |
| GGGAATTCTG | 0.565 | 0.834 | 0.827 | 0.690 | GGCGCTGCCC | -0.301 | 0.801 | 0.846 | 0.765 |
| GTGTTTTCAC | 0.565 | 0.774 | 0.631 | 0.569 | GCGGTTTTAC | -0.301 | 0.790 | 0.705 | 0.580 |
| GGGATTATTC | 0.565 | 0.796 | 0.779 | 0.607 | GGGATGCCTC | -0.301 | 0.749 | 0.821 | 0.645 |
| TGTGGTTCCC | 0.565 | 0.865 | 0.657 | 0.652 | GGGGGATTCG | -0.301 | 0.783 | 0.787 | 0.666 |
| GTGGTATTCC | 0.565 | 0.778 | 0.755 | 0.638 | AGTACTTCCT | -0.301 | 0.757 | 0.598 | 0.474 |
| TGGGTTATCC | 0.565 | 0.765 | 0.798 | 0.685 | GTTACTGCCC | -0.301 | 0.793 | 0.700 | 0.554 |
| GGATCTTTCC | 0.565 | 0.812 | 0.741 | 0.624 | GGGCATTCTC | -0.301 | 0.821 | 0.781 | 0.779 |
| GGGGGTCCCT | 0.565 | 0.789 | 0.802 | 0.803 | TGGATTTCAA | -0.301 | 0.769 | 0.596 | 0.474 |
| GGGGGTTTGC | 0.562 | 0.861 | 0.765 | 0.708 | GGCAGTTCCG | -0.301 | 0.795 | 0.737 | 0.608 |
| GAGGTTTTCC | 0.556 | 0.850 | 0.831 | 0.713 | AGTGTTGCCC | -0.301 | 0.804 | 0.717 | 0.632 |
| TGGATTTTCC | 0.556 | 0.879 | 0.812 | 0.635 | GGCGAGTCCC | -0.301 | 0.792 | 0.864 | 0.807 |
| GGTAGTTTTT | 0.554 | 0.767 | 0.510 | 0.391 | GGTGTTGCAA | -0.301 | 0.769 | 0.609 | 0.526 |
| GGGGTTCTTC | 0.554 | 0.802 | 0.831 | 0.704 | GGAACTTCCT | -0.301 | 0.810 | 0.725 | 0.607 |
| GGTATTTTTT | 0.553 | 0.776 | 0.577 | 0.396 | GGGGCCTCAC | -0.301 | 0.821 | 0.776 | 0.715 |
| CCGGGTTCCC | 0.544 | 0.749 | 0.657 | 0.652 | GGTGAATTCG | -0.301 | 0.767 | 0.750 | 0.615 |
| CGGGATTCTC | 0.544 | 0.808 | 0.746 | 0.734 | GTAGGTTCCC | -0.301 | 0.811 | 0.657 | 0.652 |
| CGTGATTCCC | 0.544 | 0.861 | 0.746 | 0.734 | GGTGCTTAAC | -0.301 | 0.792 | 0.633 | 0.569 |
| CGGGGGTCCC | 0.544 | 0.789 | 0.774 | 0.725 | GGGACGTCCT | -0.301 | 0.790 | 0.842 | 0.680 |
| GGGGGGTACC | 0.544 | 0.781 | 0.809 | 0.770 | GGTGTCTACC | -0.301 | 0.752 | 0.681 | 0.627 |
| AGTATTTTCC | 0.544 | 0.853 | 0.686 | 0.502 | GGTTCGTCCC | -0.301 | 0.796 | 0.750 | 0.642 |
| CGGAGTTCCC | 0.544 | 0.887 | 0.765 | 0.708 | GTTAATTCTC | -0.301 | 0.799 | 0.601 | 0.524 |
| GGGAAAGTTC | 0.544 | 0.749 | 0.752 | 0.612 | GGGGATTACG | -0.301 | 0.785 | 0.880 | 0.812 |
| CGTGGATTCC | 0.544 | 0.747 | 0.561 | 0.500 | GGGGCGTTCA | -0.301 | 0.769 | 0.842 | 0.680 |
| GGGACTTAAC | 0.544 | 0.823 | 0.741 | 0.624 | GGGAGCTTCG | -0.301 | 0.772 | 0.768 | 0.589 |
| GTGATATCCC | 0.544 | 0.814 | 0.755 | 0.638 | GGGAGTTCGA | -0.301 | 0.808 | 0.656 | 0.602 |
| GGTGCCGTCC | 0.544 | 0.756 | 0.750 | 0.613 | GGGAGTTAGC | -0.301 | 0.784 | 0.672 | 0.619 |
| GGAGATTTCC | 0.544 | 0.893 | 0.854 | 0.790 | GGTATAGCCC | -0.301 | 0.831 | 0.748 | 0.613 |
| GGTGGTGCAC | 0.544 | 0.849 | 0.650 | 0.627 | GGTAATTTGT | -0.301 | 0.752 | 0.600 | 0.473 |
| GGGCGCTCCC | 0.544 | 0.784 | 0.741 | 0.755 | GGTGCTCTAC | -0.301 | 0.790 | 0.706 | 0.571 |
| GGTACCTTCC | 0.544 | 0.830 | 0.737 | 0.561 | GGTAATGCCT | -0.301 | 0.807 | 0.739 | 0.658 |
| GGGATTCCTA | 0.544 | 0.748 | 0.723 | 0.598 | CGGGTTTCAC | -0.301 | 0.839 | 0.724 | 0.657 |
| GGTAGTCCCG | 0.544 | 0.792 | 0.737 | 0.599 | CTGACTTCCC | -0.301 | 0.774 | 0.707 | 0.580 |
| GTGAATTTCC | 0.544 | 0.868 | 0.835 | 0.712 | GGTGGATTGC | -0.301 | 0.757 | 0.561 | 0.500 |
| GGGGGCTCTC | 0.544 | 0.803 | 0.707 | 0.710 | GGAGTTTTCA | -0.301 | 0.807 | 0.723 | 0.607 |
| GGAATTCCTC | 0.544 | 0.769 | 0.704 | 0.571 | GGAAGTTTCG | -0.301 | 0.808 | 0.718 | 0.530 |
| GGGGCGACCC | 0.544 | 0.780 | 0.936 | 0.835 | GAGGTTTCCT | -0.301 | 0.772 | 0.742 | 0.684 |
| GGTGATGCAC | 0.544 | 0.854 | 0.740 | 0.709 | GGAACTTCAT | -0.301 | 0.764 | 0.598 | 0.474 |
| GGGGGGTCCA | 0.544 | 0.804 | 0.792 | 0.752 | GGTATATCCT | -0.301 | 0.792 | 0.646 | 0.532 |
| AGGGGGTCCC | 0.544 | 0.793 | 0.774 | 0.725 | GTTAGGTCCC | -0.301 | 0.779 | 0.628 | 0.515 |
| GGGATTGCAC | 0.544 | 0.889 | 0.825 | 0.687 | GGTGCTCCCG | -0.301 | 0.773 | 0.825 | 0.682 |
| GTGGTTTCTC | 0.539 | 0.814 | 0.724 | 0.657 | GGGAGTATGC | -0.301 | 0.767 | 0.712 | 0.602 |
| GGTCTTTTCC | 0.538 | 0.851 | 0.739 | 0.624 | TGGGCTTACC | -0.301 | 0.764 | 0.760 | 0.702 |
| CGTGTTTTCC | 0.535 | 0.839 | 0.705 | 0.580 | GGGGCTGTCA | -0.301 | 0.793 | 0.845 | 0.714 |
| GGGGTTTCAT | 0.535 | 0.840 | 0.742 | 0.684 | GGGATATCTC | -0.301 | 0.843 | 0.755 | 0.638 |
| GGGCTTTCAT | 0.531 | 0.748 | 0.650 | 0.596 | CGGTATGCCC | -0.301 | 0.751 | 0.774 | 0.753 |
| GGTGTTTTCA | 0.531 | 0.854 | 0.723 | 0.607 | GGTAGCTTAC | -0.301 | 0.793 | 0.542 | 0.423 |
| GGGAATTTGG | 0.531 | 0.787 | 0.808 | 0.612 | CGGAGTTGCC | -0.301 | 0.753 | 0.672 | 0.619 |
| GTGATTTTTC | 0.526 | 0.798 | 0.686 | 0.502 | CGTATTTCTC | -0.301 | 0.801 | 0.578 | 0.447 |
| GTGTTTGCCC | 0.526 | 0.756 | 0.752 | 0.676 | GGTATTTCGG | -0.301 | 0.795 | 0.677 | 0.480 |
| CTGGATTTCC | 0.522 | 0.754 | 0.727 | 0.657 | GGGGGTTCAG | -0.301 | 0.846 | 0.757 | 0.685 |
| GGGCGTTTCC | 0.522 | 0.863 | 0.799 | 0.752 | GGCGCTTCCC | -0.301 | 0.865 | 0.853 | 0.790 |
| GGGTATTTCA | 0.522 | 0.805 | 0.780 | 0.728 | GGCGTTATCC | -0.301 | 0.754 | 0.798 | 0.685 |
| TGGGATGTCC | 0.522 | 0.801 | 0.848 | 0.764 | GGGGCTCCTC | -0.301 | 0.809 | 0.852 | 0.782 |
| GGGATTATCT | 0.522 | 0.767 | 0.797 | 0.634 | GGGGGTTCGA | -0.301 | 0.798 | 0.675 | 0.679 |
| TGGGGGTCCC | 0.522 | 0.798 | 0.774 | 0.725 | GGTAGCGCCC | -0.301 | 0.801 | 0.681 | 0.607 |
| GGGGTATTCT | 0.522 | 0.778 | 0.773 | 0.665 | CGGCTTTCAC | -0.301 | 0.747 | 0.631 | 0.569 |
| GTGATTCTCC | 0.522 | 0.782 | 0.812 | 0.627 | GATATTTTCC | -0.301 | 0.839 | 0.686 | 0.502 |
| GGGAGTTCTC | 0.522 | 0.918 | 0.765 | 0.708 | CGTGTTTTTC | -0.301 | 0.765 | 0.578 | 0.447 |
| GGGGCTTCTT | 0.521 | 0.795 | 0.744 | 0.684 | TGTAATCTCC | -0.301 | 0.766 | 0.708 | 0.571 |
| GGTGGTTTAT | 0.519 | 0.785 | 0.529 | 0.469 | TGTATTGTAC | -0.301 | 0.747 | 0.552 | 0.344 |
| GGGGGGTCTC | 0.517 | 0.820 | 0.774 | 0.725 | GGTTCTTTCG | -0.301 | 0.769 | 0.714 | 0.524 |
| TTGGGTTTCC | 0.515 | 0.758 | 0.638 | 0.575 | GGGGCTTCAT | -0.301 | 0.822 | 0.744 | 0.684 |
| GGGTATTTCT | 0.513 | 0.791 | 0.780 | 0.728 | GGTGGGTACC | -0.301 | 0.760 | 0.682 | 0.637 |
| TGGGGTTTCA | 0.512 | 0.772 | 0.656 | 0.602 | GGGGGTACCG | -0.301 | 0.788 | 0.850 | 0.790 |
| GGTTATTTAC | 0.512 | 0.827 | 0.635 | 0.568 | GAGACATTCC | -0.301 | 0.758 | 0.737 | 0.561 |
| CGGGCTTTTC | 0.512 | 0.768 | 0.707 | 0.580 | GGGCTTATCC | -0.301 | 0.768 | 0.832 | 0.729 |
| CGTGCTTTCC | 0.501 | 0.820 | 0.707 | 0.580 | GGTGGCACCC | -0.301 | 0.752 | 0.674 | 0.682 |
| GGTGTGTCCC | 0.501 | 0.881 | 0.841 | 0.730 | CGTGGATCCC | -0.301 | 0.773 | 0.580 | 0.577 |
| GGGTATTCTC | 0.501 | 0.846 | 0.781 | 0.779 | GATATTTTAC | -0.301 | 0.792 | 0.559 | 0.369 |
| GGATGTTTTC | 0.500 | 0.747 | 0.545 | 0.486 | GGAGATTTCG | -0.301 | 0.804 | 0.827 | 0.690 |
| GGTATTTTTG | 0.493 | 0.790 | 0.658 | 0.403 | CGTAATTCCG | -0.301 | 0.782 | 0.700 | 0.557 |
| GTTATTTTCC | 0.493 | 0.850 | 0.686 | 0.502 | GGCGGTGCCC | -0.301 | 0.810 | 0.777 | 0.760 |
| TGGGGTGTCC | 0.493 | 0.796 | 0.758 | 0.682 | GTGAATTTAC | -0.301 | 0.821 | 0.708 | 0.579 |
| TGGGGTTGCC | 0.490 | 0.753 | 0.691 | 0.697 | GCTGATTTAC | -0.301 | 0.765 | 0.601 | 0.524 |
| GGTGTCTTCC | 0.490 | 0.839 | 0.755 | 0.638 | GGGAGATTGC | -0.301 | 0.788 | 0.669 | 0.556 |
| GTGGGTTTTC | 0.486 | 0.779 | 0.638 | 0.575 | GGTGCTACCA | -0.301 | 0.758 | 0.711 | 0.656 |
| GGTAATTTTT | 0.486 | 0.773 | 0.600 | 0.473 | AGGGTTGTCC | -0.301 | 0.799 | 0.825 | 0.687 |
| GGTGAATTAC | 0.477 | 0.810 | 0.650 | 0.582 | GGATCTTCAC | -0.301 | 0.791 | 0.633 | 0.569 |
| GTTATTACCC | 0.477 | 0.772 | 0.671 | 0.552 | GGTAGCTTCC | -0.301 | 0.840 | 0.669 | 0.556 |
| GGGTGTTACC | 0.477 | 0.802 | 0.726 | 0.741 | GCGCCTTCCC | -0.301 | 0.752 | 0.760 | 0.702 |
| GGGATATTCT | 0.477 | 0.788 | 0.754 | 0.587 | GGAAGTGTTC | -0.301 | 0.759 | 0.612 | 0.472 |
| TGTAATTCCC | 0.477 | 0.880 | 0.727 | 0.657 | GGTAGTTGCG | -0.301 | 0.747 | 0.645 | 0.519 |
| GGGTATGCAC | 0.477 | 0.809 | 0.774 | 0.753 | GGTGCATCGC | -0.301 | 0.773 | 0.649 | 0.582 |
| GTGGGTTCCC | 0.477 | 0.879 | 0.784 | 0.785 | GGAATTATCC | -0.301 | 0.802 | 0.779 | 0.607 |
| GGGTCCTTCC | 0.477 | 0.775 | 0.791 | 0.683 | GTTAAGTTCC | -0.301 | 0.759 | 0.698 | 0.519 |
| GGGGAGCTCC | 0.477 | 0.784 | 0.971 | 0.854 | AGCAATTCCC | -0.301 | 0.789 | 0.727 | 0.657 |
| GGTGCCCCCC | 0.477 | 0.757 | 0.775 | 0.707 | GTGATTGCTC | -0.301 | 0.759 | 0.698 | 0.554 |
| GGGCATTTAC | 0.477 | 0.822 | 0.762 | 0.701 | AGTGAATTCC | -0.301 | 0.756 | 0.650 | 0.582 |
| GGTGTTCACC | 0.477 | 0.767 | 0.758 | 0.693 | AGGCGTTCCC | -0.301 | 0.789 | 0.691 | 0.697 |
| AGGGATTCTC | 0.477 | 0.813 | 0.746 | 0.734 | GTGACCTCCC | -0.301 | 0.774 | 0.757 | 0.638 |
| TGGATTCTCC | 0.477 | 0.790 | 0.812 | 0.627 | GGTCTTGCAC | -0.301 | 0.765 | 0.625 | 0.543 |
| GGTAGTACCT | 0.477 | 0.763 | 0.623 | 0.573 | CGGGCTTCCA | -0.301 | 0.778 | 0.744 | 0.684 |
| GTTACTTCCC | 0.477 | 0.858 | 0.707 | 0.580 | GGAATTTCGA | -0.301 | 0.748 | 0.596 | 0.474 |
| GTTAATTCCC | 0.477 | 0.873 | 0.727 | 0.657 | GGCGCTCTCC | -0.301 | 0.751 | 0.833 | 0.704 |
| GGGGGTCCCG | 0.477 | 0.803 | 0.883 | 0.810 | GGTATGTCAG | -0.301 | 0.755 | 0.668 | 0.420 |
| GGGGATCTCT | 0.477 | 0.769 | 0.872 | 0.808 | GGCAATTTCC | -0.301 | 0.864 | 0.835 | 0.712 |
| TGAAATTCCC | 0.477 | 0.834 | 0.727 | 0.657 | GGAACTTCGC | -0.301 | 0.819 | 0.707 | 0.580 |
| GGGAGTTTCA | 0.477 | 0.877 | 0.764 | 0.657 | TTGAGTTCAC | -0.301 | 0.747 | 0.511 | 0.442 |
| GGTGGAGCAC | 0.477 | 0.766 | 0.574 | 0.552 | GGGAACTCTC | -0.301 | 0.818 | 0.777 | 0.715 |
| TGGAGTTTCC | 0.477 | 0.871 | 0.746 | 0.630 | GGAACTTCCG | -0.301 | 0.825 | 0.806 | 0.613 |
| GGGAGCTCCC | 0.477 | 0.887 | 0.815 | 0.766 | GCGATATCCC | -0.301 | 0.789 | 0.755 | 0.638 |
| GGGTATGTAC | 0.477 | 0.783 | 0.755 | 0.676 | TGAACTTTCC | -0.301 | 0.793 | 0.687 | 0.502 |
| GGTATGTACC | 0.477 | 0.778 | 0.729 | 0.565 | GGTAGGTTCA | -0.301 | 0.767 | 0.627 | 0.464 |
| GGTAGCCCCC | 0.477 | 0.777 | 0.687 | 0.624 | GGCGCTTTAC | -0.301 | 0.793 | 0.707 | 0.580 |
| GGTAGTGCCC | 0.477 | 0.906 | 0.758 | 0.682 | GGTGATATCA | -0.301 | 0.747 | 0.712 | 0.655 |
| GGGAGTTTAT | 0.477 | 0.816 | 0.637 | 0.524 | GGGATATTCG | -0.301 | 0.802 | 0.835 | 0.594 |
| GTATATTCCC | 0.477 | 0.749 | 0.654 | 0.646 | TGGAATTCCT | -0.301 | 0.798 | 0.746 | 0.683 |
| GGGGGTGGCC | 0.477 | 0.783 | 0.812 | 0.804 | GGTATCTCGC | -0.301 | 0.779 | 0.628 | 0.505 |
| GATATTTCCC | 0.477 | 0.865 | 0.705 | 0.580 | GGTACTTGAC | -0.301 | 0.781 | 0.614 | 0.491 |
| GAGGGGTTCC | 0.477 | 0.753 | 0.755 | 0.648 | GGTAGTCCCA | -0.301 | 0.792 | 0.656 | 0.593 |
| GGGTATCTAC | 0.477 | 0.759 | 0.761 | 0.693 | GGATATTCTC | -0.301 | 0.778 | 0.654 | 0.646 |
| GGTGTTACCC | 0.477 | 0.865 | 0.817 | 0.762 | GGGAGTCCGC | -0.301 | 0.808 | 0.764 | 0.699 |
| GGGGTTTAAC | 0.477 | 0.831 | 0.758 | 0.702 | GAGGTTTCTC | -0.301 | 0.802 | 0.724 | 0.657 |
| TGGGGTCTCC | 0.477 | 0.772 | 0.764 | 0.699 | GTTATTTTAC | -0.301 | 0.804 | 0.559 | 0.369 |
| GGGGGGGCCC | 0.477 | 0.829 | 0.894 | 0.833 | GGAATTGTCG | -0.301 | 0.752 | 0.778 | 0.510 |
| AGTGGTGTCC | 0.477 | 0.770 | 0.631 | 0.549 | GGGGATGCCT | -0.301 | 0.819 | 0.885 | 0.868 |
| GGAGTTTTGC | 0.477 | 0.802 | 0.705 | 0.580 | TGTAGTACCC | -0.301 | 0.771 | 0.605 | 0.546 |
| AGTGGTTCCC | 0.477 | 0.860 | 0.657 | 0.652 | CGTGCTGTCC | -0.301 | 0.756 | 0.700 | 0.554 |
| CGGGATTTAC | 0.477 | 0.810 | 0.727 | 0.657 | GCGGATTCCC | -0.301 | 0.859 | 0.873 | 0.867 |
| GGTACTCTTC | 0.477 | 0.772 | 0.687 | 0.494 | GGCGATATCC | -0.301 | 0.750 | 0.821 | 0.761 |
| GGAAGTTCCA | 0.477 | 0.834 | 0.656 | 0.602 | GTGCCTTTCC | -0.301 | 0.751 | 0.741 | 0.624 |
| GGGGAGTGCC | 0.477 | 0.765 | 0.898 | 0.852 | GGCGCTTTCA | -0.301 | 0.750 | 0.725 | 0.607 |
| GCGGTATTCC | 0.477 | 0.753 | 0.755 | 0.638 | GTTGATTTAC | -0.301 | 0.790 | 0.601 | 0.524 |
| GGAGTACCCC | 0.477 | 0.750 | 0.773 | 0.707 | TAGATTTTCC | -0.301 | 0.765 | 0.686 | 0.502 |
| GGTGATGTAC | 0.477 | 0.829 | 0.721 | 0.631 | AGAGATTTCC | -0.301 | 0.793 | 0.727 | 0.657 |
| GGTATTTTAT | 0.477 | 0.803 | 0.577 | 0.396 | GGAATTGTCC | -0.301 | 0.842 | 0.806 | 0.610 |
| GGTATCTACC | 0.477 | 0.762 | 0.662 | 0.550 | GGCATATCCC | -0.301 | 0.810 | 0.755 | 0.638 |
| GGTACTTCCT | 0.477 | 0.857 | 0.725 | 0.607 | ATTGCTTCCC | -0.301 | 0.747 | 0.599 | 0.524 |
| GGGAGTGCAC | 0.477 | 0.880 | 0.758 | 0.682 | GGAAATTTAG | -0.301 | 0.767 | 0.681 | 0.479 |
| GGGGTGGCCC | 0.477 | 0.837 | 0.961 | 0.838 | GGGGCTGCTC | -0.301 | 0.834 | 0.846 | 0.765 |
| GGTGCCGCCC | 0.477 | 0.781 | 0.769 | 0.690 | GGTGCGTCCT | -0.301 | 0.759 | 0.734 | 0.624 |
| GGTTCTACCC | 0.477 | 0.780 | 0.727 | 0.673 | GGGCATTCCA | -0.301 | 0.805 | 0.799 | 0.805 |
| AGTGCTTCCC | 0.477 | 0.850 | 0.726 | 0.657 | GGAAATTTGC | -0.301 | 0.808 | 0.708 | 0.579 |
| GGTTAATTCC | 0.477 | 0.790 | 0.685 | 0.626 | GGGGTGGCAC | -0.301 | 0.791 | 0.834 | 0.705 |
| GGGGTGATCC | 0.477 | 0.772 | 0.915 | 0.758 | GGGCAGTTCC | -0.301 | 0.780 | 0.879 | 0.774 |
| GGGAATGCCG | 0.477 | 0.843 | 0.947 | 0.797 | GGTAAGTTAC | -0.301 | 0.815 | 0.698 | 0.519 |
| GGTATTACCT | 0.477 | 0.771 | 0.690 | 0.578 | GGGGTCTCAC | -0.301 | 0.839 | 0.774 | 0.715 |
| GGTAGTGTCC | 0.477 | 0.880 | 0.739 | 0.605 | GGGAGTTCCA | -0.301 | 0.902 | 0.783 | 0.735 |
| GTGAATTACC | 0.477 | 0.781 | 0.762 | 0.701 | AGGATTGCAC | -0.301 | 0.788 | 0.698 | 0.554 |
| TGTGGTTTCC | 0.468 | 0.839 | 0.638 | 0.575 | GAGTTTTCAC | -0.301 | 0.763 | 0.631 | 0.569 |
| GGTGTTCCCG | 0.456 | 0.791 | 0.823 | 0.682 | GGACCTGCCC | -0.301 | 0.747 | 0.754 | 0.676 |
| GGGTCTGTCC | 0.456 | 0.815 | 0.861 | 0.732 | TGGGGGTCAC | -0.301 | 0.752 | 0.647 | 0.592 |
| GTTTATTCCC | 0.456 | 0.796 | 0.654 | 0.646 | GGTCTTTCTC | -0.301 | 0.803 | 0.631 | 0.569 |
| TGGGGTTTCT | 0.453 | 0.757 | 0.656 | 0.602 | GGAAGTTCGC | -0.301 | 0.829 | 0.638 | 0.575 |
| GGGGGTTTAG | 0.453 | 0.820 | 0.737 | 0.608 | GGTATTCCAC | -0.301 | 0.843 | 0.704 | 0.571 |
| GGGATCTTCT | 0.452 | 0.766 | 0.754 | 0.587 | GTAAATTCTC | -0.301 | 0.752 | 0.601 | 0.524 |
| GGTGGTGTAC | 0.452 | 0.823 | 0.631 | 0.549 | GGTATCGTCC | -0.301 | 0.784 | 0.729 | 0.535 |
| GGTGGTCCCT | 0.452 | 0.768 | 0.675 | 0.670 | TGTGATGCCC | -0.301 | 0.806 | 0.740 | 0.709 |
| GTGATTTTCG | 0.452 | 0.782 | 0.785 | 0.536 | CGGAACTTCC | -0.301 | 0.762 | 0.758 | 0.637 |
| GGGATGTTCT | 0.450 | 0.783 | 0.821 | 0.602 | GGGTCGTCAC | -0.301 | 0.770 | 0.750 | 0.642 |
| GGGGCCTTTC | 0.447 | 0.768 | 0.757 | 0.638 | GGGGCTGCCG | -0.301 | 0.818 | 0.945 | 0.798 |
| GGTGATTCTC | 0.447 | 0.892 | 0.746 | 0.734 | AGGGCTGCCC | -0.301 | 0.807 | 0.846 | 0.765 |
| CGGGTTTTCA | 0.447 | 0.771 | 0.723 | 0.607 | GGGGATCCAT | -0.301 | 0.748 | 0.764 | 0.752 |
| GGGATGTTGC | 0.447 | 0.791 | 0.803 | 0.576 | TCGGATTCCC | -0.301 | 0.764 | 0.746 | 0.734 |
| GGTGTTCTAC | 0.447 | 0.808 | 0.704 | 0.571 | GGTGCCTCGC | -0.301 | 0.751 | 0.649 | 0.582 |
| GGGTATTGCC | 0.439 | 0.787 | 0.815 | 0.823 | TGGGATACCC | -0.301 | 0.788 | 0.840 | 0.839 |
| GGTGATTTCT | 0.439 | 0.836 | 0.746 | 0.683 | TGAAATTTCC | -0.301 | 0.808 | 0.708 | 0.579 |
| GTGGTTCTCC | 0.436 | 0.773 | 0.831 | 0.704 | TGAATTGTCC | -0.301 | 0.747 | 0.679 | 0.477 |
| AGTTTTTTCC | 0.435 | 0.776 | 0.612 | 0.491 | GGGCGTGCCC | -0.301 | 0.824 | 0.812 | 0.804 |
| GGGGTTCCTC | 0.433 | 0.827 | 0.850 | 0.782 | GGGGCTTATC | -0.301 | 0.786 | 0.760 | 0.702 |
| GTGGCTTTCC | 0.431 | 0.843 | 0.833 | 0.713 | GGGGTCTCCG | -0.301 | 0.796 | 0.873 | 0.749 |
| TGTGCTTTCC | 0.431 | 0.830 | 0.707 | 0.580 | GGGGGCTCGC | -0.301 | 0.782 | 0.707 | 0.710 |
| TGTTTTTTCC | 0.430 | 0.781 | 0.612 | 0.491 | CGAGTTTCCC | -0.301 | 0.818 | 0.724 | 0.657 |
| TGGCTTTCCC | 0.427 | 0.803 | 0.758 | 0.702 | TCGAATTTCC | -0.301 | 0.748 | 0.708 | 0.579 |
| GGTGCTGTAC | 0.427 | 0.814 | 0.700 | 0.554 | GGTTATTCAA | -0.301 | 0.763 | 0.546 | 0.539 |
| GTGGATGTCC | 0.427 | 0.794 | 0.848 | 0.764 | CGGAATTCCT | -0.301 | 0.789 | 0.746 | 0.683 |
| GGGGTGCTCC | 0.427 | 0.787 | 0.948 | 0.777 | GGAATGTCAC | -0.301 | 0.797 | 0.695 | 0.520 |
| CCGGTTTCCC | 0.427 | 0.758 | 0.724 | 0.657 | GGTGAGTACC | -0.301 | 0.765 | 0.771 | 0.719 |
| GGGACTGTTC | 0.427 | 0.818 | 0.808 | 0.610 | GGGATTGTCA | -0.301 | 0.821 | 0.824 | 0.637 |
| GGTTTATTCC | 0.427 | 0.793 | 0.662 | 0.550 | GGTACTGCCT | -0.301 | 0.793 | 0.718 | 0.581 |
| GCGGGTTTCC | 0.427 | 0.828 | 0.765 | 0.708 | GGGACTATCT | -0.301 | 0.749 | 0.799 | 0.634 |
| GGTGAGTTCC | 0.427 | 0.852 | 0.844 | 0.730 | GTGTGTGCCC | -0.301 | 0.747 | 0.685 | 0.671 |
| TGTAATTTCC | 0.427 | 0.855 | 0.708 | 0.579 | GGTGGTGACC | -0.301 | 0.783 | 0.685 | 0.671 |
| TGGGATTCTC | 0.427 | 0.818 | 0.746 | 0.734 | GCTTATTCCC | -0.301 | 0.771 | 0.654 | 0.646 |
| TGGTTTTCTC | 0.420 | 0.755 | 0.631 | 0.569 | GGGAGTCTCA | -0.301 | 0.788 | 0.763 | 0.649 |
| GGGGCTTCCT | 0.420 | 0.869 | 0.871 | 0.817 | GGGTAGTCAC | -0.301 | 0.785 | 0.771 | 0.719 |
| GGTATTTTCT | 0.417 | 0.850 | 0.704 | 0.529 | GATATTTCCT | -0.301 | 0.761 | 0.596 | 0.474 |
| TTTAGTTCCC | 0.415 | 0.772 | 0.511 | 0.442 | GGAGCTTTAC | -0.301 | 0.832 | 0.707 | 0.580 |
| GGGCATTTTC | 0.415 | 0.795 | 0.762 | 0.701 | TGGATATTCC | -0.301 | 0.796 | 0.735 | 0.561 |
| CGGGTTTTAC | 0.415 | 0.813 | 0.705 | 0.580 | GATGTTTTAC | -0.301 | 0.782 | 0.578 | 0.447 |
| GGGGAGTTCT | 0.410 | 0.769 | 0.863 | 0.757 | GGGCGTTCCA | -0.301 | 0.800 | 0.710 | 0.723 |
| GGGTGTTCTC | 0.410 | 0.841 | 0.691 | 0.697 | CGTACTTCGC | -0.301 | 0.761 | 0.580 | 0.447 |
| ATGGTTTTCC | 0.408 | 0.761 | 0.705 | 0.580 | GGGAATTCAT | -0.301 | 0.847 | 0.746 | 0.683 |
| GGTGGTTTCG | 0.400 | 0.845 | 0.737 | 0.608 | TGTGATTCAC | -0.301 | 0.824 | 0.620 | 0.601 |
| GGAGCTTCCC | 0.398 | 0.904 | 0.853 | 0.790 | TGTGTTTTTC | -0.310 | 0.774 | 0.578 | 0.447 |
| GGGAATCCTC | 0.398 | 0.834 | 0.854 | 0.781 | TGTGTTTTGC | -0.310 | 0.753 | 0.578 | 0.447 |
| GGGTCTACCC | 0.398 | 0.801 | 0.854 | 0.806 | TGGGTTTTGC | -0.310 | 0.774 | 0.705 | 0.580 |
| GGGACTTTCA | 0.398 | 0.867 | 0.833 | 0.662 | GGTGTTCTCG | -0.319 | 0.765 | 0.804 | 0.604 |
| GGTGTTCCCA | 0.398 | 0.791 | 0.742 | 0.676 | GTGGGTTTCT | -0.319 | 0.750 | 0.656 | 0.602 |
| AGGATTTTCC | 0.398 | 0.874 | 0.812 | 0.635 | TGGGGTTCCT | -0.319 | 0.783 | 0.675 | 0.679 |
| GGGGTAGCAC | 0.398 | 0.796 | 0.767 | 0.690 | GGGGTTGTGC | -0.319 | 0.805 | 0.825 | 0.687 |
| GGTAGTTTTA | 0.398 | 0.782 | 0.510 | 0.391 | TGTAGTTTCG | -0.319 | 0.760 | 0.591 | 0.397 |
| GGTATTACCG | 0.398 | 0.785 | 0.771 | 0.585 | TGTGTTTCCT | -0.328 | 0.770 | 0.615 | 0.551 |
| TGTAGTCCCC | 0.398 | 0.786 | 0.638 | 0.566 | GGGGTTGCTT | -0.328 | 0.748 | 0.736 | 0.659 |
| GGTAATTCCT | 0.398 | 0.872 | 0.746 | 0.683 | GGGGTTGCTG | -0.328 | 0.762 | 0.817 | 0.665 |
| GGGACCCTCC | 0.398 | 0.762 | 0.864 | 0.685 | TGGATTTTCG | -0.337 | 0.790 | 0.785 | 0.536 |
| GGGGTATTCA | 0.398 | 0.792 | 0.773 | 0.665 | TGTGCTTCCG | -0.337 | 0.766 | 0.698 | 0.557 |
| GGAATTTTCA | 0.398 | 0.817 | 0.704 | 0.529 | GGGCGTTTCG | -0.337 | 0.774 | 0.772 | 0.652 |
| ATGGATTCCC | 0.398 | 0.783 | 0.746 | 0.734 | TGGGGTTCGC | -0.337 | 0.792 | 0.657 | 0.652 |
| GAAGTTTCCC | 0.398 | 0.808 | 0.724 | 0.657 | CGTTGTTTCC | -0.337 | 0.763 | 0.545 | 0.486 |
| GGTACCGTCC | 0.398 | 0.766 | 0.731 | 0.535 | TGTGTTTCGC | -0.337 | 0.779 | 0.597 | 0.524 |
| GGGAAGTTTC | 0.398 | 0.809 | 0.825 | 0.652 | GGGTTTGCTC | -0.337 | 0.785 | 0.752 | 0.676 |
| GGTATATTCC | 0.398 | 0.870 | 0.735 | 0.561 | GGGTGTTCCG | -0.337 | 0.826 | 0.791 | 0.730 |
| GTGGACTTCC | 0.398 | 0.753 | 0.777 | 0.715 | GTTGGTTTCC | -0.337 | 0.832 | 0.638 | 0.575 |
| GGAATTTTAC | 0.398 | 0.860 | 0.686 | 0.502 | GGTGGTGCCT | -0.347 | 0.792 | 0.669 | 0.653 |
| GGGGAGTCTC | 0.398 | 0.825 | 0.864 | 0.807 | GGTTCTTTGC | -0.347 | 0.764 | 0.614 | 0.491 |
| AGGGGTTTCA | 0.398 | 0.766 | 0.656 | 0.602 | GAGGTTTTCG | -0.347 | 0.761 | 0.804 | 0.613 |
| GGAATTTCCA | 0.398 | 0.843 | 0.723 | 0.607 | CGGGTTTCCT | -0.347 | 0.782 | 0.742 | 0.684 |
| GGGGATCGCC | 0.398 | 0.764 | 0.907 | 0.903 | CGTGTGTTCC | -0.347 | 0.750 | 0.695 | 0.520 |
| GGGGTATACC | 0.398 | 0.794 | 0.808 | 0.760 | CGTATTTTCG | -0.347 | 0.759 | 0.658 | 0.403 |
| GGTACTGCAC | 0.398 | 0.850 | 0.700 | 0.554 | TGTGTTTACC | -0.347 | 0.761 | 0.631 | 0.569 |
| GGTGCTATCC | 0.398 | 0.821 | 0.800 | 0.685 | CTGGTTTCCC | -0.357 | 0.783 | 0.724 | 0.657 |
| GGGGACTCCT | 0.398 | 0.779 | 0.815 | 0.819 | GGTGTATTCT | -0.357 | 0.757 | 0.646 | 0.532 |
| GGGATCGCCC | 0.398 | 0.831 | 0.875 | 0.746 | GGTTCTTTCA | -0.357 | 0.769 | 0.633 | 0.518 |
| GGTTGTACCC | 0.398 | 0.790 | 0.658 | 0.668 | GTTGTTTCCA | -0.357 | 0.777 | 0.615 | 0.551 |
| GGGAACTCCT | 0.398 | 0.789 | 0.796 | 0.742 | GGTTTGTTCC | -0.357 | 0.788 | 0.729 | 0.565 |
| TGGCCTTCCC | 0.398 | 0.784 | 0.760 | 0.702 | CGTTTTTTCC | -0.357 | 0.772 | 0.612 | 0.491 |
| GGTGCCTCCC | 0.398 | 0.846 | 0.776 | 0.715 | CTTGTTTCCC | -0.357 | 0.762 | 0.597 | 0.524 |
| GGGCATTCCT | 0.398 | 0.791 | 0.799 | 0.805 | TGGGATTTCG | -0.357 | 0.776 | 0.827 | 0.690 |
| GGGTGATTAC | 0.398 | 0.759 | 0.595 | 0.544 | GGTGGTTTGA | -0.357 | 0.751 | 0.529 | 0.469 |
| GGTACGTTCC | 0.398 | 0.847 | 0.805 | 0.576 | GGTGTCTCCT | -0.357 | 0.761 | 0.665 | 0.609 |
| GGGGAATTCG | 0.398 | 0.788 | 0.877 | 0.748 | TGTATTTCGC | -0.357 | 0.789 | 0.578 | 0.447 |
| GGGATTTAGC | 0.398 | 0.793 | 0.739 | 0.624 | TGTGCTTTAC | -0.357 | 0.783 | 0.580 | 0.447 |
| GGAACTTTTC | 0.398 | 0.814 | 0.687 | 0.502 | GGTTATTCTC | -0.357 | 0.825 | 0.654 | 0.646 |
| GTGATTTCCA | 0.398 | 0.808 | 0.723 | 0.607 | GATGTTTCCT | -0.367 | 0.751 | 0.615 | 0.551 |
| GGGGAATTGC | 0.398 | 0.783 | 0.777 | 0.715 | GTTAATTTTC | -0.367 | 0.773 | 0.581 | 0.446 |
| GGGCTTTCAC | 0.398 | 0.851 | 0.758 | 0.702 | GGGGTTTGAC | -0.367 | 0.810 | 0.758 | 0.702 |
| GGTTGATCCC | 0.398 | 0.810 | 0.615 | 0.622 | GGTTGTTTAC | -0.367 | 0.821 | 0.545 | 0.486 |
| TGGTTTTCAC | 0.398 | 0.782 | 0.631 | 0.569 | GGTATTGTCT | -0.367 | 0.785 | 0.697 | 0.504 |
| GGGATCTTAC | 0.398 | 0.823 | 0.735 | 0.561 | GGGTCTTCGC | -0.367 | 0.810 | 0.760 | 0.702 |
| GTTGATCCCC | 0.398 | 0.774 | 0.746 | 0.726 | TGTGTTTTCG | -0.367 | 0.759 | 0.677 | 0.480 |
| GGGGATTGCA | 0.398 | 0.764 | 0.799 | 0.805 | GGTGTTTACT | -0.367 | 0.753 | 0.650 | 0.596 |
| GGGGGTTGCA | 0.398 | 0.759 | 0.710 | 0.723 | CGTGGTTCCG | -0.367 | 0.767 | 0.630 | 0.552 |
| GGGGTTACAC | 0.398 | 0.839 | 0.817 | 0.762 | GGTTATTCCG | -0.367 | 0.810 | 0.754 | 0.679 |
| GGTGTAGTAC | 0.398 | 0.749 | 0.621 | 0.480 | GGTGGTTCGC | -0.367 | 0.866 | 0.657 | 0.652 |
| GTGATTTTAC | 0.398 | 0.825 | 0.686 | 0.502 | TGGATTTTTC | -0.367 | 0.805 | 0.686 | 0.502 |
| GGTACACTCC | 0.398 | 0.763 | 0.737 | 0.552 | TGTGGTTCGC | -0.367 | 0.771 | 0.530 | 0.519 |
| GGTGTTATCC | 0.398 | 0.839 | 0.798 | 0.685 | GTGGATTTCG | -0.377 | 0.769 | 0.827 | 0.690 |
| GATATTCCCC | 0.398 | 0.776 | 0.704 | 0.571 | CGGGGTTTCG | -0.377 | 0.762 | 0.737 | 0.608 |
| GGGGCTCTAC | 0.398 | 0.811 | 0.833 | 0.704 | TGGATTTTCT | -0.377 | 0.776 | 0.704 | 0.529 |
| GGGAGTTGCC | 0.398 | 0.858 | 0.799 | 0.752 | GGTGTTTCGT | -0.377 | 0.771 | 0.615 | 0.551 |
| GGAATTCTCC | 0.398 | 0.817 | 0.812 | 0.627 | TGTGTTTCTC | -0.377 | 0.800 | 0.597 | 0.524 |
| CGGATTCCCC | 0.398 | 0.807 | 0.831 | 0.704 | CGTGTTTCCT | -0.377 | 0.761 | 0.615 | 0.551 |
| GGGGGGGTAC | 0.398 | 0.756 | 0.748 | 0.622 | GGTGATTCGT | -0.377 | 0.767 | 0.638 | 0.628 |
| GGTGTTTTAT | 0.396 | 0.793 | 0.596 | 0.474 | GGTGTTGCGC | -0.377 | 0.810 | 0.717 | 0.632 |
| GGTGTTTTCG | 0.396 | 0.854 | 0.804 | 0.613 | TGTGGTTTCA | -0.377 | 0.750 | 0.529 | 0.469 |
| GCGGTTTTCC | 0.389 | 0.836 | 0.831 | 0.713 | GGTGTTGTTC | -0.377 | 0.805 | 0.698 | 0.554 |
| GTTTCTTCCC | 0.384 | 0.781 | 0.633 | 0.569 | GGGGGTTCGT | -0.377 | 0.783 | 0.675 | 0.679 |
| TTGGATTTCC | 0.384 | 0.763 | 0.727 | 0.657 | GGTGATTCTG | -0.377 | 0.803 | 0.719 | 0.634 |
| GGTGGTTGCC | 0.384 | 0.827 | 0.691 | 0.697 | TGTGGTTTCG | -0.387 | 0.750 | 0.611 | 0.475 |
| GGGTCTTTAC | 0.380 | 0.833 | 0.741 | 0.624 | GTGAGTTTTC | -0.387 | 0.789 | 0.619 | 0.497 |
| GGGGGTTCCG | 0.380 | 0.892 | 0.883 | 0.818 | GGTATGTTCT | -0.387 | 0.761 | 0.694 | 0.469 |
| GTGTATTTCC | 0.380 | 0.791 | 0.762 | 0.701 | GGTGTTGCCT | -0.387 | 0.801 | 0.736 | 0.659 |
| CGGGTTGTCC | 0.380 | 0.795 | 0.825 | 0.687 | GTTGTTTCCG | -0.387 | 0.777 | 0.697 | 0.557 |
| GGTATTTTCG | 0.375 | 0.864 | 0.785 | 0.536 | GTTGTTTTTC | -0.387 | 0.767 | 0.578 | 0.447 |
| GTGGAGTTCC | 0.367 | 0.770 | 0.844 | 0.730 | GGTCTTTTCG | -0.387 | 0.761 | 0.712 | 0.524 |
| TTTACTTCCC | 0.367 | 0.763 | 0.580 | 0.447 | GGGTGTGCCG | -0.398 | 0.761 | 0.784 | 0.704 |
| GGGACGTTTC | 0.367 | 0.794 | 0.805 | 0.576 | GTTGGTGCCC | -0.398 | 0.793 | 0.650 | 0.627 |
| GTAGCTTTCC | 0.367 | 0.775 | 0.707 | 0.580 | GGTAGTCTTC | -0.398 | 0.782 | 0.618 | 0.489 |
| GGAGTTGTCC | 0.367 | 0.832 | 0.825 | 0.687 | GGAGGTGTTC | -0.398 | 0.750 | 0.631 | 0.549 |
| GATGTTTTCC | 0.367 | 0.829 | 0.705 | 0.580 | GAGGCTTTTC | -0.398 | 0.758 | 0.707 | 0.580 |
| TGGGGTTCCA | 0.367 | 0.797 | 0.675 | 0.679 | GGTTGTTTCT | -0.398 | 0.765 | 0.564 | 0.513 |
| GGGGTTCCAT | 0.367 | 0.751 | 0.742 | 0.676 | GGTCCTTTTC | -0.398 | 0.759 | 0.614 | 0.491 |
| GTGACTTTCC | 0.367 | 0.853 | 0.814 | 0.635 | GGGGTGTCGC | -0.398 | 0.807 | 0.841 | 0.730 |
| GGGATTGTAG | 0.367 | 0.774 | 0.778 | 0.510 | GGTAGGTTGC | -0.398 | 0.762 | 0.609 | 0.438 |
| TGTAGTTCCC | 0.367 | 0.875 | 0.638 | 0.575 | GGCTCTTTCC | -0.398 | 0.773 | 0.741 | 0.624 |
| AGGGGTTTGC | 0.360 | 0.761 | 0.638 | 0.575 | CGGAGTTTCT | -0.398 | 0.758 | 0.637 | 0.524 |
| GGGGTTTCGT | 0.358 | 0.792 | 0.742 | 0.684 | GGTGATTGTC | -0.398 | 0.758 | 0.654 | 0.646 |
| GGTGGTTCTC | 0.350 | 0.887 | 0.657 | 0.652 | GCGATTTTGC | -0.398 | 0.752 | 0.686 | 0.502 |
| GGTGGTTCCT | 0.348 | 0.857 | 0.675 | 0.679 | GTTAGTGTCC | -0.398 | 0.777 | 0.612 | 0.472 |
| GTGTTTTCTC | 0.346 | 0.747 | 0.631 | 0.569 | GGGAGGTTCT | -0.398 | 0.774 | 0.754 | 0.597 |
| GGAGTTTCTC | 0.342 | 0.848 | 0.724 | 0.657 | TGGGATTCCG | -0.398 | 0.802 | 0.846 | 0.767 |
| TGGGTTCCCC | 0.342 | 0.806 | 0.850 | 0.782 | CGTGTTCTCC | -0.398 | 0.750 | 0.704 | 0.571 |
| GGGTGTTTCA | 0.342 | 0.800 | 0.691 | 0.646 | GGTAATTTCT | -0.398 | 0.846 | 0.726 | 0.606 |
| GGGACTTTGT | 0.340 | 0.758 | 0.706 | 0.529 | TGGAGTTCTC | -0.398 | 0.823 | 0.638 | 0.575 |
| GGTATTTTGC | 0.338 | 0.858 | 0.686 | 0.502 | AGTCTTTTCC | -0.398 | 0.750 | 0.612 | 0.491 |
| GGAATTTCTT | 0.336 | 0.755 | 0.596 | 0.474 | GGCGGTTCTC | -0.398 | 0.801 | 0.657 | 0.652 |
| GGGTATTCCT | 0.336 | 0.817 | 0.799 | 0.805 | GTGATGTTCC | -0.398 | 0.783 | 0.803 | 0.576 |
| GGTGTTTCTC | 0.334 | 0.895 | 0.724 | 0.657 | GGGTGTCCTC | -0.398 | 0.752 | 0.691 | 0.688 |
| GGGATTGTTG | 0.332 | 0.747 | 0.778 | 0.510 | GGTTGTATCC | -0.398 | 0.764 | 0.639 | 0.591 |
| GGTTATTTTC | 0.330 | 0.799 | 0.635 | 0.568 | GGTGCTTCTA | -0.398 | 0.788 | 0.617 | 0.551 |
| GTGATTTCTC | 0.328 | 0.823 | 0.705 | 0.580 | GGTGGTTTAA | -0.398 | 0.799 | 0.529 | 0.469 |
| GGGGTTTCGG | 0.324 | 0.806 | 0.823 | 0.690 | GGTGGGTTCA | -0.398 | 0.757 | 0.647 | 0.542 |
| GGGATTTGTC | 0.316 | 0.793 | 0.739 | 0.624 | TGGAATTTCT | -0.398 | 0.772 | 0.726 | 0.606 |
| GGGGGTTCTG | 0.316 | 0.818 | 0.757 | 0.685 | GAGTGTTTCC | -0.398 | 0.775 | 0.672 | 0.619 |
| AGGGTTTTCT | 0.312 | 0.760 | 0.723 | 0.607 | GGTATGTTCC | -0.398 | 0.865 | 0.803 | 0.576 |
| AGGGATGTCC | 0.301 | 0.796 | 0.848 | 0.764 | GGGTCTTTCA | -0.398 | 0.790 | 0.759 | 0.651 |
| GGGGTATGCC | 0.301 | 0.773 | 0.808 | 0.760 | CGTGATTTTC | -0.398 | 0.762 | 0.601 | 0.524 |
| CGGGGTTACC | 0.301 | 0.764 | 0.691 | 0.697 | CGGCTTTTCC | -0.398 | 0.767 | 0.739 | 0.624 |
| GGGAATGTCT | 0.301 | 0.803 | 0.847 | 0.714 | GGTGGGTCGC | -0.398 | 0.777 | 0.647 | 0.592 |
| GGGACCTTTC | 0.301 | 0.778 | 0.737 | 0.561 | GCGAGTTTTC | -0.398 | 0.764 | 0.619 | 0.497 |
| GGGACTTTAG | 0.301 | 0.820 | 0.787 | 0.536 | GCGGTTGTCC | -0.398 | 0.772 | 0.825 | 0.687 |
| GGGTAGTTAC | 0.301 | 0.759 | 0.752 | 0.641 | GAGGTTTTGC | -0.398 | 0.755 | 0.705 | 0.580 |
| GGGGTTTCAG | 0.301 | 0.854 | 0.823 | 0.690 | GTGGGTTTAC | -0.398 | 0.806 | 0.638 | 0.575 |
| GGGTCTTCAC | 0.301 | 0.859 | 0.760 | 0.702 | GGTATTGCTG | -0.398 | 0.751 | 0.671 | 0.454 |
| TGGATTGTAC | 0.301 | 0.768 | 0.679 | 0.477 | GGGTCTTCTC | -0.398 | 0.831 | 0.760 | 0.702 |
| GGGGATTCGC | 0.301 | 0.892 | 0.873 | 0.867 | GGTCTTCTCC | -0.398 | 0.762 | 0.739 | 0.616 |
| AGGGTTTTCA | 0.301 | 0.775 | 0.723 | 0.607 | TGTGGTTCCG | -0.398 | 0.776 | 0.630 | 0.552 |
| CGTGTTTCCC | 0.301 | 0.864 | 0.724 | 0.657 | GTGGCTTTCG | -0.409 | 0.754 | 0.806 | 0.613 |
| GGGGTTCTCT | 0.301 | 0.772 | 0.849 | 0.731 | GGGGCTTTGG | -0.409 | 0.762 | 0.806 | 0.613 |
| GGTATAGTCC | 0.301 | 0.805 | 0.729 | 0.535 | TGTAGTTTTC | -0.409 | 0.776 | 0.492 | 0.364 |
| GTGGTTTCAC | 0.301 | 0.841 | 0.724 | 0.657 | GTTGTTTTAC | -0.409 | 0.794 | 0.578 | 0.447 |
| GGGATGTCCA | 0.301 | 0.823 | 0.840 | 0.680 | GGTGTGTTTC | -0.409 | 0.781 | 0.695 | 0.520 |
| CGTAATTTCC | 0.301 | 0.845 | 0.708 | 0.579 | GGTGCTTCCG | -0.420 | 0.861 | 0.825 | 0.690 |
| GAGCTTTCCC | 0.301 | 0.783 | 0.758 | 0.702 | GGTGGTGCTC | -0.420 | 0.822 | 0.650 | 0.627 |
| AGTTTTTCAC | 0.301 | 0.755 | 0.505 | 0.436 | TGGACTTTTC | -0.420 | 0.787 | 0.687 | 0.502 |
| GGGAGCTTCC | 0.301 | 0.861 | 0.795 | 0.688 | GGTAGGTTTC | -0.420 | 0.783 | 0.609 | 0.438 |
| GGAATTTTAG | 0.301 | 0.770 | 0.658 | 0.403 | GGTGGTTCCG | -0.420 | 0.871 | 0.757 | 0.685 |
| GTAAGTTCCC | 0.301 | 0.821 | 0.638 | 0.575 | GTTGCTTTAC | -0.420 | 0.776 | 0.580 | 0.447 |
| GGGCCTCTCC | 0.301 | 0.765 | 0.867 | 0.749 | GGTGTTTTAA | -0.420 | 0.808 | 0.596 | 0.474 |
| TGGAAGTTCC | 0.301 | 0.787 | 0.825 | 0.652 | GTTGTTTCGC | -0.420 | 0.771 | 0.597 | 0.524 |
| GGACCTTTCC | 0.301 | 0.786 | 0.741 | 0.624 | TGGGCTTTGC | -0.420 | 0.756 | 0.707 | 0.580 |
| CGTTATTCCC | 0.301 | 0.794 | 0.654 | 0.646 | GGGGGTTGGC | -0.420 | 0.753 | 0.691 | 0.697 |
| GGTAATTTAC | 0.301 | 0.903 | 0.708 | 0.579 | TGTATTTCCT | -0.420 | 0.780 | 0.596 | 0.474 |
| CGGAGTTTCC | 0.301 | 0.861 | 0.746 | 0.630 | GCGATTTTTC | -0.420 | 0.773 | 0.686 | 0.502 |
| GGGGGAGCTC | 0.301 | 0.760 | 0.700 | 0.685 | GTTGATTCTC | -0.420 | 0.789 | 0.620 | 0.601 |
| GGGATGGTCC | 0.301 | 0.821 | 0.923 | 0.683 | GGTGTCTTCG | -0.432 | 0.749 | 0.727 | 0.538 |
| GTGAATTCCG | 0.301 | 0.804 | 0.827 | 0.690 | TGGGTTTCGC | -0.432 | 0.800 | 0.724 | 0.657 |
| AGGGTTATCC | 0.301 | 0.760 | 0.798 | 0.685 | CGTGTTTCTC | -0.432 | 0.791 | 0.597 | 0.524 |
| GTGATTTTCT | 0.301 | 0.768 | 0.704 | 0.529 | GGTGTTCTCT | -0.432 | 0.751 | 0.723 | 0.598 |
| GGGCATTCAC | 0.301 | 0.848 | 0.781 | 0.779 | GGTGTTTCGA | -0.444 | 0.785 | 0.615 | 0.551 |
| GGGACTACCG | 0.301 | 0.788 | 0.900 | 0.718 | GGTCGTTCTC | -0.444 | 0.794 | 0.565 | 0.564 |
| GGGCGTTACC | 0.301 | 0.776 | 0.726 | 0.741 | GGTAGTTCGT | -0.444 | 0.772 | 0.529 | 0.469 |
| GGTACTTTCG | 0.301 | 0.846 | 0.787 | 0.536 | GGGGCGTTCT | -0.444 | 0.754 | 0.842 | 0.680 |
| GGGAATCTTC | 0.301 | 0.808 | 0.835 | 0.704 | GCTGTTGTCC | -0.444 | 0.751 | 0.698 | 0.554 |
| GGAAATTTCG | 0.301 | 0.814 | 0.808 | 0.612 | TGTACTTTCG | -0.444 | 0.750 | 0.660 | 0.403 |
| TGTGTTTTCC | 0.301 | 0.848 | 0.705 | 0.580 | GTTGCTTTCC | -0.444 | 0.822 | 0.707 | 0.580 |
| TGGCATTTCC | 0.301 | 0.773 | 0.762 | 0.701 | GGTGGTTTGG | -0.444 | 0.751 | 0.611 | 0.475 |
| GGTATTTTCA | 0.301 | 0.864 | 0.704 | 0.529 | TGGCGTTTCC | -0.444 | 0.768 | 0.672 | 0.619 |
| TGGATGTCCC | 0.301 | 0.817 | 0.822 | 0.653 | CGTGTTTCGC | -0.444 | 0.770 | 0.597 | 0.524 |
| GGGACTCTCT | 0.301 | 0.764 | 0.832 | 0.654 | GTTGTTTCCT | -0.456 | 0.763 | 0.615 | 0.551 |
| GGGACTTCGA | 0.301 | 0.798 | 0.725 | 0.607 | GCTGTTTCTC | -0.456 | 0.767 | 0.597 | 0.524 |
| GGGGGTTAAC | 0.301 | 0.822 | 0.691 | 0.697 | CGTGTTTTCG | -0.456 | 0.749 | 0.677 | 0.480 |
| GGGTAATTAC | 0.301 | 0.764 | 0.685 | 0.626 | GGTTGTTCCT | -0.456 | 0.790 | 0.583 | 0.591 |
| CCGGATTCCC | 0.301 | 0.754 | 0.746 | 0.734 | TGGGCTTTCG | -0.456 | 0.762 | 0.806 | 0.613 |
| GGGGACTTCG | 0.301 | 0.767 | 0.877 | 0.748 | GGTGGTGTCG | -0.456 | 0.781 | 0.731 | 0.582 |
| GGCATTTACC | 0.301 | 0.781 | 0.739 | 0.624 | CGTATTTTTC | -0.456 | 0.775 | 0.559 | 0.369 |
| GGGATAGTAC | 0.301 | 0.780 | 0.729 | 0.535 | GGTCATTTGC | -0.481 | 0.753 | 0.635 | 0.568 |
| GGCATTTTCC | 0.301 | 0.868 | 0.812 | 0.635 | GTGGGTGCCC | -0.481 | 0.814 | 0.777 | 0.760 |
| GGGAGTTCCT | 0.301 | 0.888 | 0.783 | 0.735 | GCGGGTGTCC | -0.481 | 0.763 | 0.758 | 0.682 |
| TAGGATTCCC | 0.301 | 0.777 | 0.746 | 0.734 | CGGTTTGCCC | -0.481 | 0.754 | 0.752 | 0.676 |
| GGGGATTTGG | 0.301 | 0.777 | 0.827 | 0.690 | GTTGGTTCCG | -0.481 | 0.768 | 0.630 | 0.552 |
| CGGGATTCAC | 0.301 | 0.836 | 0.746 | 0.734 | GGTATTGTAC | -0.481 | 0.842 | 0.679 | 0.477 |
| GGGACTTCTG | 0.301 | 0.819 | 0.806 | 0.613 | ATTGTTTCCC | -0.481 | 0.766 | 0.597 | 0.524 |
| TGTGAATTCC | 0.301 | 0.761 | 0.650 | 0.582 | GTGACTTCTC | -0.481 | 0.805 | 0.707 | 0.580 |
| GGGGATTGAC | 0.301 | 0.807 | 0.781 | 0.779 | GGGTGTTCAG | -0.481 | 0.779 | 0.664 | 0.597 |
| AGGGATTTCT | 0.301 | 0.757 | 0.746 | 0.683 | TGTCATTCCC | -0.481 | 0.778 | 0.654 | 0.646 |
| GGGGAGTCCG | 0.301 | 0.809 | 0.963 | 0.840 | GCTAGTTTCC | -0.481 | 0.817 | 0.619 | 0.497 |
| GGGATTTACA | 0.301 | 0.798 | 0.757 | 0.651 | TTGGCTTCCC | -0.481 | 0.774 | 0.726 | 0.657 |
| CGGGATTTCA | 0.301 | 0.767 | 0.746 | 0.683 | TGTTATTCAC | -0.481 | 0.757 | 0.527 | 0.513 |
| CGTGAATTCC | 0.301 | 0.752 | 0.650 | 0.582 | GTTACTTCCG | -0.481 | 0.769 | 0.679 | 0.480 |
| TGGGATGCTC | 0.301 | 0.753 | 0.740 | 0.709 | GGTGGCTCCG | -0.481 | 0.766 | 0.680 | 0.610 |
| CGGGCGTTCC | 0.301 | 0.753 | 0.824 | 0.653 | GGTCTTGCCC | -0.481 | 0.812 | 0.752 | 0.676 |
| GCGGATTTCC | 0.301 | 0.833 | 0.854 | 0.790 | GGTGTATTCA | -0.481 | 0.771 | 0.646 | 0.532 |
| GGGCCCTCCC | 0.301 | 0.775 | 0.810 | 0.760 | GGTGGTTCGA | -0.481 | 0.777 | 0.549 | 0.546 |
| AGTGGTTTTC | 0.301 | 0.760 | 0.511 | 0.442 | GGTCGGTTCC | -0.481 | 0.754 | 0.663 | 0.559 |
| GGGGTTATAC | 0.301 | 0.814 | 0.798 | 0.685 | GTTACTTCGC | -0.481 | 0.763 | 0.580 | 0.447 |
| GGGTAATCTC | 0.301 | 0.763 | 0.704 | 0.704 | GGGGTATCCT | -0.481 | 0.803 | 0.792 | 0.742 |
| GGGCCTGTCC | 0.301 | 0.789 | 0.861 | 0.732 | GGGCCTTCTC | -0.481 | 0.806 | 0.760 | 0.702 |
| GATGTTCCCC | 0.301 | 0.766 | 0.723 | 0.649 | GGATGTTCCG | -0.481 | 0.758 | 0.664 | 0.597 |
| CGGGGTCTCC | 0.301 | 0.762 | 0.764 | 0.699 | GGGGTGGCCG | -0.481 | 0.748 | 0.934 | 0.738 |
| GGGGGTAACC | 0.301 | 0.765 | 0.785 | 0.801 | GGTCTATTCC | -0.481 | 0.768 | 0.662 | 0.550 |
| TGTGGTTACC | 0.301 | 0.753 | 0.565 | 0.564 | GGTATGGTCC | -0.481 | 0.800 | 0.796 | 0.550 |
| TGGGATTGCC | 0.301 | 0.758 | 0.781 | 0.779 | GGTAGTTCGC | -0.481 | 0.876 | 0.638 | 0.575 |
| GAGGTGTTCC | 0.301 | 0.762 | 0.822 | 0.653 | GGTAGTCTGC | -0.481 | 0.761 | 0.618 | 0.489 |
| GGTATTTCTC | 0.301 | 0.905 | 0.705 | 0.580 | GCGGGTTCGC | -0.481 | 0.759 | 0.657 | 0.652 |
| GGGATCGTCC | 0.301 | 0.805 | 0.856 | 0.668 | GGGAGTGCTG | -0.481 | 0.764 | 0.731 | 0.582 |
| GGTAGTACCG | 0.301 | 0.777 | 0.704 | 0.579 | GCTATTTCCG | -0.481 | 0.762 | 0.677 | 0.480 |
| AGTGTTCCCC | 0.301 | 0.780 | 0.723 | 0.649 | TGGATTGCCG | -0.481 | 0.751 | 0.798 | 0.587 |
| GGGGGTACTC | 0.301 | 0.804 | 0.750 | 0.757 | GGCGTTTCCG | -0.481 | 0.794 | 0.823 | 0.690 |
| GGTACATTAC | 0.301 | 0.805 | 0.611 | 0.428 | GTTGGATTCC | -0.481 | 0.749 | 0.561 | 0.500 |
| GGGATTGACC | 0.301 | 0.823 | 0.859 | 0.732 | GGTATTGCCG | -0.481 | 0.825 | 0.798 | 0.587 |
| TGGGGTTACC | 0.301 | 0.774 | 0.691 | 0.697 | GCGTCTTTCC | -0.481 | 0.752 | 0.741 | 0.624 |
| TGGGATCCAC | 0.301 | 0.756 | 0.746 | 0.726 | GGTACTTTAG | -0.481 | 0.799 | 0.660 | 0.403 |
| AGGGGTGTCC | 0.301 | 0.791 | 0.758 | 0.682 | TGGTGTTCAC | -0.481 | 0.773 | 0.565 | 0.564 |
| GGTTCTCCAC | 0.301 | 0.749 | 0.633 | 0.560 | GGGGGTGCTC | -0.481 | 0.843 | 0.777 | 0.760 |
| ATGGTTTCCC | 0.301 | 0.787 | 0.724 | 0.657 | GGGAGGTTCG | -0.481 | 0.788 | 0.835 | 0.604 |
| GGGTGTACCC | 0.301 | 0.811 | 0.785 | 0.801 | GTGACTTCCT | -0.481 | 0.776 | 0.725 | 0.607 |
| TTTGCTTCCC | 0.301 | 0.753 | 0.599 | 0.524 | GTCGTTTTCC | -0.481 | 0.755 | 0.705 | 0.580 |
| GGACGTTTCC | 0.301 | 0.795 | 0.672 | 0.619 | TGGGTTTCAA | -0.481 | 0.759 | 0.615 | 0.551 |
| GGAATTTCAC | 0.301 | 0.886 | 0.705 | 0.580 | CGGGCTTTGC | -0.481 | 0.747 | 0.707 | 0.580 |
| GGGGATATCT | 0.301 | 0.753 | 0.839 | 0.788 | GGGAGTTGCT | -0.481 | 0.755 | 0.691 | 0.646 |
| GGGGGGCCAC | 0.301 | 0.758 | 0.774 | 0.717 | GTGGATTCGC | -0.481 | 0.789 | 0.746 | 0.734 |
| GGGGCTGTTC | 0.301 | 0.808 | 0.827 | 0.687 | GATGATTTTC | -0.481 | 0.752 | 0.601 | 0.524 |
| GGTGTTTCCT | 0.297 | 0.866 | 0.742 | 0.684 | CGTGTTTCCG | -0.481 | 0.775 | 0.697 | 0.557 |
| TTTGGTTCCC | 0.294 | 0.762 | 0.530 | 0.519 | GGTGATATTC | -0.481 | 0.762 | 0.694 | 0.628 |
| GGGGTTTGTC | 0.292 | 0.783 | 0.758 | 0.702 | GGGGGTGTGC | -0.481 | 0.796 | 0.758 | 0.682 |
| GTGATTTTGC | 0.290 | 0.777 | 0.686 | 0.502 | GTGGATTGCC | -0.481 | 0.750 | 0.781 | 0.779 |
| TGTGTTTCCC | 0.286 | 0.874 | 0.724 | 0.657 | GTCGATTTCC | -0.481 | 0.751 | 0.727 | 0.657 |
| GGGGCGTTTC | 0.283 | 0.784 | 0.824 | 0.653 | GGTTCATTCC | -0.481 | 0.775 | 0.664 | 0.550 |
| TTTATTTTCC | 0.279 | 0.755 | 0.559 | 0.369 | GGCATTTCCT | -0.481 | 0.790 | 0.723 | 0.607 |
| GGTGCTTTAT | 0.279 | 0.775 | 0.598 | 0.474 | GGTGGCTCGC | -0.481 | 0.761 | 0.580 | 0.577 |
| GCGTTTTCCC | 0.276 | 0.796 | 0.758 | 0.702 | GGGGGGTTCG | -0.481 | 0.778 | 0.854 | 0.681 |
| CTGGTTTTCC | 0.274 | 0.757 | 0.705 | 0.580 | GGGCTTGTAC | -0.481 | 0.761 | 0.732 | 0.599 |
| GGGATTGTCG | 0.270 | 0.820 | 0.905 | 0.643 | TGTAATTCCT | -0.481 | 0.777 | 0.619 | 0.551 |
| GGGGGCTTGC | 0.262 | 0.756 | 0.688 | 0.633 | GGTGCGTTCA | -0.481 | 0.748 | 0.715 | 0.547 |
| TGGGCTTTTC | 0.260 | 0.777 | 0.707 | 0.580 | GGTATTTGAC | -0.481 | 0.799 | 0.612 | 0.491 |
| GGGGTGTTAC | 0.255 | 0.830 | 0.822 | 0.653 | CGGATTTTCG | -0.481 | 0.780 | 0.785 | 0.536 |
| TGGAGTTCCT | 0.255 | 0.793 | 0.656 | 0.602 | GGTTCTTCAG | -0.481 | 0.748 | 0.606 | 0.469 |
| TGCATTTTCC | 0.255 | 0.772 | 0.686 | 0.502 | GGGGTTGTCG | -0.481 | 0.810 | 0.924 | 0.720 |
| TGAGTTTCCC | 0.255 | 0.827 | 0.724 | 0.657 | GGAATTGTTC | -0.481 | 0.768 | 0.679 | 0.477 |
| GGCCTTTTCC | 0.255 | 0.765 | 0.739 | 0.624 | TGGGGTTCAC | -0.481 | 0.840 | 0.657 | 0.652 |
| TTGGATTCCC | 0.255 | 0.789 | 0.746 | 0.734 | GTTAATTTCC | -0.481 | 0.847 | 0.708 | 0.579 |
| GGGGTGTCCT | 0.250 | 0.798 | 0.859 | 0.757 | GGGATGTCTC | -0.481 | 0.838 | 0.822 | 0.653 |
| TTTAATTTCC | 0.243 | 0.752 | 0.581 | 0.446 | GGGCGGTTCC | -0.481 | 0.775 | 0.789 | 0.692 |
| TGTATTGTCC | 0.243 | 0.793 | 0.679 | 0.477 | CGTAGTTCTC | -0.481 | 0.792 | 0.511 | 0.442 |
| GGGGCTATTC | 0.243 | 0.768 | 0.800 | 0.685 | GGTAGGTCTC | -0.481 | 0.808 | 0.628 | 0.515 |
| GGGACTTTGC | 0.243 | 0.861 | 0.814 | 0.635 | AGGAGTTTCT | -0.481 | 0.762 | 0.637 | 0.524 |
| GGGTGGTCTC | 0.243 | 0.753 | 0.682 | 0.637 | TGGGCTTCGC | -0.481 | 0.782 | 0.726 | 0.657 |
| GGTGGCTTTC | 0.243 | 0.756 | 0.561 | 0.500 | GGTAAGTTCT | -0.481 | 0.758 | 0.717 | 0.546 |
| GTTATGTCCC | 0.243 | 0.788 | 0.695 | 0.520 | GGCGTTTTTC | -0.481 | 0.784 | 0.705 | 0.580 |
| GGGGATTGCT | 0.243 | 0.750 | 0.799 | 0.805 | GCGATTTCCT | -0.481 | 0.769 | 0.723 | 0.607 |
| GGGATCTTCG | 0.243 | 0.780 | 0.835 | 0.594 | GGGGGTGCTA | -0.481 | 0.754 | 0.669 | 0.653 |
| GGTGGTTCTT | 0.238 | 0.783 | 0.549 | 0.546 | CGTAGTTTTC | -0.481 | 0.766 | 0.492 | 0.364 |
| GGGGTTGTTC | 0.236 | 0.826 | 0.825 | 0.687 | GGTTATTCGC | -0.481 | 0.804 | 0.654 | 0.646 |
| GGGGGTTACT | 0.233 | 0.766 | 0.710 | 0.723 | GTTTGTTCCC | -0.481 | 0.791 | 0.565 | 0.564 |
| GGGCTTTCGC | 0.233 | 0.803 | 0.758 | 0.702 | TGGAGTGTCC | -0.481 | 0.806 | 0.739 | 0.605 |
| GGCGTTTTCC | 0.230 | 0.858 | 0.831 | 0.713 | GGTATTATGC | -0.481 | 0.754 | 0.652 | 0.474 |
| TGGTTTTTAC | 0.230 | 0.756 | 0.612 | 0.491 | GGTTCTGTAC | -0.481 | 0.747 | 0.608 | 0.466 |
| TGGTCTTTCC | 0.225 | 0.784 | 0.741 | 0.624 | GTTATGTTCC | -0.481 | 0.762 | 0.676 | 0.443 |
| GGTGCTTTCA | 0.223 | 0.836 | 0.725 | 0.607 | GGTAGTTACT | -0.481 | 0.754 | 0.564 | 0.513 |
| GGAGATTTCT | 0.223 | 0.790 | 0.746 | 0.683 | GGTGTGATCC | -0.481 | 0.751 | 0.788 | 0.625 |
| GGGCCTTTTC | 0.223 | 0.780 | 0.741 | 0.624 | GGCAGTTTGC | -0.481 | 0.764 | 0.619 | 0.497 |
| CGGACTTTTC | 0.223 | 0.778 | 0.687 | 0.502 | GCGATTTTCC | -0.481 | 0.846 | 0.812 | 0.635 |
| GGGGATTCAT | 0.223 | 0.837 | 0.765 | 0.761 | GGGGGGTCGC | -0.481 | 0.799 | 0.774 | 0.725 |
| GGAAGTTTTC | 0.223 | 0.824 | 0.619 | 0.497 | GTTGTTCTCC | -0.481 | 0.751 | 0.704 | 0.571 |
| GGGTTTATAC | 0.223 | 0.747 | 0.706 | 0.596 | GGTGTCTCCG | -0.481 | 0.775 | 0.746 | 0.616 |
| GGGTGTTCAC | 0.223 | 0.868 | 0.691 | 0.697 | GGGTCTTGCC | -0.481 | 0.772 | 0.795 | 0.746 |
| GGTATCTTCC | 0.223 | 0.848 | 0.735 | 0.561 | GGTTGTTCAA | -0.481 | 0.758 | 0.456 | 0.458 |
| TGGCGTTCCC | 0.223 | 0.794 | 0.691 | 0.697 | GGGCGTGTCC | -0.481 | 0.799 | 0.792 | 0.727 |
| GGTGATTCCT | 0.223 | 0.862 | 0.765 | 0.761 | GTTGCTTCCC | -0.481 | 0.848 | 0.726 | 0.657 |
| GGTACTTCTC | 0.223 | 0.887 | 0.707 | 0.580 | GGTGTTCCGC | -0.481 | 0.785 | 0.723 | 0.649 |
| GGGGTGGTAC | 0.223 | 0.765 | 0.815 | 0.627 | GGGCGTTCGC | -0.481 | 0.794 | 0.691 | 0.697 |
| GGTTATGTCC | 0.223 | 0.809 | 0.755 | 0.676 | CGTATTTCCG | -0.481 | 0.785 | 0.677 | 0.480 |
| GTGGGCTTCC | 0.223 | 0.748 | 0.688 | 0.633 | GTTTATTCAC | -0.481 | 0.750 | 0.527 | 0.513 |
| GGGCTTTCAG | 0.223 | 0.762 | 0.731 | 0.602 | GGTATTGTCA | -0.481 | 0.800 | 0.697 | 0.504 |
| CGGGTTCTCC | 0.223 | 0.771 | 0.831 | 0.704 | GGTGCTTGAC | -0.481 | 0.771 | 0.633 | 0.569 |
| GTGCTTTCAC | 0.223 | 0.748 | 0.631 | 0.569 | TGTGGTGCAC | -0.481 | 0.754 | 0.524 | 0.494 |
| TGTACTGTCC | 0.223 | 0.775 | 0.681 | 0.477 | GGGATCTTGC | -0.481 | 0.775 | 0.735 | 0.561 |
| GGAGGTTTTC | 0.223 | 0.814 | 0.638 | 0.575 | CGGTTTTCAC | -0.481 | 0.772 | 0.631 | 0.569 |
| TGGTCTTCCC | 0.223 | 0.810 | 0.760 | 0.702 | TGTATTTTCG | -0.481 | 0.769 | 0.658 | 0.403 |
| GGTGGTCCCG | 0.223 | 0.782 | 0.756 | 0.677 | GGTGATCTCG | -0.481 | 0.762 | 0.826 | 0.681 |
| GGTGTTTCCA | 0.223 | 0.880 | 0.742 | 0.684 | GGGGTCTGCC | -0.481 | 0.752 | 0.808 | 0.760 |
| GGGGGGTTGC | 0.223 | 0.773 | 0.755 | 0.648 | TGCGGTTCCC | -0.481 | 0.780 | 0.657 | 0.652 |
| GGCTTTGCCC | 0.223 | 0.752 | 0.752 | 0.676 | GGATGTTCGC | -0.481 | 0.752 | 0.565 | 0.564 |
| GGTGGTCTAC | 0.223 | 0.799 | 0.638 | 0.566 | GGTATTTTGA | -0.481 | 0.769 | 0.577 | 0.396 |
| GGTGATTGCC | 0.223 | 0.832 | 0.781 | 0.779 | GTCAGTTTCC | -0.481 | 0.756 | 0.619 | 0.497 |
| CGTAGTTTCC | 0.223 | 0.840 | 0.619 | 0.497 | GTGGTGTCAC | -0.481 | 0.752 | 0.714 | 0.597 |
| TGGGCTGTCC | 0.223 | 0.786 | 0.827 | 0.687 | GGCGTTTTCT | -0.481 | 0.754 | 0.723 | 0.607 |
| GGTTCTTCAC | 0.223 | 0.838 | 0.633 | 0.569 | TGGAATTTCG | -0.481 | 0.786 | 0.808 | 0.612 |
| GGAGTTTCAT | 0.223 | 0.772 | 0.615 | 0.551 | TGGAGTTCGC | -0.481 | 0.802 | 0.638 | 0.575 |
| GTGGTTATCC | 0.223 | 0.757 | 0.798 | 0.685 | GGGTGTGCCC | -0.481 | 0.850 | 0.812 | 0.804 |
| GGTACTTCTT | 0.223 | 0.784 | 0.598 | 0.474 | AGTGTTTTGC | -0.481 | 0.748 | 0.578 | 0.447 |
| GGGAGTTCCG | 0.223 | 0.902 | 0.864 | 0.741 | GTGGGTTCAC | -0.481 | 0.832 | 0.657 | 0.652 |
| GTGGATTTTC | 0.217 | 0.784 | 0.727 | 0.657 | GTTGTTTTCG | -0.481 | 0.751 | 0.677 | 0.480 |
| TGTCTTTCCC | 0.217 | 0.781 | 0.631 | 0.569 | TGGAGTTTCA | -0.481 | 0.781 | 0.637 | 0.524 |
| GGGAGTTTCG | 0.212 | 0.876 | 0.845 | 0.663 | TGGCCTTTCC | -0.481 | 0.759 | 0.741 | 0.624 |
| GGTATTTTGT | 0.210 | 0.755 | 0.577 | 0.396 | GGTGTTTAAC | -0.481 | 0.810 | 0.631 | 0.569 |
| GGGTATTCTG | 0.210 | 0.757 | 0.754 | 0.679 | GGCGGTTTAC | -0.481 | 0.803 | 0.638 | 0.575 |
| GGGTGTTTCT | 0.207 | 0.786 | 0.691 | 0.646 | GCAGTTTTCC | -0.481 | 0.768 | 0.705 | 0.580 |
| GGGGTGTTCA | 0.204 | 0.787 | 0.840 | 0.680 | GGTGTTGCCA | -0.481 | 0.815 | 0.736 | 0.659 |
| TGGGTTCTCC | 0.204 | 0.780 | 0.831 | 0.704 | GTGAGTTCGC | -0.481 | 0.794 | 0.638 | 0.575 |
| GGGATTGTTA | 0.204 | 0.747 | 0.697 | 0.504 | TGGAATTTGC | -0.481 | 0.781 | 0.708 | 0.579 |
| GGGGCTTTGT | 0.199 | 0.748 | 0.725 | 0.607 | AGGGTTTCGC | -0.481 | 0.795 | 0.724 | 0.657 |
| GGTATTTTTA | 0.196 | 0.790 | 0.577 | 0.396 | TGTATTTTCA | -0.481 | 0.769 | 0.577 | 0.396 |
| GGGATGTTCG | 0.196 | 0.797 | 0.902 | 0.609 | GGTGAATTCT | -0.481 | 0.753 | 0.669 | 0.609 |
| GGGACTTTGG | 0.193 | 0.772 | 0.787 | 0.536 | ATTGGTTCCC | -0.481 | 0.757 | 0.530 | 0.519 |
| TGTATGTTCC | 0.193 | 0.770 | 0.676 | 0.443 | GGGTCTGTAC | -0.481 | 0.768 | 0.734 | 0.599 |
| GGTATTTTAG | 0.193 | 0.817 | 0.658 | 0.403 | GGTGATTACT | -0.481 | 0.750 | 0.672 | 0.672 |
| TAGGTTTTCC | 0.193 | 0.755 | 0.705 | 0.580 | GGGGTCTCTC | -0.481 | 0.812 | 0.774 | 0.715 |
| TTGGTTTTCC | 0.193 | 0.766 | 0.705 | 0.580 | GGTAATTGTC | -0.481 | 0.768 | 0.635 | 0.568 |
| TTTAGTTTCC | 0.185 | 0.747 | 0.492 | 0.364 | TTGAGTTCCC | -0.481 | 0.793 | 0.638 | 0.575 |
| GGTGTTTGCC | 0.182 | 0.835 | 0.758 | 0.702 | GGTGGTGGCC | -0.481 | 0.762 | 0.685 | 0.671 |
| GGGTCTTTTC | 0.182 | 0.806 | 0.741 | 0.624 | GGGTGTTTAG | -0.481 | 0.753 | 0.645 | 0.519 |
| TGGGTTTTAC | 0.182 | 0.823 | 0.705 | 0.580 | AGGGGTTCCT | -0.481 | 0.778 | 0.675 | 0.679 |
| GGTGTTTTGC | 0.179 | 0.848 | 0.705 | 0.580 | GGTTGTTGCC | -0.481 | 0.760 | 0.599 | 0.608 |
| GGTAATCCCG | 0.176 | 0.797 | 0.826 | 0.681 | CGGCTTTCCC | -0.481 | 0.793 | 0.758 | 0.702 |
| GGAAATTTCT | 0.176 | 0.800 | 0.726 | 0.606 | GGTTTGTCAC | -0.481 | 0.768 | 0.622 | 0.509 |
| AGGGGTTCCA | 0.176 | 0.792 | 0.675 | 0.679 | GCCGTTTCCC | -0.481 | 0.756 | 0.724 | 0.657 |
| GGGGATCCTC | 0.176 | 0.824 | 0.873 | 0.859 | TGGACTTTCG | -0.481 | 0.772 | 0.787 | 0.536 |
| GGGTCTGCCC | 0.176 | 0.841 | 0.880 | 0.809 | GGTTCTATCC | -0.481 | 0.754 | 0.708 | 0.596 |
| GGGGTGTCCG | 0.176 | 0.813 | 0.940 | 0.763 | CGGAGTTTCG | -0.481 | 0.772 | 0.718 | 0.530 |
| AGTATTCCCC | 0.176 | 0.789 | 0.704 | 0.571 | GGGGTTACGC | -0.481 | 0.791 | 0.817 | 0.762 |
| GGGTTCGCCC | 0.176 | 0.754 | 0.802 | 0.734 | GTTATTTTCA | -0.481 | 0.761 | 0.577 | 0.396 |
| AAGGTTTTCC | 0.176 | 0.750 | 0.705 | 0.580 | TGTGCTTCCA | -0.481 | 0.767 | 0.617 | 0.551 |
| AGGGCTCCCC | 0.176 | 0.782 | 0.852 | 0.782 | GGGACTTGCG | -0.481 | 0.759 | 0.840 | 0.657 |
| GGTCGTTCCC | 0.176 | 0.868 | 0.691 | 0.697 | GCGTATTTCC | -0.481 | 0.766 | 0.762 | 0.701 |
| GGGCCTTTAC | 0.176 | 0.807 | 0.741 | 0.624 | GGTGGCTTAC | -0.481 | 0.783 | 0.561 | 0.500 |
| CAGGATTCCC | 0.176 | 0.768 | 0.746 | 0.734 | TGTATATTCC | -0.481 | 0.775 | 0.609 | 0.428 |
| GTAAGTTTCC | 0.176 | 0.795 | 0.619 | 0.497 | GGTGCGTTAC | -0.481 | 0.790 | 0.697 | 0.520 |
| GACGTTTCCC | 0.176 | 0.769 | 0.724 | 0.657 | GGGAGGTTGC | -0.481 | 0.783 | 0.736 | 0.570 |
| GGAATTTCTA | 0.176 | 0.769 | 0.596 | 0.474 | GGTTATGTAC | -0.481 | 0.762 | 0.628 | 0.543 |
| GGTAGTATTC | 0.176 | 0.767 | 0.585 | 0.469 | GGAGTTGCTC | -0.481 | 0.784 | 0.717 | 0.632 |
| GGTACTCTAC | 0.176 | 0.799 | 0.687 | 0.494 | GGTCGTTCGC | -0.481 | 0.773 | 0.565 | 0.564 |
| GTGATTGCCC | 0.176 | 0.833 | 0.825 | 0.687 | GGTGCGTCGC | -0.481 | 0.768 | 0.716 | 0.597 |
| TTGAATTCCC | 0.176 | 0.799 | 0.727 | 0.657 | GGGGCTTACG | -0.481 | 0.770 | 0.860 | 0.735 |
| AGTGATTTCC | 0.176 | 0.839 | 0.727 | 0.657 | GTTGTTGTCC | -0.481 | 0.776 | 0.698 | 0.554 |
| AGTATTTCCC | 0.176 | 0.878 | 0.705 | 0.580 | TGTGTATCCC | -0.481 | 0.791 | 0.647 | 0.582 |
| GGGGGATCGC | 0.176 | 0.804 | 0.707 | 0.710 | CGAGTTTTCC | -0.481 | 0.792 | 0.705 | 0.580 |
| TGGACGTTCC | 0.176 | 0.773 | 0.805 | 0.576 | TGGGGTTCAG | -0.481 | 0.751 | 0.630 | 0.552 |
| GGAAAGTCTC | 0.176 | 0.767 | 0.718 | 0.597 | GGTCCTTTAC | -0.481 | 0.786 | 0.614 | 0.491 |
| TGGACATTCC | 0.176 | 0.778 | 0.737 | 0.561 | TGGGTTGCAC | -0.481 | 0.784 | 0.717 | 0.632 |
| CTGGATTCCC | 0.176 | 0.779 | 0.746 | 0.734 | GGAGTTGCCT | -0.481 | 0.754 | 0.736 | 0.659 |
| GTTACTCCCC | 0.176 | 0.769 | 0.706 | 0.571 | GGTGGGTCCG | -0.481 | 0.783 | 0.747 | 0.625 |
| GGGGGTACCT | 0.176 | 0.774 | 0.769 | 0.784 | AGTTATTTCC | -0.481 | 0.773 | 0.635 | 0.568 |
| GGTGTAGCCC | 0.176 | 0.821 | 0.767 | 0.690 | GGGAGTGTCT | -0.481 | 0.798 | 0.757 | 0.632 |
| GGTTATGCCC | 0.176 | 0.834 | 0.774 | 0.753 | GTTAGTTCGC | -0.481 | 0.773 | 0.511 | 0.442 |
| GGGGATGCAA | 0.176 | 0.787 | 0.758 | 0.735 | GAGATTTTCG | -0.481 | 0.771 | 0.785 | 0.536 |
| GGCAGTTCTC | 0.176 | 0.811 | 0.638 | 0.575 | GGTATATTCG | -0.481 | 0.781 | 0.708 | 0.461 |
| GGGTATATCC | 0.176 | 0.790 | 0.855 | 0.806 | CGGACTTTCT | -0.481 | 0.748 | 0.706 | 0.529 |
| GGGAATTCGC | 0.176 | 0.902 | 0.854 | 0.790 | GGGGCTGTCT | -0.481 | 0.778 | 0.845 | 0.714 |
| GGATCTTTAC | 0.176 | 0.765 | 0.614 | 0.491 | GATAGTTTTC | -0.481 | 0.756 | 0.492 | 0.364 |
| GGGCATCTCC | 0.176 | 0.780 | 0.888 | 0.826 | GTTGCTTCAC | -0.481 | 0.801 | 0.599 | 0.524 |
| GGAGCTTTCC | 0.176 | 0.878 | 0.833 | 0.713 | GGTGTGGCCC | -0.481 | 0.816 | 0.834 | 0.705 |
| GGGGCGTTAC | 0.176 | 0.811 | 0.824 | 0.653 | GATGCTTCTC | -0.481 | 0.763 | 0.599 | 0.524 |
| GGGGCCCTCC | 0.176 | 0.752 | 0.883 | 0.763 | TGGGTTTTCG | -0.481 | 0.780 | 0.804 | 0.613 |
| TGGAGATTCC | 0.176 | 0.787 | 0.669 | 0.556 | GGTGGTACCT | -0.481 | 0.753 | 0.642 | 0.651 |
| GGTGGTACCC | 0.176 | 0.856 | 0.750 | 0.757 | GGTTGTTTCA | -0.495 | 0.779 | 0.564 | 0.513 |
| GTGGGATCCC | 0.176 | 0.795 | 0.707 | 0.710 | GGTTGTTTCG | -0.495 | 0.779 | 0.645 | 0.519 |
| GGGAAGTTCG | 0.176 | 0.793 | 0.925 | 0.685 | GGTTGTTCGC | -0.495 | 0.799 | 0.565 | 0.564 |
| TAGGATTTCC | 0.176 | 0.751 | 0.727 | 0.657 | GGTTGTGTCC | -0.495 | 0.803 | 0.666 | 0.594 |
| AGGCTTTCCC | 0.176 | 0.797 | 0.758 | 0.702 | GGTGGGTTGC | -0.495 | 0.752 | 0.628 | 0.515 |
| GCTACTTTCC | 0.176 | 0.807 | 0.687 | 0.502 | TGTGATTTTC | -0.495 | 0.771 | 0.601 | 0.524 |
| GGTTATACCC | 0.176 | 0.795 | 0.747 | 0.750 | GGTTGTTTGC | -0.495 | 0.773 | 0.545 | 0.486 |
| TGTGCTCCCC | 0.176 | 0.767 | 0.725 | 0.649 | CGGGTTTTGC | -0.495 | 0.765 | 0.705 | 0.580 |
| GGTGAGGTCC | 0.176 | 0.787 | 0.838 | 0.704 | GTTGTGTCCC | -0.509 | 0.778 | 0.714 | 0.597 |
| GTTAGTCCCC | 0.176 | 0.779 | 0.638 | 0.566 | GGTATTTGCG | -0.509 | 0.756 | 0.712 | 0.524 |
| GGGCTATTCC | 0.176 | 0.789 | 0.789 | 0.683 | GTTGTTTCTC | -0.509 | 0.792 | 0.597 | 0.524 |
| GGGACATTAC | 0.176 | 0.826 | 0.737 | 0.561 | GGGTGTTCGC | -0.509 | 0.820 | 0.691 | 0.697 |
| GGGAGTGCCG | 0.176 | 0.837 | 0.858 | 0.715 | GGGTGTTCTG | -0.509 | 0.752 | 0.664 | 0.597 |
| GGGACTATCG | 0.176 | 0.763 | 0.880 | 0.640 | GGCGTTTCCT | -0.523 | 0.780 | 0.742 | 0.684 |
| GGGATATCCG | 0.176 | 0.827 | 0.854 | 0.671 | GGGTGTGCCT | -0.523 | 0.747 | 0.703 | 0.698 |
| GGAATTTCTC | 0.176 | 0.858 | 0.705 | 0.580 | GGGTGTTCAT | -0.523 | 0.765 | 0.583 | 0.591 |
| GGGATTCCAG | 0.176 | 0.775 | 0.804 | 0.604 | GTGGTTGCTC | -0.523 | 0.749 | 0.717 | 0.632 |
| AGGGGTCTCC | 0.176 | 0.766 | 0.764 | 0.699 | TGGGCTTTCT | -0.523 | 0.748 | 0.725 | 0.607 |
| GGACATTTCC | 0.176 | 0.801 | 0.762 | 0.701 | GGCGTTTCTC | -0.523 | 0.810 | 0.724 | 0.657 |
| GGTTACTCCC | 0.176 | 0.794 | 0.704 | 0.704 | GGTGAGTTTC | -0.523 | 0.778 | 0.718 | 0.597 |
| TTGATTTCAC | 0.176 | 0.755 | 0.578 | 0.447 | TGTATTTCCG | -0.523 | 0.794 | 0.677 | 0.480 |
| GGGGCTTCCA | 0.176 | 0.883 | 0.871 | 0.817 | GGGTCTTTGC | -0.523 | 0.785 | 0.741 | 0.624 |
| TGGAAGTCCC | 0.176 | 0.813 | 0.844 | 0.730 | GTGGGTTTCA | -0.523 | 0.764 | 0.656 | 0.602 |
| GGGGGTACAC | 0.176 | 0.831 | 0.750 | 0.757 | GGTGTCTCTC | -0.523 | 0.791 | 0.647 | 0.582 |
| GGGGTTATCA | 0.176 | 0.771 | 0.816 | 0.711 | GTTGGTGTCC | -0.523 | 0.767 | 0.631 | 0.549 |
| GGGACATTTC | 0.176 | 0.799 | 0.737 | 0.561 | GTTGGTTCCT | -0.523 | 0.754 | 0.549 | 0.546 |
| GGTACTGTAC | 0.176 | 0.824 | 0.681 | 0.477 | TGTGCTTCCT | -0.523 | 0.752 | 0.617 | 0.551 |
| TGGATTACCC | 0.176 | 0.801 | 0.798 | 0.685 | TGGGCTTCTC | -0.523 | 0.803 | 0.726 | 0.657 |
| GGAATGTCCG | 0.176 | 0.755 | 0.794 | 0.553 | GGTGTTGTCT | -0.523 | 0.775 | 0.716 | 0.581 |
| GGCTATTCCC | 0.176 | 0.813 | 0.781 | 0.779 | GGTGTGTCCT | -0.523 | 0.777 | 0.732 | 0.624 |
| GGGATTGCCG | 0.176 | 0.846 | 0.924 | 0.720 | GGGGTTGCAT | -0.538 | 0.776 | 0.736 | 0.659 |
| TGAAGTTTCC | 0.176 | 0.803 | 0.619 | 0.497 | GTGGGTTCTC | -0.538 | 0.805 | 0.657 | 0.652 |
| GGGAATTCTA | 0.176 | 0.834 | 0.746 | 0.683 | TGTAATTTTC | -0.538 | 0.781 | 0.581 | 0.446 |
| CCGATTTCCC | 0.176 | 0.768 | 0.705 | 0.580 | TGGGCTTCCG | -0.538 | 0.787 | 0.825 | 0.690 |
| GCGGGATCCC | 0.176 | 0.770 | 0.707 | 0.710 | TGTCGTTTCC | -0.538 | 0.747 | 0.545 | 0.486 |
| CGGTCTTTCC | 0.176 | 0.775 | 0.741 | 0.624 | GGGGTTGCCG | -0.538 | 0.836 | 0.944 | 0.798 |
| TGGGATATCC | 0.176 | 0.762 | 0.821 | 0.761 | GGTAGTTCGG | -0.538 | 0.786 | 0.611 | 0.475 |
| GGAATTTGCC | 0.176 | 0.799 | 0.739 | 0.624 | GGGTTTGCGC | -0.538 | 0.764 | 0.752 | 0.676 |
| TGGGATGCCC | 0.176 | 0.827 | 0.867 | 0.842 | GGTGGTGTCT | -0.538 | 0.767 | 0.650 | 0.576 |
| TGGGGTCCCC | 0.176 | 0.797 | 0.783 | 0.777 | GGTGGTTACG | -0.538 | 0.758 | 0.664 | 0.597 |
| GGAGTTGCCC | 0.176 | 0.858 | 0.844 | 0.765 | GGGTCTTCCG | -0.538 | 0.816 | 0.860 | 0.735 |
| TGTGGATCCC | 0.176 | 0.782 | 0.580 | 0.577 | GTTAGTTTGC | -0.538 | 0.747 | 0.492 | 0.364 |
| TGGGTGTCCC | 0.176 | 0.807 | 0.841 | 0.730 | CGGGCTTTCG | -0.538 | 0.752 | 0.806 | 0.613 |
| TGAGATTTCC | 0.176 | 0.798 | 0.727 | 0.657 | TGTGCTTTTC | -0.538 | 0.756 | 0.580 | 0.447 |
| GGGAGTACAC | 0.176 | 0.841 | 0.731 | 0.679 | AGTGTTTCTC | -0.538 | 0.795 | 0.597 | 0.524 |
| TAGGGTTCCC | 0.176 | 0.772 | 0.657 | 0.652 | TGTATTTTTC | -0.553 | 0.784 | 0.559 | 0.369 |
| GGTAATTCTC | 0.176 | 0.902 | 0.727 | 0.657 | GGTGTGTTCT | -0.553 | 0.752 | 0.713 | 0.547 |
| GGATGGTCCC | 0.176 | 0.759 | 0.682 | 0.637 | GTTGCTTCTC | -0.553 | 0.774 | 0.599 | 0.524 |
| CGAGGTTTCC | 0.176 | 0.783 | 0.638 | 0.575 | GTGAGTTTCG | -0.569 | 0.773 | 0.718 | 0.530 |
| GGGATATTGC | 0.176 | 0.796 | 0.735 | 0.561 | GGTGTTGTGC | -0.569 | 0.784 | 0.698 | 0.554 |
| GGTTCTTACC | 0.176 | 0.772 | 0.668 | 0.613 | GGTGTTTCGG | -0.569 | 0.785 | 0.697 | 0.557 |
| GGGGCTTCTA | 0.176 | 0.809 | 0.744 | 0.684 | GGTGTTGGCC | -0.569 | 0.771 | 0.752 | 0.676 |
| GGGGTTACCG | 0.176 | 0.797 | 0.917 | 0.795 | GGCGGTTTCG | -0.569 | 0.760 | 0.737 | 0.608 |
| GGGGCTCACC | 0.176 | 0.770 | 0.887 | 0.826 | GGTGATGTTC | -0.569 | 0.802 | 0.721 | 0.631 |
| GGTGTGGCAC | 0.176 | 0.770 | 0.708 | 0.572 | GGTGTTCCTC | -0.569 | 0.806 | 0.723 | 0.649 |
| GGGGAGTCCT | 0.176 | 0.795 | 0.882 | 0.834 | GGTCGTTTTC | -0.569 | 0.768 | 0.545 | 0.486 |
| GGGATTATGC | 0.176 | 0.775 | 0.779 | 0.607 | TGGGTTTCAG | -0.569 | 0.759 | 0.697 | 0.557 |
| GGTGGCGCCC | 0.176 | 0.791 | 0.700 | 0.685 | GGGTGTGTTC | -0.569 | 0.751 | 0.666 | 0.594 |
| GGTAAGTTCC | 0.176 | 0.861 | 0.825 | 0.652 | GGTGGGTCTC | -0.569 | 0.798 | 0.647 | 0.592 |
| GGGAAGTTCT | 0.176 | 0.779 | 0.844 | 0.679 | GGTGTGTCCG | -0.569 | 0.791 | 0.814 | 0.630 |
| GGTAGGTCCC | 0.176 | 0.882 | 0.755 | 0.648 | GGTTGCTTCC | -0.569 | 0.763 | 0.595 | 0.544 |
| GGGATCTTCA | 0.176 | 0.781 | 0.754 | 0.587 | GCGGTTTTCG | -0.569 | 0.747 | 0.804 | 0.613 |
| GGTACTTGCC | 0.176 | 0.827 | 0.741 | 0.624 | GGTATTTGGC | -0.569 | 0.751 | 0.612 | 0.491 |
| GGGCATTCCG | 0.176 | 0.805 | 0.880 | 0.812 | GGTGATTTGG | -0.585 | 0.756 | 0.700 | 0.557 |
| GGGGTATCGC | 0.176 | 0.812 | 0.774 | 0.715 | GTGGCTTTTC | -0.585 | 0.770 | 0.707 | 0.580 |
| GGGATTATCG | 0.176 | 0.781 | 0.878 | 0.640 | GGGCGTTTCT | -0.585 | 0.760 | 0.691 | 0.646 |
| GGTATTATCC | 0.176 | 0.849 | 0.779 | 0.607 | GTTTGTTTCC | -0.585 | 0.765 | 0.545 | 0.486 |
| GCGAGTTCCC | 0.176 | 0.863 | 0.765 | 0.708 | GGTGTTGTCG | -0.602 | 0.789 | 0.798 | 0.587 |
| GGTTCATCCC | 0.176 | 0.801 | 0.683 | 0.627 | GGGCGTTCCG | -0.602 | 0.800 | 0.791 | 0.730 |
| GGTTTATTAC | 0.176 | 0.747 | 0.535 | 0.417 | TGGAGTTCCG | -0.602 | 0.807 | 0.737 | 0.608 |
| GGGAGTATTC | 0.176 | 0.788 | 0.712 | 0.602 | GGGGGGTCCG | -0.602 | 0.804 | 0.874 | 0.758 |
| GGTAGTGTAC | 0.176 | 0.833 | 0.612 | 0.472 | TGGATTTCAT | -0.602 | 0.755 | 0.596 | 0.474 |
| GACATTTCCC | 0.176 | 0.779 | 0.705 | 0.580 | AGTGTTTCGC | -0.602 | 0.774 | 0.597 | 0.524 |
| GGAACTTCTC | 0.176 | 0.840 | 0.707 | 0.580 | GGTTGTGCTC | -0.602 | 0.755 | 0.558 | 0.538 |
| GGATGTTTAC | 0.176 | 0.775 | 0.545 | 0.486 | GGTGATTCCG | -0.602 | 0.876 | 0.846 | 0.767 |
| TGGTGTTTAC | 0.170 | 0.747 | 0.545 | 0.486 | CGGGTTTTCG | -0.602 | 0.770 | 0.804 | 0.613 |
| GGGATTGTCT | 0.164 | 0.806 | 0.824 | 0.637 | GGTACTGTCG | -0.602 | 0.781 | 0.780 | 0.510 |
| GGTTCTTTAC | 0.158 | 0.812 | 0.614 | 0.491 | TGTGATTTAC | -0.602 | 0.798 | 0.601 | 0.524 |
| GGGGGTATCT | 0.155 | 0.748 | 0.750 | 0.706 | CTTATTTCCC | -0.602 | 0.772 | 0.578 | 0.447 |
| TGTGCTTCCC | 0.155 | 0.856 | 0.726 | 0.657 | GGTGGTCCGC | -0.602 | 0.777 | 0.657 | 0.644 |
| TGTTATTCCC | 0.155 | 0.804 | 0.654 | 0.646 | GGTCCTTCCG | -0.602 | 0.769 | 0.733 | 0.602 |
| GCGGTTTTTC | 0.152 | 0.763 | 0.705 | 0.580 | GTGCGTTTCC | -0.602 | 0.760 | 0.672 | 0.619 |
| GGTATTTTGG | 0.149 | 0.769 | 0.658 | 0.403 | TGGTATTTAC | -0.602 | 0.753 | 0.635 | 0.568 |
| GTGAGTTTCC | 0.146 | 0.863 | 0.746 | 0.630 | TGTGATTTCA | -0.602 | 0.756 | 0.619 | 0.551 |
| GGTGGGTTCC | 0.146 | 0.846 | 0.755 | 0.648 | GCTGTTTCCC | -0.602 | 0.841 | 0.724 | 0.657 |
| GGGTATTTAG | 0.146 | 0.758 | 0.734 | 0.601 | GGTGTATTCG | -0.602 | 0.771 | 0.727 | 0.538 |
| GGGATTGTAT | 0.146 | 0.760 | 0.697 | 0.504 | GGGGGTTGAC | -0.602 | 0.801 | 0.691 | 0.697 |
| GGATGTTTCC | 0.146 | 0.821 | 0.672 | 0.619 | GGGTGTTTAA | -0.602 | 0.753 | 0.564 | 0.513 |
| GGTGCATTTC | 0.146 | 0.768 | 0.630 | 0.505 | TGTATGTCCC | -0.602 | 0.796 | 0.695 | 0.520 |
| TGTGTATTCC | 0.146 | 0.765 | 0.628 | 0.505 | GCGGTTTTCA | -0.602 | 0.747 | 0.723 | 0.607 |
| CGGGTTTGCC | 0.146 | 0.752 | 0.758 | 0.702 | GGGGTTGCAG | -0.602 | 0.790 | 0.817 | 0.665 |
| GGGGCTTCCG | 0.146 | 0.883 | 0.952 | 0.823 | AGTAGTTTTC | -0.602 | 0.770 | 0.492 | 0.364 |
| GGGATTTGCT | 0.140 | 0.763 | 0.757 | 0.651 | GGGTATGCTC | -0.602 | 0.782 | 0.774 | 0.753 |
| GGGGTTTCGA | 0.140 | 0.806 | 0.742 | 0.684 | TGGAGTTTCG | -0.602 | 0.781 | 0.718 | 0.530 |
| GTGGATTTCT | 0.137 | 0.755 | 0.746 | 0.683 | GGGACTGTCT | -0.602 | 0.788 | 0.826 | 0.637 |
| GGTGTATTTC | 0.137 | 0.786 | 0.628 | 0.505 | AGTGATTTTC | -0.602 | 0.766 | 0.601 | 0.524 |
| GGTGTTGTAC | 0.134 | 0.832 | 0.698 | 0.554 | GTGGGTTCCG | -0.602 | 0.789 | 0.757 | 0.685 |
| TGTTCTTCCC | 0.134 | 0.789 | 0.633 | 0.569 | GTTATTCTCC | -0.602 | 0.761 | 0.685 | 0.494 |
| GGTGGTTCTA | 0.134 | 0.798 | 0.549 | 0.546 | GGTGCTGCGC | -0.602 | 0.791 | 0.719 | 0.632 |
| GGTAGTTTTG | 0.130 | 0.781 | 0.591 | 0.397 | GGGAGTTCGG | -0.602 | 0.807 | 0.737 | 0.608 |
| GGTTTCTCAC | 0.124 | 0.751 | 0.555 | 0.494 | GGGGGTATGC | -0.602 | 0.757 | 0.731 | 0.679 |
| GGTCTCTCCC | 0.124 | 0.772 | 0.681 | 0.627 | AGTGTTGTCC | -0.602 | 0.778 | 0.698 | 0.554 |
| GGTATTCCTC | 0.124 | 0.816 | 0.704 | 0.571 | GGGGCTCTGC | -0.602 | 0.762 | 0.833 | 0.704 |
| GGGGTTACCT | 0.124 | 0.783 | 0.836 | 0.789 | GTGATTTTCA | -0.602 | 0.782 | 0.704 | 0.529 |
| AGGGTTTGCC | 0.124 | 0.756 | 0.758 | 0.702 | AGTGGTTCTC | -0.602 | 0.786 | 0.530 | 0.519 |
| GGAAGTTTCT | 0.124 | 0.794 | 0.637 | 0.524 | GGTGTTGCTC | -0.602 | 0.831 | 0.717 | 0.632 |
| GTTATTGCCC | 0.124 | 0.811 | 0.698 | 0.554 | GGGGTATTCG | -0.602 | 0.792 | 0.854 | 0.671 |
| TGTGTTCCCC | 0.124 | 0.785 | 0.723 | 0.649 | TGGATTTTAC | -0.602 | 0.833 | 0.686 | 0.502 |
| GGGTGTTCCA | 0.124 | 0.826 | 0.710 | 0.723 | GGGTATTCCG | -0.602 | 0.831 | 0.880 | 0.812 |
| GGAGATTTTC | 0.124 | 0.819 | 0.727 | 0.657 | GGGTTATCCG | -0.602 | 0.751 | 0.781 | 0.660 |
| CGGGATTTCT | 0.124 | 0.753 | 0.746 | 0.683 | GGTGCTTCGA | -0.602 | 0.767 | 0.617 | 0.551 |
| GGATTTGTCC | 0.124 | 0.765 | 0.732 | 0.599 | GGGGCTGTCG | -0.602 | 0.792 | 0.926 | 0.720 |
| TGGACTTCCT | 0.124 | 0.783 | 0.725 | 0.607 | GATGGTTCTC | -0.602 | 0.772 | 0.530 | 0.519 |
| TGTGATTCGC | 0.124 | 0.776 | 0.620 | 0.601 | AGGTTTTTAC | -0.602 | 0.751 | 0.612 | 0.491 |
| AGGTCTTTCC | 0.124 | 0.779 | 0.741 | 0.624 | GGTGCTGCCG | -0.602 | 0.797 | 0.819 | 0.665 |
| GGAATTTCGG | 0.124 | 0.748 | 0.677 | 0.480 | GTTGCTGTCC | -0.602 | 0.758 | 0.700 | 0.554 |
| GGTGCGGTCC | 0.124 | 0.772 | 0.817 | 0.627 | GGGGTTCTCA | -0.602 | 0.786 | 0.849 | 0.731 |
| GTGGGGTCCC | 0.124 | 0.790 | 0.774 | 0.725 | TGTAATTTGC | -0.602 | 0.760 | 0.581 | 0.446 |
| CGATTTTCCC | 0.124 | 0.751 | 0.631 | 0.569 | TGGGGTGTAC | -0.602 | 0.749 | 0.631 | 0.549 |
| TGCAGTTTCC | 0.124 | 0.764 | 0.619 | 0.497 | TGAGTTTTAC | -0.602 | 0.755 | 0.578 | 0.447 |
| GGGGTATTGC | 0.124 | 0.786 | 0.755 | 0.638 | TGTAGTTTAC | -0.602 | 0.803 | 0.492 | 0.364 |
| GGGGCGTCCT | 0.124 | 0.780 | 0.861 | 0.757 | GGGGGTGTCG | -0.602 | 0.802 | 0.858 | 0.715 |
| GGTGTTGCAC | 0.124 | 0.858 | 0.717 | 0.632 | CGGGGTTCGC | -0.602 | 0.782 | 0.657 | 0.652 |
| GGGAGTCTTC | 0.124 | 0.803 | 0.745 | 0.622 | GGGGTCTCGC | -0.602 | 0.791 | 0.774 | 0.715 |
| GCTGTTTCCA | 0.124 | 0.752 | 0.615 | 0.551 | GGGGCTTACT | -0.602 | 0.756 | 0.778 | 0.729 |
| GTGCATTTCC | 0.124 | 0.766 | 0.762 | 0.701 | GAGATTTTTC | -0.602 | 0.786 | 0.686 | 0.502 |
| GGCTGTTCCC | 0.124 | 0.808 | 0.691 | 0.697 | GGTGCATTCG | -0.602 | 0.752 | 0.729 | 0.538 |
| GGGCATTTCT | 0.124 | 0.765 | 0.780 | 0.728 | GATGTTTCGC | -0.602 | 0.760 | 0.597 | 0.524 |
| GGGTTTGCAC | 0.124 | 0.812 | 0.752 | 0.676 | GGTAGTTCTG | -0.602 | 0.807 | 0.611 | 0.475 |
| GGGGTTTGCA | 0.111 | 0.768 | 0.776 | 0.729 | GGTGGTTCGT | -0.602 | 0.762 | 0.549 | 0.546 |
| GGGATTTGGC | 0.111 | 0.772 | 0.739 | 0.624 | GTTGGTTTTC | -0.602 | 0.758 | 0.511 | 0.442 |
| GGGGCTCTTC | 0.111 | 0.783 | 0.833 | 0.704 | GGTGTTACGC | -0.602 | 0.770 | 0.690 | 0.629 |
| GTGGTTGTCC | 0.107 | 0.797 | 0.825 | 0.687 | AGTATTTTGC | -0.602 | 0.758 | 0.559 | 0.369 |
| TGGGATTTCT | 0.107 | 0.762 | 0.746 | 0.683 | GTGATTTCAT | -0.602 | 0.747 | 0.596 | 0.474 |
| GGTGCTTTCG | 0.104 | 0.836 | 0.806 | 0.613 | GCTATTTTCC | -0.602 | 0.825 | 0.686 | 0.502 |
| GGGTGTTGCC | 0.104 | 0.781 | 0.726 | 0.741 | CGGGGTTCCG | -0.602 | 0.788 | 0.757 | 0.685 |
| GTGGTTTCAG | 0.104 | 0.751 | 0.697 | 0.557 | TGGACTTTCT | -0.602 | 0.757 | 0.706 | 0.529 |
| TGTGATTTCC | 0.104 | 0.845 | 0.727 | 0.657 | GGTTGTTCTC | -0.620 | 0.820 | 0.565 | 0.564 |
| GGTGTTTCTT | 0.097 | 0.792 | 0.615 | 0.551 | AGTGTTTTCG | -0.620 | 0.753 | 0.677 | 0.480 |
| TGGAATTTTC | 0.097 | 0.802 | 0.708 | 0.579 | GGTGGTGTGC | -0.620 | 0.775 | 0.631 | 0.549 |
| TGGGTCTCCC | 0.097 | 0.790 | 0.774 | 0.715 | GGTTGTTCCG | -0.620 | 0.804 | 0.664 | 0.597 |
| GTGTGTTCCC | 0.097 | 0.812 | 0.691 | 0.697 | GGTGTTCTGC | -0.638 | 0.760 | 0.704 | 0.571 |
| GGGGATTGCG | 0.097 | 0.764 | 0.880 | 0.812 | GGGTATGTTC | -0.638 | 0.756 | 0.755 | 0.676 |
| GTGGATTCCG | 0.097 | 0.795 | 0.846 | 0.767 | GGGTTTGCCG | -0.638 | 0.770 | 0.851 | 0.709 |
| GGTGGTATTC | 0.097 | 0.757 | 0.605 | 0.546 | GGCGTTTTGC | -0.638 | 0.763 | 0.705 | 0.580 |
| GGGGATGTCT | 0.097 | 0.793 | 0.866 | 0.791 | GGTAGTTCTT | -0.638 | 0.793 | 0.529 | 0.469 |
| GCTATTTCCT | 0.097 | 0.748 | 0.596 | 0.474 | GGTATGTTCG | -0.638 | 0.776 | 0.775 | 0.476 |
| GGGGTGTCAT | 0.097 | 0.752 | 0.732 | 0.624 | GTTGTATTCC | -0.658 | 0.757 | 0.628 | 0.505 |
| GGTATTTCTA | 0.097 | 0.816 | 0.596 | 0.474 | GGGGGTTGCG | -0.658 | 0.759 | 0.791 | 0.730 |
| TAGGTTTCCC | 0.097 | 0.781 | 0.724 | 0.657 | GTGGCTTCCT | -0.658 | 0.766 | 0.744 | 0.684 |
| TGGGTTTTCA | 0.097 | 0.780 | 0.723 | 0.607 | TGTGTTTCAC | -0.658 | 0.827 | 0.597 | 0.524 |
| TGTATTTGCC | 0.097 | 0.750 | 0.612 | 0.491 | GGTGTGTTCG | -0.658 | 0.766 | 0.794 | 0.553 |
| GGATATTTTC | 0.097 | 0.753 | 0.635 | 0.568 | GTTATTTCGC | -0.658 | 0.781 | 0.578 | 0.447 |
| GGGATTGCCT | 0.097 | 0.832 | 0.843 | 0.714 | GCTGGTTCTC | -0.658 | 0.759 | 0.530 | 0.519 |
| GGAGTTTTAG | 0.097 | 0.761 | 0.677 | 0.480 | GGGTGTGCTC | -0.658 | 0.777 | 0.685 | 0.671 |
| GGTGGGTTTC | 0.097 | 0.773 | 0.628 | 0.515 | GTGACTTTCT | -0.658 | 0.750 | 0.706 | 0.529 |
| GGGTCTCTCC | 0.097 | 0.791 | 0.867 | 0.749 | GCGGTTTCCT | -0.658 | 0.759 | 0.742 | 0.684 |
| GGGGTTCTCG | 0.093 | 0.786 | 0.931 | 0.737 | GGTGCGTTTC | -0.678 | 0.763 | 0.697 | 0.520 |
| GGTGCTTCTT | 0.090 | 0.774 | 0.617 | 0.551 | GCTATTTTTC | -0.678 | 0.751 | 0.559 | 0.369 |
| GTTATTGTCC | 0.086 | 0.786 | 0.679 | 0.477 | GGGGTTGCTC | -0.678 | 0.852 | 0.844 | 0.765 |
| GGTGTTTCTA | 0.086 | 0.806 | 0.615 | 0.551 | GGTGCTGTCG | -0.678 | 0.771 | 0.800 | 0.587 |
| GGTATTCTTC | 0.086 | 0.790 | 0.685 | 0.494 | GGTGGTTCGG | -0.678 | 0.776 | 0.630 | 0.552 |
| TGGGTTTCTC | 0.083 | 0.821 | 0.724 | 0.657 | GTTGTGTTCC | -0.678 | 0.752 | 0.695 | 0.520 |
| GGTGTTACCT | 0.079 | 0.762 | 0.709 | 0.656 | GTGGGTTTGC | -0.678 | 0.758 | 0.638 | 0.575 |
| TGGGTTGCCC | 0.079 | 0.830 | 0.844 | 0.765 | GTTGTTGCCC | -0.699 | 0.802 | 0.717 | 0.632 |
| GCGGTTTCCC | 0.079 | 0.862 | 0.851 | 0.790 | GGCGATTTTC | -0.699 | 0.781 | 0.727 | 0.657 |
| GGTAGTTCTC | 0.079 | 0.897 | 0.638 | 0.575 | GGTAGGTTCG | -0.699 | 0.767 | 0.709 | 0.471 |
| TCGATTTTCC | 0.079 | 0.751 | 0.686 | 0.502 | GGTTCCTTCC | -0.699 | 0.754 | 0.664 | 0.550 |
| TGTGGTTCAC | 0.079 | 0.819 | 0.530 | 0.519 | GGCAGTTTTC | -0.699 | 0.785 | 0.619 | 0.497 |
| TTGACTTTCC | 0.079 | 0.758 | 0.687 | 0.502 | GGTGTGCTCC | -0.699 | 0.766 | 0.821 | 0.645 |
| GGTGACTTTC | 0.079 | 0.761 | 0.650 | 0.582 | GGTTATTTCA | -0.699 | 0.784 | 0.653 | 0.595 |
| GGTAATTTTG | 0.079 | 0.787 | 0.681 | 0.479 | GGGACTTCGT | -0.699 | 0.784 | 0.725 | 0.607 |
| TGGTTTGCCC | 0.079 | 0.764 | 0.752 | 0.676 | GGTACTTCGG | -0.699 | 0.777 | 0.679 | 0.480 |
| GGTGTTTCCG | 0.079 | 0.880 | 0.823 | 0.690 | GGTCGTTTAC | -0.699 | 0.796 | 0.545 | 0.486 |
| GTGGTCTTCC | 0.079 | 0.757 | 0.755 | 0.638 | GGTAGTTTAG | -0.699 | 0.809 | 0.591 | 0.397 |
| GTTGTTTTCC | 0.072 | 0.840 | 0.705 | 0.580 | GTTGATGTCC | -0.699 | 0.772 | 0.721 | 0.631 |
| TGGGTTGTCC | 0.072 | 0.805 | 0.825 | 0.687 | GGTGCTTATC | -0.699 | 0.764 | 0.633 | 0.569 |
| GTGTCTTTCC | 0.072 | 0.777 | 0.741 | 0.624 | GCTGTTTCCG | -0.699 | 0.752 | 0.697 | 0.557 |
| CGGGTTTCTC | 0.072 | 0.812 | 0.724 | 0.657 | GTTGTTGCAC | -0.699 | 0.755 | 0.590 | 0.499 |
| GGGGTGTTCT | 0.072 | 0.773 | 0.840 | 0.680 | GGGGCTTCGG | -0.699 | 0.788 | 0.825 | 0.690 |
| GGGATTGTTC | 0.072 | 0.836 | 0.806 | 0.610 | GGTGATTTAG | -0.699 | 0.804 | 0.700 | 0.557 |
| GGGGCTTCTG | 0.072 | 0.809 | 0.825 | 0.690 | GTTACTTTCC | -0.699 | 0.832 | 0.687 | 0.502 |
| TGTTTTTCAC | 0.072 | 0.761 | 0.505 | 0.436 | GGTGTGTTAC | -0.699 | 0.808 | 0.695 | 0.520 |
| TGGGTTTACC | 0.068 | 0.782 | 0.758 | 0.702 | CGGGTTGCTC | -0.699 | 0.747 | 0.717 | 0.632 |
| GGGATTGTGC | 0.068 | 0.815 | 0.806 | 0.610 | GGTAGTTTGA | -0.699 | 0.761 | 0.510 | 0.391 |
| GGTGCTTGCC | 0.068 | 0.817 | 0.760 | 0.702 | CGTGGTTTAC | -0.699 | 0.784 | 0.511 | 0.442 |
| TGGATTTCGC | 0.068 | 0.810 | 0.705 | 0.580 | GATGTTTCCG | -0.699 | 0.765 | 0.697 | 0.557 |
| GGGAGTTCTT | 0.068 | 0.814 | 0.656 | 0.602 | GTGAGTTCTC | -0.699 | 0.815 | 0.638 | 0.575 |
| GGTATTTCTT | 0.068 | 0.802 | 0.596 | 0.474 | GTGTGTTCAC | -0.699 | 0.765 | 0.565 | 0.564 |
| TGTGGATTCC | 0.068 | 0.756 | 0.561 | 0.500 | GCGGTTTCGC | -0.699 | 0.767 | 0.724 | 0.657 |
| GGGCTGTTCC | 0.068 | 0.784 | 0.856 | 0.698 | GTGGATTTGC | -0.699 | 0.763 | 0.727 | 0.657 |
| TTGGTTTCCC | 0.068 | 0.792 | 0.724 | 0.657 | GGCGGTTCCT | -0.699 | 0.771 | 0.675 | 0.679 |
| GGGAGTTGTC | 0.068 | 0.784 | 0.672 | 0.619 | CGGAGTTTTC | -0.699 | 0.787 | 0.619 | 0.497 |
| GGTGGTTTGC | 0.064 | 0.840 | 0.638 | 0.575 | GGTGATGTGC | -0.699 | 0.780 | 0.721 | 0.631 |
| GGGGTTATCT | 0.061 | 0.757 | 0.816 | 0.711 | GGTGAGTTGC | -0.699 | 0.757 | 0.718 | 0.597 |
| GGTGATTCTT | 0.061 | 0.788 | 0.638 | 0.628 | GTCATTTTCC | -0.699 | 0.765 | 0.686 | 0.502 |
| TGTGATTCCG | 0.057 | 0.781 | 0.719 | 0.634 | GGTGAGTTCT | -0.699 | 0.748 | 0.736 | 0.624 |
| GGGTATTTGC | 0.057 | 0.800 | 0.762 | 0.701 | GGTTGTTCAC | -0.699 | 0.847 | 0.565 | 0.564 |
| GGGGGTTTGG | 0.057 | 0.772 | 0.737 | 0.608 | GTGGCGTTCC | -0.699 | 0.755 | 0.824 | 0.653 |
| GGGGTTGGCC | 0.057 | 0.792 | 0.878 | 0.809 | GCTTGTTCCC | -0.699 | 0.766 | 0.565 | 0.564 |
| GGTACTTCTG | 0.057 | 0.798 | 0.679 | 0.480 | GGGGAGTTCG | -0.699 | 0.783 | 0.944 | 0.763 |
| TGTAGTTCTC | 0.053 | 0.801 | 0.511 | 0.442 | GCTGCTTCTC | -0.699 | 0.749 | 0.599 | 0.524 |
| TGTATTTTCC | 0.049 | 0.858 | 0.686 | 0.502 | GTGAGTTCCT | -0.699 | 0.785 | 0.656 | 0.602 |
| GTGGTTTCGC | 0.049 | 0.793 | 0.724 | 0.657 | GGTTGTTACC | -0.699 | 0.781 | 0.599 | 0.608 |
| GGGGCTTTGC | 0.045 | 0.851 | 0.833 | 0.713 | GGGTCTTCCT | -0.699 | 0.802 | 0.778 | 0.729 |
| AGTGTTTTTC | 0.045 | 0.769 | 0.578 | 0.447 | GGTGTTCTCA | -0.699 | 0.765 | 0.723 | 0.598 |
| GGGATTTGCG | 0.045 | 0.777 | 0.838 | 0.657 | GGTATATTCT | -0.699 | 0.766 | 0.627 | 0.454 |
| GTTTCTTTCC | 0.045 | 0.755 | 0.614 | 0.491 | GGTGTTATCG | -0.699 | 0.750 | 0.771 | 0.585 |
| TGGTATTCTC | 0.045 | 0.751 | 0.654 | 0.646 | TGTATTTTCT | -0.699 | 0.755 | 0.577 | 0.396 |
| TTGAGTTTCC | 0.041 | 0.768 | 0.619 | 0.497 | GTTGGTTCAC | -0.699 | 0.811 | 0.530 | 0.519 |
| GGCTGTTTCC | 0.041 | 0.782 | 0.672 | 0.619 | GGTGCTTCGT | -0.721 | 0.753 | 0.617 | 0.551 |
| TGTAGTTTCC | 0.041 | 0.849 | 0.619 | 0.497 | GTTGATTTTC | -0.721 | 0.763 | 0.601 | 0.524 |
| GGGTGTGTCC | 0.041 | 0.824 | 0.792 | 0.727 | GGTTGTGTAC | -0.745 | 0.757 | 0.539 | 0.461 |
| GTTATTTCCG | 0.041 | 0.787 | 0.677 | 0.480 | CGTGGTTCTC | -0.745 | 0.782 | 0.530 | 0.519 |
| GTGGTTTGCC | 0.037 | 0.754 | 0.758 | 0.702 | GGTGTGTGCC | -0.745 | 0.747 | 0.749 | 0.642 |
| GGTGCTTCCT | 0.033 | 0.847 | 0.744 | 0.684 | GGTGATGTCT | -0.745 | 0.772 | 0.739 | 0.658 |
| TTGGGTTCCC | 0.033 | 0.784 | 0.657 | 0.652 | GTTGCTTCGC | -0.745 | 0.753 | 0.599 | 0.524 |
| GGGAGTTTGT | 0.025 | 0.767 | 0.637 | 0.524 | GGTCTTTCGC | -0.745 | 0.782 | 0.631 | 0.569 |
| CGTTTTTCCC | 0.025 | 0.798 | 0.631 | 0.569 | GGTAGTTGTC | -0.745 | 0.763 | 0.545 | 0.486 |
| TGTTATTTCC | 0.021 | 0.778 | 0.635 | 0.568 | GGTCGTTTCG | -0.745 | 0.753 | 0.645 | 0.519 |
| GAGGTTTTTC | 0.021 | 0.776 | 0.705 | 0.580 | GTTAGTTTTC | -0.745 | 0.768 | 0.492 | 0.364 |
| GGTGTTTTAG | 0.017 | 0.807 | 0.677 | 0.480 | CGTTCTTTCC | -0.745 | 0.754 | 0.614 | 0.491 |
| AGGGATTTAC | 0.000 | 0.814 | 0.727 | 0.657 | GGTTGTCTCC | -0.745 | 0.779 | 0.672 | 0.611 |
| GGGAGCTTAC | 0.000 | 0.814 | 0.669 | 0.556 | GGTGGTTAGC | -0.770 | 0.753 | 0.565 | 0.564 |
| GGAGTTTCTA | 0.000 | 0.759 | 0.615 | 0.551 | TGTAGTTCCG | -0.770 | 0.786 | 0.611 | 0.475 |
| CGGGATGCCC | 0.000 | 0.818 | 0.867 | 0.842 | GTGGCTTCCG | -0.770 | 0.780 | 0.825 | 0.690 |
| TGGACTTCTC | 0.000 | 0.813 | 0.707 | 0.580 | GGTGAGTTCG | -0.770 | 0.762 | 0.817 | 0.630 |
| GGAGTTCCTC | 0.000 | 0.760 | 0.723 | 0.649 | GGGTCTGCTC | -0.770 | 0.767 | 0.754 | 0.676 |
| GGGTTTGCCA | 0.000 | 0.770 | 0.770 | 0.703 | GGGGTCTCCT | -0.770 | 0.782 | 0.792 | 0.742 |
| GGTAGGTCCG | 0.000 | 0.793 | 0.728 | 0.548 | GGGGTTGCTA | -0.770 | 0.763 | 0.736 | 0.659 |
| GGTACTTTCA | 0.000 | 0.846 | 0.706 | 0.529 | GGTGCGTCTC | -0.770 | 0.789 | 0.716 | 0.597 |
| GGGGTTTCAA | 0.000 | 0.855 | 0.742 | 0.684 | GTGACTTTCG | -0.770 | 0.764 | 0.787 | 0.536 |
| GGTACGGTCC | 0.000 | 0.782 | 0.798 | 0.550 | GTGGCTGTCC | -0.770 | 0.779 | 0.827 | 0.687 |
| GGGTTTACAC | 0.000 | 0.773 | 0.725 | 0.673 | GGTGTTGCAG | -0.770 | 0.769 | 0.690 | 0.532 |
| GGCAGTACCC | 0.000 | 0.781 | 0.731 | 0.679 | GGTTGTGCCC | -0.770 | 0.829 | 0.685 | 0.671 |
| GGCATTTCTC | 0.000 | 0.820 | 0.705 | 0.580 | GTTGAGTTCC | -0.770 | 0.749 | 0.718 | 0.597 |
| CATGATTCCC | 0.000 | 0.747 | 0.620 | 0.601 | GTTGATTCCG | -0.770 | 0.773 | 0.719 | 0.634 |
| TGGAGTGCTC | 0.000 | 0.758 | 0.631 | 0.549 | TGTATTTTAC | -0.770 | 0.811 | 0.559 | 0.369 |
| GGAACTTTCT | 0.000 | 0.785 | 0.706 | 0.529 | GGTGGTCCTC | -0.770 | 0.798 | 0.657 | 0.644 |
| GTGGCTTTAC | 0.000 | 0.797 | 0.707 | 0.580 | CGTGGTTCGC | -0.770 | 0.761 | 0.530 | 0.519 |
| GAGGATTTTC | 0.000 | 0.773 | 0.727 | 0.657 | GGCGTTGTCC | -0.770 | 0.793 | 0.825 | 0.687 |
| CGTTGTTCCC | 0.000 | 0.789 | 0.565 | 0.564 | GGTGTCTCGC | -0.770 | 0.770 | 0.647 | 0.582 |
| CGGATTGCCC | 0.000 | 0.831 | 0.825 | 0.687 | GTTAATTTCG | -0.770 | 0.758 | 0.681 | 0.479 |
| GTGATATCAC | 0.000 | 0.767 | 0.628 | 0.505 | GGCGCTTTCG | -0.770 | 0.750 | 0.806 | 0.613 |
| GGCGGTCCCC | 0.000 | 0.786 | 0.783 | 0.777 | GGTACGTTTC | -0.770 | 0.773 | 0.678 | 0.443 |
| CATGTTTCCC | 0.000 | 0.750 | 0.597 | 0.524 | GTTGGGTCCC | -0.770 | 0.769 | 0.647 | 0.592 |
| GGGCATATCC | 0.000 | 0.764 | 0.855 | 0.806 | GGTAGTGTCG | -0.770 | 0.791 | 0.712 | 0.505 |
| CAGGGTTCCC | 0.000 | 0.763 | 0.657 | 0.652 | GGTATTGCCT | -0.770 | 0.811 | 0.716 | 0.581 |
| GGGGTATCCG | 0.000 | 0.818 | 0.873 | 0.749 | GTGGGTCTCC | -0.770 | 0.764 | 0.764 | 0.699 |
| GGTGCTCCCA | 0.000 | 0.773 | 0.744 | 0.676 | GTTGCTTCCG | -0.770 | 0.759 | 0.698 | 0.557 |
| GGTAATTTCA | 0.000 | 0.861 | 0.726 | 0.606 | GGCGTTTTCG | -0.770 | 0.768 | 0.804 | 0.613 |
| GGTATGTTAC | 0.000 | 0.818 | 0.676 | 0.443 | GGTGGGTTCG | -0.770 | 0.757 | 0.728 | 0.548 |
| AATGATTCCC | 0.000 | 0.751 | 0.620 | 0.601 | GGTAGTTCCT | -0.770 | 0.867 | 0.656 | 0.602 |
| GGTAGTCTCC | 0.000 | 0.856 | 0.745 | 0.622 | GGTTAGTTCC | -0.770 | 0.785 | 0.752 | 0.641 |
| TGTAATCCCC | 0.000 | 0.791 | 0.727 | 0.648 | GTGGGTTCCT | -0.796 | 0.775 | 0.675 | 0.679 |
| GGGCCTTCCT | 0.000 | 0.776 | 0.778 | 0.729 | CGTGCTTTTC | -0.796 | 0.747 | 0.580 | 0.447 |
| GGAGTTTCGC | 0.000 | 0.827 | 0.724 | 0.657 | GGTTCGTTCC | -0.824 | 0.770 | 0.731 | 0.565 |
| GGGTCCTCCC | 0.000 | 0.801 | 0.810 | 0.760 | GGTGCGTTCG | -0.824 | 0.747 | 0.796 | 0.553 |
| AGGGTTCTCC | 0.000 | 0.775 | 0.831 | 0.704 | GTTGCTTTTC | -0.824 | 0.748 | 0.580 | 0.447 |
| GGTAAGGTAC | 0.000 | 0.750 | 0.692 | 0.494 | GTGAGTTTCT | -0.824 | 0.759 | 0.637 | 0.524 |
| GGTAAGTTTC | 0.000 | 0.788 | 0.698 | 0.519 | GTTAGTTTCG | -0.824 | 0.752 | 0.591 | 0.397 |
| GTGACGTCCC | 0.000 | 0.791 | 0.824 | 0.653 | GGTATCTTCG | -0.854 | 0.759 | 0.708 | 0.461 |
| GGTATTATCG | 0.000 | 0.760 | 0.752 | 0.507 | GCTGTTTTAC | -0.854 | 0.769 | 0.578 | 0.447 |
| GGAACATTCC | 0.000 | 0.805 | 0.737 | 0.561 | GGGAGTTCTG | -0.854 | 0.828 | 0.737 | 0.608 |
| TGGAGGTCCC | 0.000 | 0.808 | 0.755 | 0.648 | CGGGTTTCCG | -0.854 | 0.796 | 0.823 | 0.690 |
| TATATTTCCC | 0.000 | 0.769 | 0.578 | 0.447 | GGGGGTTCGG | -0.854 | 0.797 | 0.757 | 0.685 |
| GGGGTCCTCC | 0.000 | 0.771 | 0.881 | 0.763 | GGTGGTCTCG | -0.854 | 0.756 | 0.737 | 0.599 |
| GAAGTTTTCC | 0.000 | 0.782 | 0.705 | 0.580 | GGTACTGTCT | -0.854 | 0.767 | 0.699 | 0.504 |
| TGGGCGTTCC | 0.000 | 0.763 | 0.824 | 0.653 | GGTCGTTCCG | -0.854 | 0.779 | 0.664 | 0.597 |
| GTGATTGTCC | 0.000 | 0.807 | 0.806 | 0.610 | GGGGCTGTGC | -0.854 | 0.787 | 0.827 | 0.687 |
| TGGATTGTCC | 0.000 | 0.815 | 0.806 | 0.610 | GTGGCTTTGC | -0.854 | 0.749 | 0.707 | 0.580 |
| GGGAAGTTGC | 0.000 | 0.788 | 0.825 | 0.652 | GGGATGTCTG | -0.854 | 0.749 | 0.794 | 0.553 |
| GTGAATTCAC | 0.000 | 0.847 | 0.727 | 0.657 | AGTATTTTCT | -0.854 | 0.749 | 0.577 | 0.396 |
| GGAACTTCTA | 0.000 | 0.751 | 0.598 | 0.474 | GGGAGTTCGT | -0.886 | 0.793 | 0.656 | 0.602 |
| GGGACTCTCG | 0.000 | 0.778 | 0.913 | 0.660 | GGAGGTTTCT | -0.886 | 0.784 | 0.656 | 0.602 |
| GGGAGTTCTA | 0.000 | 0.829 | 0.656 | 0.602 | TGTAATTTCT | -0.886 | 0.751 | 0.600 | 0.473 |
| GGGGCATCCT | 0.000 | 0.785 | 0.794 | 0.742 | GGTAGTTTGG | -0.886 | 0.760 | 0.591 | 0.397 |
| GGGATTCTCA | 0.000 | 0.796 | 0.830 | 0.654 | CGTGTTTTCA | -0.886 | 0.750 | 0.596 | 0.474 |
| GGTGCGCCCC | 0.000 | 0.774 | 0.843 | 0.722 | GTGGGTTTCG | -0.886 | 0.764 | 0.737 | 0.608 |
| GAGGTTTTAC | 0.000 | 0.803 | 0.705 | 0.580 | GATTGTTTCC | -0.886 | 0.754 | 0.545 | 0.486 |
| GGGAATCTCG | 0.000 | 0.793 | 0.934 | 0.737 | GGTTGGTTCC | -0.921 | 0.780 | 0.663 | 0.559 |
| GGGGCATTCA | 0.000 | 0.774 | 0.775 | 0.665 | GGTGTGTTGC | -0.959 | 0.760 | 0.695 | 0.520 |
| GGGGTTCGCC | 0.000 | 0.768 | 0.885 | 0.826 | GTTGTTTCAC | -0.959 | 0.820 | 0.597 | 0.524 |
| GGGTTATCAC | 0.000 | 0.794 | 0.681 | 0.627 | GGTGCTGTCT | -0.959 | 0.757 | 0.718 | 0.581 |
| CGTGTATTCC | 0.000 | 0.755 | 0.628 | 0.505 | GCGGTTTCTC | -0.959 | 0.788 | 0.724 | 0.657 |
| TGGGTTTCCA | 0.000 | 0.806 | 0.742 | 0.684 | GGTGTATTGC | -0.959 | 0.765 | 0.628 | 0.505 |
| GGGGGCTCCT | 0.000 | 0.773 | 0.725 | 0.737 | GGTAGTTTAT | -0.959 | 0.795 | 0.510 | 0.391 |
| GGGAATTCAG | 0.000 | 0.861 | 0.827 | 0.690 | GGGCCTTTCT | -0.959 | 0.750 | 0.759 | 0.651 |
| CGTGGTCCCC | 0.000 | 0.767 | 0.657 | 0.644 | GGTCATTTTC | -0.959 | 0.774 | 0.635 | 0.568 |
| TGGATTTCCA | 0.000 | 0.816 | 0.723 | 0.607 | GGCGGTTTGC | -0.959 | 0.754 | 0.638 | 0.575 |
| GATTATTCCC | 0.000 | 0.785 | 0.654 | 0.646 | GTTGTTTTCA | -0.959 | 0.751 | 0.596 | 0.474 |
| TGAAGTTCCC | 0.000 | 0.828 | 0.638 | 0.575 | GGTGTGTTCA | -1.000 | 0.766 | 0.713 | 0.547 |
| GGTATTTCAG | 0.000 | 0.843 | 0.677 | 0.480 | AGTTGTTTCC | -1.000 | 0.767 | 0.545 | 0.486 |
| GGAGGTTTCC | 0.000 | 0.888 | 0.765 | 0.708 | GGTGTGTCTC | -1.000 | 0.807 | 0.714 | 0.597 |
| CGTTTTTCAC | 0.000 | 0.751 | 0.505 | 0.436 | GTGGGTTCGC | -1.000 | 0.784 | 0.657 | 0.652 |
| GGTGATTATC | 0.000 | 0.779 | 0.654 | 0.646 | GGGGTGTCTC | -1.000 | 0.828 | 0.841 | 0.730 |
| TGCGATTTCC | 0.000 | 0.759 | 0.727 | 0.657 | GTTGATTTCG | -1.000 | 0.748 | 0.700 | 0.557 |
| ATGGGTTCCC | 0.000 | 0.778 | 0.657 | 0.652 | GGTATTGTGC | -1.000 | 0.794 | 0.679 | 0.477 |
| TGATGTTCCC | 0.000 | 0.752 | 0.565 | 0.564 | GGTTGTTCAG | -1.046 | 0.758 | 0.537 | 0.464 |
| TGTAATTGCC | 0.000 | 0.747 | 0.635 | 0.568 | GGTGTTGTCA | -1.046 | 0.790 | 0.716 | 0.581 |
| GGGGCCTCTC | 0.000 | 0.793 | 0.776 | 0.715 | GGTAGTGTCT | -1.046 | 0.777 | 0.630 | 0.499 |
| GGGGATTTAA | 0.000 | 0.825 | 0.746 | 0.683 | GGTATTGTCG | -1.046 | 0.799 | 0.778 | 0.510 |
| GGGATGTTAC | 0.000 | 0.839 | 0.803 | 0.576 | GGTAGTTTGC | -1.046 | 0.850 | 0.619 | 0.497 |
| GGCATTTCCA | 0.000 | 0.804 | 0.723 | 0.607 | GTTGGTTCGC | -1.097 | 0.763 | 0.530 | 0.519 |
| GGGGGCTCAC | 0.000 | 0.830 | 0.707 | 0.710 | GGGCGTTTGC | -1.097 | 0.769 | 0.672 | 0.619 |
| TGGAATTACC | 0.000 | 0.789 | 0.762 | 0.701 | GGTCGTTTGC | -1.097 | 0.747 | 0.545 | 0.486 |
| GGTAGATTAC | 0.000 | 0.815 | 0.542 | 0.423 | GGTGGTCTGC | -1.097 | 0.751 | 0.638 | 0.566 |
| GGATGTTCTC | 0.000 | 0.773 | 0.565 | 0.564 | TGGGGTTCCG | -1.155 | 0.797 | 0.757 | 0.685 |
| GTTGATTCCC | 0.000 | 0.863 | 0.746 | 0.734 | GGTGCTTCGG | -1.222 | 0.767 | 0.698 | 0.557 |
| GGTTCTTCCA | 0.000 | 0.795 | 0.652 | 0.596 | GTTACTTTTC | -1.222 | 0.758 | 0.561 | 0.369 |
| GGTCTTTTAC | 0.000 | 0.804 | 0.612 | 0.491 | GTTGGTTTAC | -1.301 | 0.785 | 0.511 | 0.442 |
| GGGTAGTCTC | 0.000 | 0.758 | 0.771 | 0.719 | GTTGGTTCTC | -1.398 | 0.784 | 0.530 | 0.519 |

Table S4. The SELEX-Seq values and model scores of 114 sequences.

| Sequence | SELEX | SNMM | PWMSA | Match | PC |
| --- | --- | --- | --- | --- | --- |
| GGGATTTCCC | 2.450 | 1.000 | 0.958 | 0.846 | 0.891 |
| GGGGATTTCC | 2.382 | 0.961 | 0.981 | 0.923 | 0.902 |
| GGGGTTTCCC | 2.348 | 0.990 | 0.977 | 0.923 | 0.969 |
| GGGGATTCCC | 2.342 | 0.987 | 1.000 | 1.000 | 0.992 |
| GGGATTCCCC | 2.333 | 0.911 | 0.958 | 0.837 | 0.973 |
| GGGGTTCCCC | 2.289 | 0.901 | 0.977 | 0.915 | 0.957 |
| GGGGTTTTCC | 2.280 | 0.964 | 0.958 | 0.846 | 0.953 |
| GGGGGTTTCC | 2.199 | 0.956 | 0.891 | 0.841 | 0.781 |
| GGGGGTTCCC | 2.180 | 0.982 | 0.911 | 0.918 | 0.910 |
| GGGACTTTCC | 2.165 | 0.956 | 0.941 | 0.768 | 0.371 |
| GGGAGTTCCC | 2.158 | 0.991 | 0.891 | 0.841 | 0.734 |
| GGGAATTCCC | 2.143 | 0.997 | 0.981 | 0.923 | 0.930 |
| GGGGCTTTCC | 2.135 | 0.946 | 0.960 | 0.846 | 0.746 |
| GGGGCTTCCC | 2.113 | 0.972 | 0.979 | 0.923 | 0.981 |
| GGGATTTTCC | 2.076 | 0.974 | 0.939 | 0.768 | 0.606 |
| GGGACTTCCC | 2.057 | 0.982 | 0.960 | 0.846 | 0.844 |
| GGGATGTCCC | 2.029 | 0.912 | 0.949 | 0.786 | 0.793 |
| GGGGTGTCCC | 2.016 | 0.902 | 0.968 | 0.863 | 0.859 |
| GGGGATCCCC | 1.954 | 0.898 | 1.000 | 0.991 | 0.988 |
| GGGGAATTCC | 1.929 | 0.878 | 0.904 | 0.848 | 0.820 |
| GGGGAGTTCC | 1.914 | 0.873 | 0.971 | 0.863 | 0.723 |
| GGGATCTCCC | 1.900 | 0.895 | 0.881 | 0.771 | 0.813 |
| GGGGATCTCC | 1.900 | 0.872 | 0.980 | 0.914 | 0.613 |
| GGGGGATTCC | 1.877 | 0.872 | 0.815 | 0.766 | 0.856 |
| GGAGTTTCCC | 1.871 | 0.922 | 0.851 | 0.790 | 0.543 |
| GGAGTTCCCC | 1.842 | 0.833 | 0.850 | 0.782 | 0.656 |
| GGGAGTTTCC | 1.837 | 0.966 | 0.872 | 0.763 | 0.406 |
| GGGGACTCCC | 1.810 | 0.882 | 0.923 | 0.925 | 0.887 |
| GGGGACTTCC | 1.712 | 0.856 | 0.904 | 0.848 | 0.762 |
| GGGGTTCTCC | 1.708 | 0.875 | 0.958 | 0.837 | 0.625 |
| GGGGCGTCCC | 1.703 | 0.884 | 0.970 | 0.863 | 0.832 |
| GGGACGTCCC | 1.699 | 0.894 | 0.951 | 0.786 | 0.609 |
| GGGGGATCCC | 1.690 | 0.898 | 0.834 | 0.843 | 0.773 |
| GGGATTCTCC | 1.690 | 0.885 | 0.939 | 0.760 | 0.430 |
| GGGGCATCCC | 1.681 | 0.889 | 0.903 | 0.848 | 0.867 |
| GGGAATTTCC | 1.658 | 0.971 | 0.962 | 0.845 | 0.641 |
| GGGGTATTCC | 1.651 | 0.881 | 0.881 | 0.771 | 0.879 |
| GGGGCATTCC | 1.643 | 0.863 | 0.883 | 0.771 | 0.711 |
| GGGGTCTCCC | 1.620 | 0.885 | 0.901 | 0.848 | 0.809 |
| GGAATTTCCC | 1.607 | 0.932 | 0.831 | 0.713 | 0.629 |
| GGGGTATCCC | 1.602 | 0.907 | 0.901 | 0.848 | 0.914 |
| GGGGAGCCCC | 1.580 | 0.810 | 0.990 | 0.932 | 0.926 |
| GGGGGCTTCC | 1.522 | 0.851 | 0.815 | 0.766 | 0.680 |
| GGGGCTCTCC | 1.505 | 0.857 | 0.960 | 0.837 | 0.574 |
| GGGGCGCCCC | 1.491 | 0.795 | 0.969 | 0.855 | 0.699 |
| GGAGATTCCC | 1.477 | 0.919 | 0.873 | 0.867 | 0.582 |
| GGGGTGCCCC | 1.462 | 0.813 | 0.967 | 0.855 | 0.766 |
| GGGGGGTTCC | 1.461 | 0.867 | 0.882 | 0.781 | 0.559 |
| GGGGGTCCCC | 1.439 | 0.893 | 0.910 | 0.910 | 0.941 |
| GGGAGATTCC | 1.431 | 0.882 | 0.795 | 0.688 | 0.566 |
| GGGATATCCC | 1.415 | 0.917 | 0.881 | 0.771 | 0.828 |
| GGGGGTCTCC | 1.415 | 0.867 | 0.891 | 0.832 | 0.481 |
| GGGGGGTCCC | 1.407 | 0.893 | 0.901 | 0.858 | 0.727 |
| GGAGAGTCCC | 1.362 | 0.831 | 0.864 | 0.807 | 0.277 |
| GGAGTCCCCC | 1.342 | 0.729 | 0.773 | 0.707 | 0.473 |
| GGGGGCTCCC | 1.312 | 0.877 | 0.834 | 0.843 | 0.684 |
| GGGAAGTTCC | 1.279 | 0.883 | 0.952 | 0.785 | 0.465 |
| GGGGGACCCC | 1.267 | 0.809 | 0.833 | 0.835 | 0.797 |
| GGAAGTTCCC | 1.255 | 0.923 | 0.765 | 0.708 | 0.496 |
| GGGACATTCC | 1.217 | 0.873 | 0.864 | 0.694 | 0.508 |
| GGGGTCTTCC | 1.209 | 0.860 | 0.881 | 0.771 | 0.707 |
| GGAGTTTTCC | 1.188 | 0.896 | 0.831 | 0.713 | 0.445 |
| GGGAGGTCCC | 1.176 | 0.903 | 0.882 | 0.781 | 0.652 |
| GGAGTGTCCC | 1.146 | 0.834 | 0.841 | 0.730 | 0.344 |
| GGGGTGTTCC | 1.138 | 0.876 | 0.949 | 0.786 | 0.668 |
| GGGGCGTTCC | 1.136 | 0.858 | 0.951 | 0.786 | 0.586 |
| GGGGCCTCCC | 1.130 | 0.867 | 0.903 | 0.848 | 0.754 |
| GGGGCCTTCC | 1.114 | 0.841 | 0.883 | 0.771 | 0.520 |
| GGAATTTTCC | 1.114 | 0.906 | 0.812 | 0.635 | 0.164 |
| GGGATATTCC | 1.097 | 0.891 | 0.862 | 0.694 | 0.535 |
| GGGATCTTCC | 1.079 | 0.870 | 0.862 | 0.694 | 0.418 |
| GGGGTACCCC | 0.954 | 0.818 | 0.900 | 0.840 | 0.840 |
| GGGATGTTCC | 0.938 | 0.886 | 0.929 | 0.709 | 0.453 |
| GGAGACTCCC | 0.903 | 0.814 | 0.796 | 0.792 | 0.297 |
| GGGAGACTCC | 0.875 | 0.793 | 0.795 | 0.680 | 0.258 |
| GGGACGTTCC | 0.813 | 0.868 | 0.931 | 0.709 | 0.313 |
| GGGACCTTCC | 0.813 | 0.851 | 0.864 | 0.694 | 0.363 |
| GGGGGGCCCC | 0.778 | 0.804 | 0.901 | 0.850 | 0.691 |
| GGGGACCTCC | 0.778 | 0.767 | 0.904 | 0.839 | 0.551 |
| GGAGTGCCCC | 0.778 | 0.745 | 0.841 | 0.722 | 0.504 |
| GGGGGCCTCC | 0.699 | 0.762 | 0.814 | 0.757 | 0.324 |
| GGGGGCCCCC | 0.699 | 0.788 | 0.833 | 0.835 | 0.664 |
| GGAAATTTCC | 0.602 | 0.903 | 0.835 | 0.712 | 0.184 |
| GGGGGGCTCC | 0.602 | 0.779 | 0.881 | 0.772 | 0.391 |
| GGGGCGCTCC | 0.602 | 0.769 | 0.950 | 0.777 | 0.328 |
| GGAAAGTTCC | 0.602 | 0.815 | 0.825 | 0.652 | 0.035 |
| GGAGATTTCC | 0.544 | 0.893 | 0.854 | 0.790 | 0.242 |
| GGGGAGCTCC | 0.477 | 0.784 | 0.971 | 0.854 | 0.594 |
| GGGAGCTCCC | 0.477 | 0.887 | 0.815 | 0.766 | 0.637 |
| GGAGTACCCC | 0.477 | 0.750 | 0.773 | 0.707 | 0.488 |
| GGGGTGCTCC | 0.427 | 0.787 | 0.948 | 0.777 | 0.422 |
| GGAGCTTCCC | 0.398 | 0.904 | 0.853 | 0.790 | 0.527 |
| GGGACCCTCC | 0.398 | 0.762 | 0.864 | 0.685 | 0.176 |
| GGAATTCTCC | 0.398 | 0.817 | 0.812 | 0.627 | 0.199 |
| GGGAGCTTCC | 0.301 | 0.861 | 0.795 | 0.688 | 0.356 |
| GGAGCTTTCC | 0.176 | 0.878 | 0.833 | 0.713 | 0.125 |
| GGGGCCCTCC | 0.176 | 0.752 | 0.883 | 0.763 | 0.402 |
| GGAGGCCTCC | 0.000 | 0.694 | 0.687 | 0.624 | 0.000 |
| GGAACATTCC | 0.000 | 0.805 | 0.737 | 0.561 | 0.063 |
| GGGGTCCTCC | 0.000 | 0.771 | 0.881 | 0.763 | 0.379 |
| GGAGGTTTCC | 0.000 | 0.888 | 0.765 | 0.708 | 0.106 |
| GGGATCCTCC | 0.000 | 0.781 | 0.862 | 0.685 | 0.156 |
| GGGACGCTCC | 0.000 | 0.779 | 0.931 | 0.700 | 0.250 |
| GGAGTATTCC | 0.000 | 0.813 | 0.755 | 0.638 | 0.285 |
| GGAGTGTTCC | -0.174 | 0.808 | 0.822 | 0.653 | 0.219 |
| GGAGTTCTCC | -0.301 | 0.807 | 0.831 | 0.704 | 0.293 |
| GGAGTGCTCC | -0.301 | 0.719 | 0.821 | 0.645 | 0.234 |
| GGAACCTCCC | -0.301 | 0.809 | 0.757 | 0.638 | 0.383 |
| GGAGTCTTCC | -0.301 | 0.792 | 0.755 | 0.638 | 0.148 |
| GGAAGTTTCC | -0.301 | 0.898 | 0.746 | 0.630 | 0.090 |
| GGGAACCTCC | -0.301 | 0.777 | 0.884 | 0.762 | 0.309 |
| GGGATGCTCC | -0.301 | 0.797 | 0.929 | 0.700 | 0.336 |
| GGAACTCTCC | -0.301 | 0.799 | 0.814 | 0.627 | 0.109 |
| GGAACCCTCC | -0.301 | 0.694 | 0.737 | 0.552 | 0.027 |

Table S5. The SELEX-Seq values and model scores of 25 sequences.

| Sequence | SELEX | SNMM | PWMSA | Match | PC | EMSA |
| --- | --- | --- | --- | --- | --- | --- |
| GGGGATTCCC | 2.342 | 0.987 | 1.000 | 1.000 | 0.992 | 2.704 |
| GGGGTTCCCC | 2.289 | 0.901 | 0.977 | 0.915 | 0.957 | 2.356 |
| GGGGTTTTCC | 2.280 | 0.964 | 0.958 | 0.846 | 0.953 | 2.322 |
| GGGGCTTCCC | 2.113 | 0.972 | 0.979 | 0.923 | 0.981 | 2.444 |
| GGGATGTCCC | 2.029 | 0.912 | 0.949 | 0.786 | 0.793 | 1.732 |
| GGGATCTCCC | 1.900 | 0.895 | 0.881 | 0.771 | 0.813 | 2.057 |
| GGGGGATTCC | 1.877 | 0.872 | 0.815 | 0.766 | 0.856 | 2.336 |
| GGGGACTTCC | 1.712 | 0.856 | 0.904 | 0.848 | 0.762 | 2.155 |
| GGGAATTTCC | 1.658 | 0.971 | 0.962 | 0.845 | 0.641 | 2.328 |
| GGGGTATCCC | 1.602 | 0.907 | 0.901 | 0.848 | 0.914 | 1.602 |
| GGGGAGCCCC | 1.580 | 0.810 | 0.990 | 0.932 | 0.926 | 1.756 |
| GGGGGCTTCC | 1.522 | 0.851 | 0.815 | 0.766 | 0.680 | 1.826 |
| GGGATATCCC | 1.415 | 0.917 | 0.881 | 0.771 | 0.828 | 1.623 |
| GGGGGCTCCC | 1.312 | 0.877 | 0.834 | 0.843 | 0.684 | 1.929 |
| GGGAGACTCC | 0.875 | 0.793 | 0.795 | 0.680 | 0.258 | 0.301 |
| GGGGGCCCCC | 0.699 | 0.788 | 0.833 | 0.835 | 0.664 | 1.431 |
| GGAAATTTCC | 0.602 | 0.903 | 0.835 | 0.712 | 0.184 | 0.778 |
| GGGGGGCTCC | 0.602 | 0.779 | 0.881 | 0.772 | 0.391 | 1.301 |
| GGGAGCTCCC | 0.477 | 0.887 | 0.815 | 0.766 | 0.637 | 0.699 |
| GGGGTGCTCC | 0.427 | 0.787 | 0.948 | 0.777 | 0.422 | 0.699 |
| GGAATTCTCC | 0.398 | 0.817 | 0.812 | 0.627 | 0.199 | 0.477 |
| GGAGGCCTCC | 0.000 | 0.694 | 0.687 | 0.624 | 0.000 | 0.000 |
| GGGATCCTCC | 0.000 | 0.781 | 0.862 | 0.685 | 0.156 | 0.477 |
| GGAACCTCCC | -0.301 | 0.809 | 0.757 | 0.638 | 0.383 | 0.301 |
| GGAAGTTTCC | -0.301 | 0.898 | 0.746 | 0.630 | 0.090 | 0.301 |

Table S6. The sequences used to determine the optimal threshold of SNMM.

| S1 | | | | S2 | | |
| --- | --- | --- | --- | --- | --- | --- |
|  | EMSA | SNMM | Match |  | SNMM | Match |
| GGGGATTCCC | 2.704 | 0.987 | 1 | CCTTGCGGGG | 0.174 | 0 |
| GGGGAATCCC | 2.614 | 0.903 | 0.925 | ATATGGTGTT | 0.245 | 0 |
| GGGGCTTCCC | 2.444 | 0.972 | 0.923 | TATTGGGGGC | 0.303 | 0 |
| GGGGTTCCCC | 2.356 | 0.901 | 0.915 | TTATGGGTGT | 0.272 | 0 |
| GGGGGATTCC | 2.336 | 0.872 | 0.766 | ACCTGGTGGT | 0.160 | 0 |
| GGGAATTTCC | 2.328 | 0.971 | 0.845 | TTTTGGTTGT | 0.384 | 0 |
| GGGGTTTTCC | 2.322 | 0.964 | 0.846 | TATCGTGGGG | 0.276 | 0 |
| GGGAAATTCC | 2.258 | 0.888 | 0.77 | CCCTGGGGCT | 0.186 | 0 |
| GGGGACTTCC | 2.155 | 0.856 | 0.848 | TCATGGTGGT | 0.204 | 0 |
| GGGATCTCCC | 2.057 | 0.895 | 0.771 | TTCTGTGGTT | 0.235 | 0 |
| GGGGAGTCCC | 1.991 | 0.898 | 0.94 | TCTTGGTGGG | 0.265 | 0 |
| GGGGGCTCCC | 1.929 | 0.877 | 0.843 | CTTTGGGGGA | 0.216 | 0 |
| GGGGGCTTCC | 1.826 | 0.851 | 0.766 | ATTCGGGGGA | 0.195 | 0 |
| GGGATACCCC | 1.799 | 0.828 | 0.763 | TTTTGGTACT | 0.391 | 0 |
| GGGGAGCCCC | 1.756 | 0.81 | 0.932 | ACTCGTGGGG | 0.257 | 0 |
| GGGGACCCCC | 1.748 | 0.793 | 0.917 | ACCTGGTGTT | 0.181 | 0 |
| GGGATGTCCC | 1.732 | 0.912 | 0.786 | ACTTGGGGGA | 0.195 | 0 |
| GGGGCTCCCC | 1.69 | 0.883 | 0.915 | CCCCGGTGCT | 0.225 | 0 |
| GGGAACTTCC | 1.663 | 0.866 | 0.77 | TTACGGGGGG | 0.153 | 0 |
| GGAATACCCC | 1.653 | 0.76 | 0.63 | CCATGGGGTT | 0.151 | 0 |
| GGGATATCCC | 1.623 | 0.917 | 0.771 | CTCTGGGGTG | 0.151 | 0 |
| GGGGTATCCC | 1.602 | 0.907 | 0.848 | ACCTGGGGTT | 0.116 | 0 |
| GGGGCACCCC | 1.491 | 0.8 | 0.84 | CTTCGGGGGC | 0.280 | 0 |
| GGGGCCCCCC | 1.477 | 0.778 | 0.84 | ACTTGGGACT | 0.296 | 0 |
| GGGAGGCCCC | 1.447 | 0.814 | 0.772 | ATCCGTGGGG | 0.197 | 0 |
| GGGACTCTCC | 1.431 | 0.867 | 0.76 | CTCTGCGGTG | 0.135 | 0 |
| GGGGGCCCCC | 1.431 | 0.788 | 0.835 | TTTTGGTTTT | 0.405 | 0 |
| GGAGAACCCC | 1.415 | 0.747 | 0.784 | CATCGGTGGT | 0.229 | 0 |
| GGGGGGCTCC | 1.301 | 0.779 | 0.772 | CATCGTGGGG | 0.267 | 0 |
| GGAGGGTTCC | 0.954 | 0.8 | 0.648 | TACCGGTGTT | 0.174 | 0 |

**References**

1. Siggers T, Chang AB, Teixeira A, Wong D, Williams KJ, et al. (2012) Principles of dimer-specific gene regulation revealed by a comprehensive characterization of NF-kappaB family DNA binding. Nat Immunol 13: 95-102.

2. Aleksandrushkina NI, Egorova LA (1978) [Nucleotide makeup of the DNA of thermophilic bacteria of the genus Thermus]. Mikrobiologiia 47: 250-252.

3. Ben-Gal I, Shani A, Gohr A, Grau J, Arviv S, et al. (2005) Identification of transcription factor binding sites with variable-order Bayesian networks. Bioinformatics 21: 2657-2666.
